# Sensory-processing sensitivity versus the sensory-processing theory: Convergence and divergence

**DOI:** 10.3389/fpsyg.2022.1010836

**Published:** 2022-12-01

**Authors:** Yaara Turjeman-Levi, Avraham N. Kluger

**Affiliations:** The Hebrew University Business School, The Hebrew University of Jerusalem, Jerusalem, Israel

**Keywords:** sensory-processing sensitivity, adolescent adult sensory profile, exploratory graph analysis, neuroticism, validity

## Abstract

Two individual-difference theories focus on sensory sensitivity: one emanating from psychology—sensory-processing-sensitivity (SPS); and one from occupational therapy—sensory processing theory (SP). Each theory is coupled with its measure: the highly-sensitive-person scale (HSPS) and the adolescent adult sensory profile (ASP). The constructs of both theories were claimed to be independent of neuroticism. To assess the convergence of these measures, we recruited participants from a general population and a Facebook Group dedicated to people high in SPS. The participants, *N* = 1,702 *M*_age_ = 26.9 (66.7% female), answered the HSPS, ASP, and neuroticism questionnaires. We subjected the HSPS and the APS to exploratory graph analysis. To assess the divergence of these measures from neuroticism, we performed meta-analyses. We also used a subsample obtained in an unrelated study, *N =* 490, to correlate HSPS and APS with the Big Five and additional measures. The results suggested that (a) the latent structure of these measures conforms to the theories only partially, (b) some of the sub-scales of these two measures correlated highly, *r* = 0.63, but low enough to suggest divergence, (c) both differentially predict membership in a Facebook group, and (d) both are not isomorphic with neuroticism. We concluded that HSPS primarily measures the *emotional* reaction to sensory stimulation, whereas ASP the *behavioral* reactions. We offer shorter yet reliable measures for both theories.

## Introduction

All living organisms respond to variations in their physical environment through sensors that detect changes in energy patterns (such as light or sound; Strelau, 1994). The likelihood of detection of changes in the environment depends on individual differences. Such individual differences are the subject of two theories—Sensory-Processing Sensitivity (SPS; Aron and Aron, 1997) and the Sensory processing theory (SP; Dunn, 1997). However, researchers of these theories work in relatively independent research areas, each with its conceptualization of the dimensions of these individual differences and measurement instruments. SPS research suggests three interrelated sub-constructs, and the SP theoretically offers four individual difference profiles. Thus, our first goal is to test the presumed dimensions of SPS and SP, and our second goal is to test whether the measures emanating from these theories converge or diverge. Next, it is unclear whether each of the measures associated with these theories diverges from neuroticism and other related traits. Thus, our third goal is to establish divergent validity for the constructs of each theory. Finally, the SPS contains 27 items and the SP 60. Therefore, our last goal was to offer shorter versions for these scales to facilitate research covering the entire spectrum of phenomena described by the two theories. In summary, we investigated the measures of SPS and SP. Specifically, we (a) tested their dimensionality, (b) explored their similarities and uniqueness, (c) tested their divergent validity from neuroticism and other constructs, and (d) offered shorter and reliable versions of these scales.

### Sensory-processing-sensitivity theory

The SPS theory, emanating from social and personality psychology, suggests individual differences in the capacity to process stimuli. People *high* in SPS (highly sensitive persons, or HSPs) notice subtleties and nuances in the environment that are not noticed by people low in SPS (Strelau, 1987; Aron and Aron, 1997; Belsky and Pluess, 2009; Aron, 2010; Aron et al., 2012). On the other hand, HSPs can handle a smaller amount of stimulation than others (Strelau, 1987; Aron and Aron, 1997; Belsky and Pluess, 2009). Therefore, HSPs are more likely to experience overarousal (Pavlov, 1927/1960) and to be overwhelmed (Aron and Aron, 1997; Belsky and Pluess, 2009). When HSPs are overwhelmed, they struggle to process the stimuli. Consequently, they experience intensive-physical reactions (such as rapid heart rate), which increases their overarousal. Their overarousal is increased until they attempt to block the stimulation—reach into transmarginal inhibition. The potential overarousal of HSPs may cause them to be more careful before responding to the environment (Aron and Aron, 1997, 2010; Aron, 2000; Jagiellowicz et al., 2010).

The standard measures of SPS in human adults are the 27-item HSP scale (HSPS; Aron and Aron, 1997) and the short version of the 12-item HSPS questionnaire (Pluess, 2015; Pluess and Boniwell, 2015). Initially, the 27-items HSPS questionnaire was considered to reflect a single factor (Aron and Aron, 1997). However, further studies suggested three factors (Smolewska et al., 2006; Aron and Aron, 2010; Booth et al., 2015; Grimen and Diseth, 2016): (a) *Ease of Excitation* (EOE)—becoming overwhelmed mentally by external and internal demands; (b) *Aesthetic Sensitivity* (AES)—aesthetic awareness; and (c) *Low Sensory Threshold* (LST)—unpleasant sensory arousal due to external stimuli. We sought to re-test the factor structure of the HSPS on a different, and larger, sample.

### The sensory processing theory: Patterns of sensory processing

Another theory that pertains to a similar sensory pattern is Dunn’s sensory processing theory, emanating from occupational therapy (SP; Dunn, 1997, 2014). The SP theory, like the SPS theory, suggests individual differences in sensory processing but attributes these differences to high vs. low neurological thresholds and active vs. passive behavioral responses. Unlike the SPS theory, which focuses only on people *high* in sensory processing sensitivity, the SP theory considers the threshold of sensory processing and the behavioral response to sensations as separate continua (Dean, 2015): neurological thresholds for stimulation (high-low) and behavioral response (active-passive). These two axes underlie four sensory processing styles (Dunn, 1997, 2001): (a) *Sensory Sensitivity (Ss)*—a low neurological threshold that causes a strong physiological response but a passive-behavioral response with slow habituation to the sensation; (b) *Sensory Avoiding (SA)*—a low neurological threshold that causes a strong physiological response, and an active-behavioral response to withdraw from the sensation (therefore quick habituation); (c) *Low Registration (LR)*—a high neurological threshold that causes a weak physiological-response and a passive-behavioral response with delayed habituation to the sensation; and (d) *Sensation Seeking (Se)*—a high neurological threshold that causes a weak physiological response, but an active-behavioral response that pursues sensation (therefore slow habituation).

The standard measure of SP in children is the 86-item Sensory Profile 2 (Dunn, 2014), and in adults is the 60-item Adolescent Adult Sensory Profile scale (ASP; Brown and Dunn, 2002). A factor analysis of the ASP yielded 16 factors. Brown et al. (2001) used a scree test and suggested that the first four factors are similar to the theory. As predicted, it indicated factors of *Low Registration* and *Sensation Seeking*, but inconsistent with the theory, *Sensory Sensitivity,* and *Sensory Avoiding* items loaded together on two additional factors (Brown et al., 2001, p. 78).

### Sensory-processing sensitivity and sensory processing theory: Same or different?

The SPS and the SP theories lay on the same theoretical ground, suggesting that some people are more sensitive than others to sensory information. Therefore, some researchers refer to these two theories as the same (Jerome and Liss, 2005; Bakker and Moulding, 2012; Meredith et al., 2016) or consider the SPS the same as the low neurological threshold profiles of the SP; *Sensory Sensitivity* (Benham, 2006; Minshew and Hobson, 2008; Brown and Stoffel, 2010) or *Sensory Avoiding* (Ben-Avi et al., 2012).

Other researchers suggest that these theories should be distinguished (Aron, 2011; Levit-Binnun et al., 2014). Their main argument is that while SP refers only to sensitivity to sensory stimulation (Levit-Binnun et al., 2014), SPS refers to a deeper depth of processing, involving higher emotional arousal and empathic abilities (Aron and Aron, 1997; Aron, 2013; Levit-Binnun et al., 2014). The high emotional arousal and empathic abilities cause aesthetic awareness of the environment’s subtleties and nuances, such as delicate or fine scents, tastes, sounds, art, and music. Indeed, aesthetic awareness is one of the SPS factors labeled *Aesthetic Sensitivity* (Smolewska et al., 2006). Aesthetic sensitivity is not considered in the SP theory.

The debate regarding the convergence, or divergence, of the constructs of SPS and SP remained primarily theoretical. One exception is a study that reported that SPS was correlated with *Low Registration*, *Sensory Sensitivity,* and *Sensory Avoiding*, *rs* = 0.21, 0.50, 0.48, respectively, where *Sensory Seeking* was not correlated with SPS (Meredith et al., 2016; *N* = 116). These correlations are far from unity and may suggest divergence.

Moreover, both the SP and SPS theories suggest that although their respective constructs are related to neuroticism, they contain variances not shared with neuroticism (Aron and Aron, 1997; Dunn, 2001). That is, the theories claim that their construct diverges from neuroticism. Therefore, we tested whether we could establish the divergent validity of SP and SPS with empirical data (Lawson and Robins, 2021). We sought to determine whether neuroticism is only a *sibling* construct to SPS and SP or their identical twin (Lawson and Robins, 2021). We probed this question (a) theoretically, (b) with meta-analyses, and (c) through data we collected.

Moreover, we expanded the probing of the divergent validity of SPS and SP to other candidate constructs whose measures may overlap with SPS and SP. First, we considered measures of all the Big Five traits rather than only neuroticism. Second, we considered specific constructs that may threaten the divergent validity of SPS and SP: trait anxiety, trait social anxiety, shyness, introversion (as part of the Big Five), attention deficit hyperactivity disorder (ADHD), trait mindfulness, family environment, and attachment style. For example, we asked whether SP merely measures trait social anxiety. Suppose we establish that the correlations of neuroticism and all the other constructs with SPS and SP are low enough. In that case, we will establish the divergent validity of SPS and SP, providing evidence that they should be considered unique traits. Therefore, next, we review why neuroticism and the other constructs could overlap with SPS and SP.

### Neuroticism

Neuroticism is defined as maladjustment or negative emotionality (Costa and McCrae, 1985). Neuroticism is also called personality-negative affectivity or negative emotionality (Eysenck and Eysenck, 1975; Watson and Clark, 1984). Neuroticism is a temperamental sensitivity to negative stimuli (Zobel et al., 2004) and an inclination to experience psychological distress (Ormel and Wohlfarth, 1991). It predisposes them to experience negative emotions, such as suffering more acutely from misfortunes (McCrae, 1990). Moreover, neuroticism refers to a tendency towards experiencing tension, anxiety, depression, hostility, irrational thinking, impulsivity, self-pity, self-consciousness, low self-esteem (Penley and Tomaka, 2002), having unrealistic ideas, an inability to control urges, and inefficient ways of coping with stress (Costa and McCrae, 1985).

The authors of both SP and SPS theories discussed the relationship of the respective constructs with neuroticism. Dunn (2001) argued that neuroticism relates to the *Sensory Avoiding* profile, which refers to a low neurological threshold and an active behavioral response to stimuli. However, while *Sensory Avoiding* refers to a low neurological threshold for all stimuli, neuroticism definition refers only to sensitivity to *negative* stimuli. We did not find any study testing this argument.

Aron and Aron (1997) argued that SPS is often, but mistakenly, considered as nothing more than neuroticism (e.g., Howarth, 1986). On the one hand, SPS was correlated to neurotic personality traits, such as stress, anxiety, and depression (Smolewska et al., 2006; Ahadi and Basharpoor, 2010). Also, neuroticism yielded positive correlations with SPS, and its component, especially with the SPS components of *Low Sensory Threshold* and *Ease of Excitation* (Aron and Aron, 1997; Andresen et al., 2005, 2017; Smolewska et al., 2006; Ahadi and Basharpoor, 2010; Aron et al., 2010; Jagiellowicz et al., 2010; Acevedo et al., 2014; Sobocko and Zelenski, 2015; Listou Grimen and Diseth, 2016; Mullet et al., 2016; Lionetti et al., 2018;Pluess et al., 2018; Weyn et al., 2019). Nevertheless, *Low Sensory Threshold* and *Ease of Excitation* could be associated with negative emotions and experiences merely because their measures include many negatively worded items, describing negative consequences of greater depth of information processing (Greven et al., 2019).

On the other hand, SPS theory is compatible with other theories postulating individual differences in reacting both to negative and positive environments, such as differential susceptibility (Belsky, 1997; Belsky and Pluess, 2009) and biological sensitivity to context (Boyce and Ellis, 2005). Thus, SPS and other environmental sensitivity theories refer to a broader line of sensitivity to all kinds of stimuli, whereas theories about neuroticism refer specifically to sensitivity to negative stimuli. Therefore, the SPS-neuroticism correlation might be caused by the tendency of both HSPs and neurotic individuals to respond to stimuli cautiously (Smolewska et al., 2006). However, SPS is also theorized to reflect sensitivity to positive stimuli, unlike neuroticism, which refers only to sensitivity to negative stimuli (Zobel et al., 2004). Consistent with the theories, brain research found that SPS is correlated with stronger reactions to positive images than to negative images (Pluess and Belsky, 2013; Acevedo et al., 2014), while neuroticism is associated with greater sustained *medial prefrontal cortex* for sad facial expressions, but not for happy or fearful facial expressions (Haas et al., 2008).

To address whether SPS is nothing more than neuroticism, we meta-analyzed all the relevant correlations with a random model (Borenstein et al., 2021). However, several correlations were reported by the same author. To account for the nesting of some correlations within an author, we used a three-level meta-analysis (Van den Noortgate et al., 2013) available in the *metafor* package (Viechtbauer, 2010) in R (R Core Team, 2018). The mean-weighted correlation of SPS with neuroticism was $\bar{r}$ = 0.47, *k* = 21, *N* = 8,494 (see Supplementary Figure S1 in Section A). Our results are consistent with a meta-analysis conducted by Lionetti et al. (2019), $\bar{r}$ = 0.40, *k* = 8, *N* = 6,790. In addition, a subsample of the studies in this meta-analysis, *k* = 11, *N* = 6,519, also reported correlations with SPS subscales and indicated that neuroticism correlates with *Low Sensory Threshold*, $\bar{r}$ = 0.30, *Ease of Excitation*, $\bar{r}$ =0.52, but barely with *Aesthetic Sensitivity*, $\bar{r}$ =0.12 (see Supplementary Figures S2, S3, and S4 in Section A). None of the analyses suggested that SPS and neuroticism are isomorphic. We sought to replicate these findings and test the correlations of SP components with neuroticism in data we collected to better understand the differences and similarities among SPS and ASP items.

### Trait anxiety and trait social anxiety

HSPs were hypothesized to be at risk for adverse emotional and psychological outcomes (Aron and Aron, 1997). Indeed, SPS is correlated with anxiety (Liss et al., 2005, 2008; Bakker and Moulding, 2012; Brindle et al., 2015), social anxiety (Liss et al., 2005; Hofmann and Bitran, 2007; Liss et al., 2008; Bakker and Moulding, 2012), limitations in communication (Liss et al., 2008), and social phobia (Neal et al., 2002). Thus, we anticipate that trait anxiety and trait-social anxiety will also be positively correlated with subscales of SPS and SP related to sensitivity to negative stimuli (SPS: *Ease of Excitation, Low Sensory Threshold*, SP: *Sensory Sensitivity, Sensory Avoiding, Low Registration*). Nevertheless, given that sensory sensitivity applies to all kinds of stimuli, rather than mostly-anxious stimuli, we expect to find divergence between these concepts.

### Shyness and introversion

The over-arousal characterizing HSPs’ reaction to stimuli was theorized to lead to inhibition and social withdrawal (Aron and Aron, 1997; Aron et al., 2005). Social withdrawal and inhibition are social strategies mostly related to introversion (Eysenck, 1957; Eysenck, 1981) and shyness (Asendorpf, 1990; Kagan, 2001). Therefore, Aron and Aron (1997) argued that HSPs might be perceived as introverts or shy. Note that one of the HSPS scales contains an item about shyness: “When you were a child did parents or teachers seem to see you as sensitive or shy?.” Not surprisingly, Aron et al. (2005) found positive correlations between shyness and SPS; *r* = 0.20, *p* < 0.001; *r* = 0.26, *p* < 0.001.

We meta-analyzed the relevant correlations we could find regarding introversion and SPS with a random model (Jerome and Liss, 2005; Levit-Binnun et al., 2014; Meredith et al., 2016) and found a positive correlation; $\bar{r}$ = 0.23, *k* = 21, *N* = 7,989 (see Supplementary Figure S5 in Section B). This result suggests that introversion is different from SPS. Therefore, we sought to replicate these findings and test the correlations of SPS and SP components with introversion and shyness.

### ADHD

Both ADHD and SPS are often associated with unusual responses to sensory stimulations and include elements of emotional sensitivity, over-reactivity, and experiencing others’ emotions as heightened (Aron and Aron, 1997; Maté, 2000; Friedman et al., 2003; Jensen and Rosen, 2004; Robbins, 2005; Aron, 2010; Aron et al., 2012; Acevedo et al., 2014; Sanz-Cervera et al., 2017; Little et al., 2018). Moreover, SPS may also be related to inattention, a main symptom of ADHD, due to HSPs’ tendency to experience over-arousal, pay attention to subtleties and nuances and be reactive to emotional stimuli (Aron et al., 2012; Jagiellowicz et al., 2016).

On the other hand, ADHD also presents elements diverging from SPS, such as deficits in response inhibition (Crosbie et al., 2013; Polner et al., 2015), hyperactivity, and impulsivity (Stormont, 1998, 2001; Uekermann et al., 2010; Chamberlain et al., 2017), seeking self-stimulation (Gaub and Carlson, 1997; Stormont, 1998, 2001; Hodgens et al., 2000; Wheeler Maedgen and Carlson, 2000; Robbins, 2005; Solden, 2012), reduced empathy (Uekermann et al., 2010), and reduced ability to recognize emotions (Jensen and Rosen, 2004).

Panagiotidi et al. (2020) found a positive correlation between ADHD and SPS; *r* (274) = 0.42, *p* < 0.001, and revealed, by exploratory factor analysis, two factors of ADHD, while one of them also contains all items of HSPS, suggesting that ADHD symptomatology may include elements of SPS, without this implying unity. We sought to replicate this finding and test the correlations of SPS and SP components with ADHD.

### Trait mindfulness

Both SPS and mindfulness are related to sensitivity to environmental and internal sensations. However, the SPS’s sensitivity to subtle stimuli is coupled with an unpleasant experience of overstimulation (Aron and Aron, 1997). In contrast, in mindfulness, the awareness of subtle stimuli is purposeful and nonjudgmental (Kabat-Zinn, 2003). Thus, it is not surprising that, unlike SPS, mindfulness correlates negatively with trait anxiety (Miller et al., 1995; Weinstein et al., 2009; Barnhofer et al., 2011). Moreover, SPS was negatively correlated with mindful attention, awareness, and acceptance (Bakker and Moulding, 2012). Consequently, we predict that SPS would also correlate negatively with trait mindfulness. Yet, only the subscales pertaining to negative experiences should show this pattern.

### Family environment and attachment style

Aron et al. (2005, 2010) argued that HSPs are prone to negative emotional outcomes, such as anxiety, only if they experienced a poor-family environment. They found that HSPs who recalled a troubled childhood were more introverted and emotional (Aron and Aron, 1997). Nevertheless, Meyer and Carver (2000) failed to find a significant interaction between negative childhood memories and SPS in predicting features of avoidant personality disorder. Moreover, Liss et al. (2005) found that SPS is an independent risk factor for experiencing psychological distress (depression and anxiety) above and beyond parental experiences. But still, an interaction was found between SPS and parental care when measuring depression (Liss et al., 2005). Although this last paper does not fully support Aron et al. (2005, 2010), it still suggests that HSPs may be more sensitive to poor parenting than people low in SPS. Therefore, we explore whether the Perceived Social-Support of the entire family (PSS-Fa; Procidano and Heller, 1983), specifically in childhood (up to the age of 18), is associated with SPS and whether it moderates the association of SPS with neuroticism.

An extension of the view that SPS is linked to childhood experiences can also be assessed with attachment styles (Jerome and Liss, 2005; Levit-Binnun et al., 2014; Meredith et al., 2016). We meta-analyzed the correlations found in these papers with a random model. The mean-weighted correlation of *Sensory Sensitivity* with *Attachment Anxiety* was $\bar{r}$ = 0.28, *k* = 3, *N* = 443, and with *Attachment Avoidance* was $\bar{r}$ = 0.18, *k* = 2, *N* = 310 (Levit-Binnun et al., 2014; Meredith et al., 2016). *Sensory Avoidance* was related to *Attachment Anxiety;* $\bar{r}$ = 0.29, *k* = 2, *N* = 310, and *Attachment Avoidance;* $\bar{r}$ = 0.21, *k* = 3, *N* = 443. *Low Registration* was related to *Attachment Anxiety;* $\bar{r}$ = 0.23, *k* = 3, *N* = 443. Moreover, *Sensory Seeking* is generally reported as related to *Secure attachment* (Jerome and Liss, 2005; Levit-Binnun et al., 2014; Meredith et al., 2016). Indeed, Levit-Binnun et al. (2014; *N* = 194) reported significant negative correlations of *Sensory Seeking* with both *Attachment anxiety* (*r* = −0.13, *p* > 0.05) and *Attachment Avoidance* (*r* = −0.24, *p* < 0.001). Regarding SPS, it was reported to have a significant positive correlation with *Attachment Anxiety* (Meredith et al., 2016; N = 116, r = 0.23, *p* < 0.05), and with an attachment subscale of *Feeling Upset and Misunderstood by Parents* (Meyer et al., 2005; N = 156, r = 0.30 *p* < 0.01). Therefore, we sought to determine whether attachment styles are correlated with sensory sensitivity and moderate the association between SPS and neuroticism.

### Overview

We set to address four questions: (a) What are the dimensions underlying the measures of SPS and SP? (b) To what degree do these measures and their dimensions converge? (c) Can divergent validity of SPS and SP be established when correlated with neuroticism and other candidate constructs? (d) Is it possible to offer a short and reliable version for measuring SPS and SP? To test our questions, we collected two samples. In the second sample, we measured the HSPS, ASP, Big 5, Shyness, ADHD, trait anxiety, trait social anxiety, trait mindfulness, family environment, and attachment style. We obtained ethical approval from our University’s Ethics Committee for collecting both samples and obtained informed consent from all participants. In one sample, we measured the HSPS, ASP, three items of neuroticism, and membership in the HSPs Facebook group. 

Next, we combined both samples and explored the dimensionality of their items with *exploratory graph analysis* (EGA; Golino and Epskamp, 2017; Golino et al., 2020b). Based on the EGA, we removed items showing poor item stability and constructed new sub-scales reflecting various aspects of sensory sensitivity based on the stable items. Armed with these new scales, we tested the divergent validity of SPS and SP from neuroticism and other scales.

## Materials and methods

### Participants and procedure

**Figure 1:**
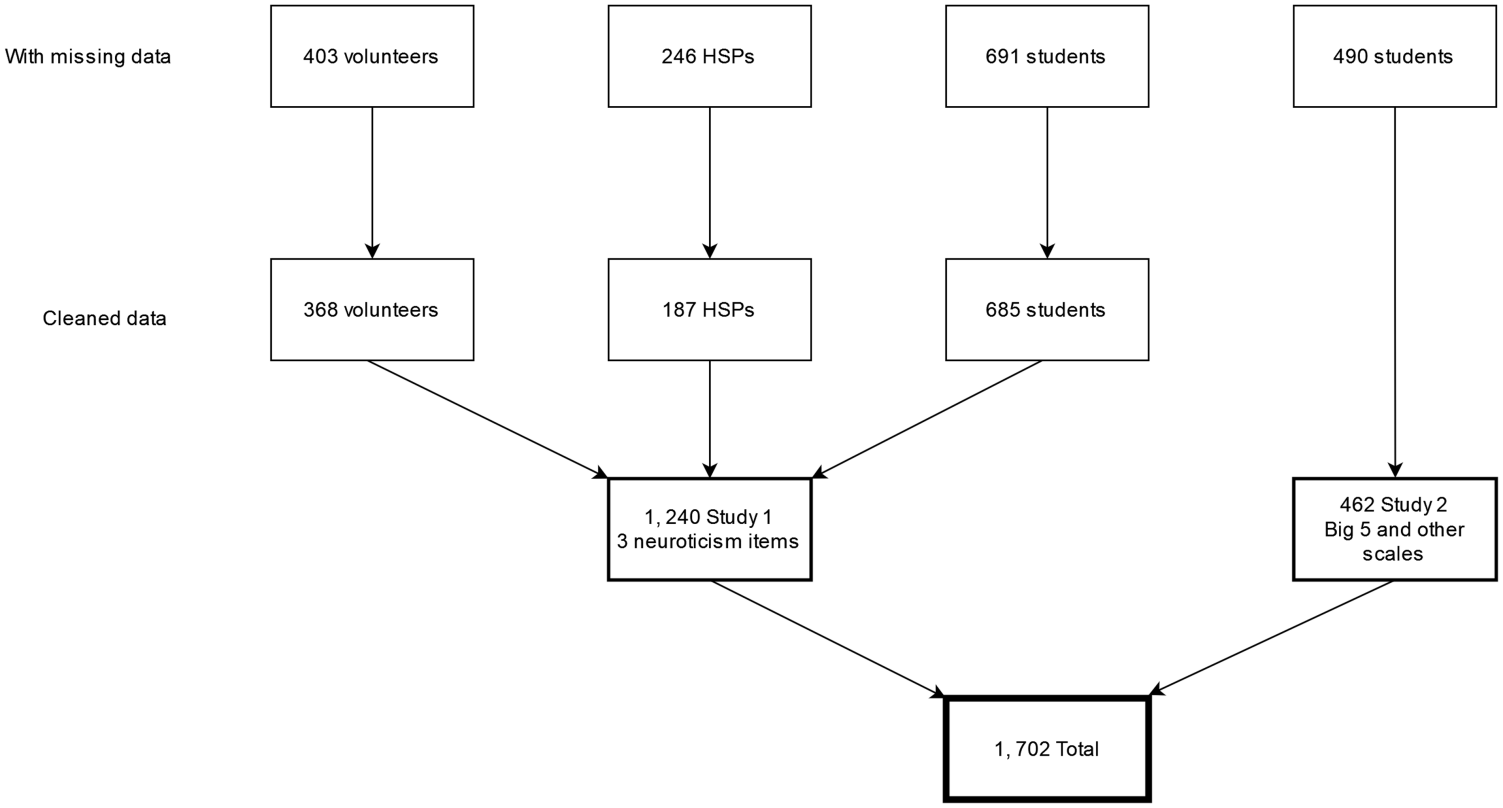
Flowchart of number of participants.

We collected two samples of Israeli participants that were asked to participate in a study designed to learn about highly sensitive people. We provided all the participants with a link to a Qualtrics survey and asked them to fill out the HSPS, the ASP measure, the neuroticism measure, and a few demographic questions (see Figure 1). In the first sample, we recruited 1,340 students and volunteers. In the second sample, we recruited 490 first-year undergraduate students to participate in a laboratory experiment. Students participated in exchange for course credit. The experiment is not completed yet, and we extracted personality data collected before participating in the experiment. Of these, 128 (7%) had three or more missing items in any of the HSPS, ASP, or Neuroticism items. We deleted these participants’ data and imputed missing data on HSPS and ASP, with mean substitution, for those with one (138) or two missing items (34).

The respondents in the combined sample, *N* = 1,702, include 1,147 first-year undergraduate students and 555 volunteers, *M*_age_ = 26.9 (66.7% female). The students participated in the study in exchange for course credit. The volunteers were recruited with snowball sampling *via* Facebook. A request to volunteer for this study was shared on several Facebook Groups (with the consent of the groups’ managers). Importantly, one Facebook Group that yielded 187 responses was a group of Highly Sensitive People in Israel.^1^

### Measures

We translated all the questionnaires to Hebrew and had a graduate student back-translated the items to English. Based on the back translation, the authors resolved minor discrepancies. To increase the validity of all scales, we presented all items on 11-point scales ranging from 0 = *Not true at all* to 10 = *Very true*, as more points on the scale enhance validity (Aguinis et al., 2009). Using 11-point scales has the drawback of not allowing comparison of the means in our study to the means of the measures reported by others. However, 11-point scales can increase the observed correlations among all items and reveal stronger correlations between SPS, SP, and all the constructs threatening their divergent validity. Thus, we used 11-point scales to provide the most rigorous divergent-validity tests.

#### Highly sensitive person

We used the 27-items HSP Scale (HSPS; Aron and Aron, 1997). The HSPS is reliable and widely used in HSPs studies (Aron and Aron, 1997; Meyer et al., 2005; Benham, 2006; Hofmann and Bitran, 2007; Jagiellowicz et al., 2010; Aron et al., 2012; Acevedo et al., 2014).

#### Adolescent adult sensory profile

We used the 60-item ASP scale (Brown and Dunn, 2002), based on Dunn (1997) Model of Sensory Processing. The ASP scale includes 15 items for each of the four sensory profiles and is widely used in adult sensory studies (Brown et al., 2001; Brown and Dunn, 2002; Pohl et al., 2003; Chung, 2006; Engel-Yeger, 2012; Üçgül et al., 2017).

#### Neuroticism

In Sample 1, we used a 3-item neuroticism scale by Aron and Aron (1997), as was done in some SPS studies (Aron and Aron, 1997; Aron et al., 2005, 2010; Andresen et al., 2017). The items were “Are you a tense or worried person by nature?,” “Are you prone to fears?” and “Are you prone to depression?.” In Sample 2, we used the Big Five (see below).

#### Trait anxiety

We used 20 items regarding trait anxiety from the State–Trait Anxiety Inventory (STAI; Spielberger, 1983), widely used for assessing trait anxiety (Sesti, 2000).

#### Trait-social anxiety

We used the 20-item Social Interaction Anxiety Scale (SIAS; Mattick and Clarke, 1998). SIAS is a reliable measure of adults’ trait-social anxiety (Mattick and Clarke, 1998; Osman et al., 1998; Fergus et al., 2012; Le Blanc et al., 2014).

#### Shyness

We used the 20-item Shyness Scale (Cheek and Melchior, 1985), a validated measure of shyness (Melchior and Cheek, 1990; Cheek and Krasnoperova, 1999).

#### Big-five

We used the 44-item Big-Five Inventory (BFI; John and Srivastava, 1999). The BFI contains five scales for the five-trait model: Neuroticism, Extraversion, Openness, Agreeableness, and Conscientiousness. BFI scales include 8–10 items each and are reported to have high internal consistency reliabilities (John et al., 1991, 2008).

#### ADHD

We used the 20-item Barkely Deficit in Executive Function Scale–Short Form (BDEFS-SF; Barkely, 2011) and the 6-item Adult ADHD Self-Report Screening Scale for DSM-5 (ASRS-5; Ustun et al., 2017). Previous studies demonstrated excellent internal consistency for BDEFS-SF (Barkley, 2011; Flannery et al., 2017; Lace et al., 2020) and excellent psychometric properties for DSM-5’s Self-Report Screening Scale (sensitivity, 91.4%; specificity, 96.0%; AUC, 0.94; Somma et al., 2021). We used these instruments because ADHD is often under-identified in the adult population (Wender et al., 2001; Biederman et al., 2004).

#### Trait mindfulness

We used the 15-item Mindful Attention Awareness Scale (MAAS; Brown and Ryan, 2003) to assess dispositional mindfulness. MAAS has been validated for use with adults, and its internal consistency levels were found to be acceptable (Brown and Ryan, 2003; Carlson and Brown, 2005; MacKillop and Anderson, 2007; McCracken and Zhao-O’Brien, 2010; Catak, 2012; Deng et al., 2012).

#### Family-support

We used the Perceived Social Support–Family Scale (PSS-Fa; Procidano and Heller, 1983). Participants were asked to answer this measurement regarding their childhood (up to the age of 18).

#### Attachment styles

We used A Brief Version of the Experiences in Close Relationships Scale (ECR-12; Lafontaine et al., 2016).

### Analysis

Typically, researchers use exploratory factor analysis, or multi-dimensional scaling, to uncover the factorial structure of items of questionnaires. However, these techniques are less accurate and informative than the relatively new exploratory graph analysis (EGA; Golino and Epskamp, 2017; Golino et al., 2020b).^2^ First, EGA is more likely to uncover the correct number of factors underlying the data. In EGA, the factors are called communities. Second, EGA estimates its solution’s stability concerning the number of communities and the allocation of each item into a community. This feature allows the identification of unstable items that could be discarded from final analyses (conceptually similar to items loaded on multiple factors). Discarding unstable items makes the differences between the subscales stronger, but more accurate, and may provide shorter measurements. This is suitable for both ASP and HSPS because previous studies of these measurements reflect more than a single factor. Last, EGA provides a graphical output showing the degree of (dis)similarity between the communities and the items within them.

Technically, we bootstrapped our results over 500 samples using the *bootEGA* function in the *EGAnet* package in R (v.0.9.5; Golino et al., 2020a; Christensen and Golino, 2021). We used EGA to uncover the data structure *via* a network graph without assuming a simple structure (exploratory factor analysis seeks a solution with a simple structure). This approach is suited to test empirical dimensions in multi-dimensional data, and thus can test SP theory of two underlying dimensions, and may help solve the debate regarding the convergence, or divergence, of the constructs of SPS and SP. Based on EGA results, we constructed sub-scales of HSPS and ASP, computed their correlations with each other and the measured traits, and used them all to predict belonging to the HSP-Facebook group, using logistic regression.

The EGA bootstrapping provides information about the likely true number of communities (factors) underlying the data and the stability of the items (the percent of the time that a given item appears in the same community across the samples). The best estimate of the number of communities is the median across bootstrapped samples, as used here. As per item stability, it is recommended to retain items with stability between 0.65 and 0.75 (Christensen and Golino, 2021). Our first EGA yielded many items with lower stability. Therefore, we discarded unstable items with three iterations. First, we ran EGA on all items and discarded those with stability below 0.55; next, we re-ran EGA on the surviving items and discarded those with stability below 0.65; and, finally, we re-ran EGA once more without items with stability below 0.75.

## Results

**Figure 2:**
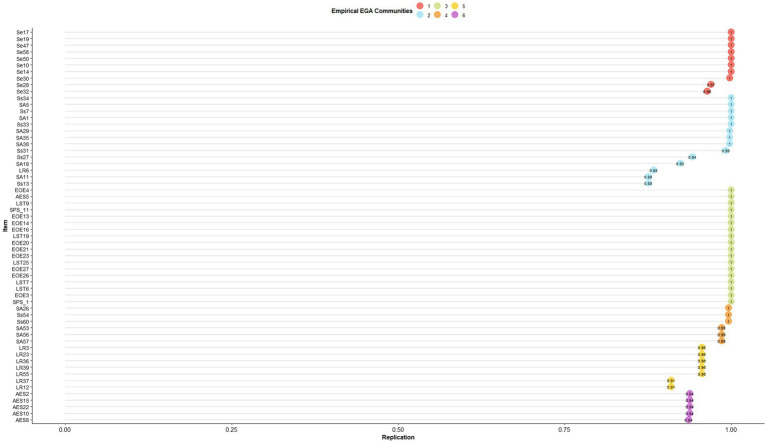
Structural consistency of the six community solution of highly Sensitive person scale (HSPS) and the adolescent adult sensory profile (ASP) items. *N* = 1,702. SPS, items that previous studies did not assign to any subscale of the Highly sensitive person scale; EOE, ease of excitation; AES, aesthetic sensitivity; LST, low sensory threshold; SS, sensory sensitivity; SA, sensory avoiding; Se, sensation seeking; LR, low registration.

**Figure 3:**
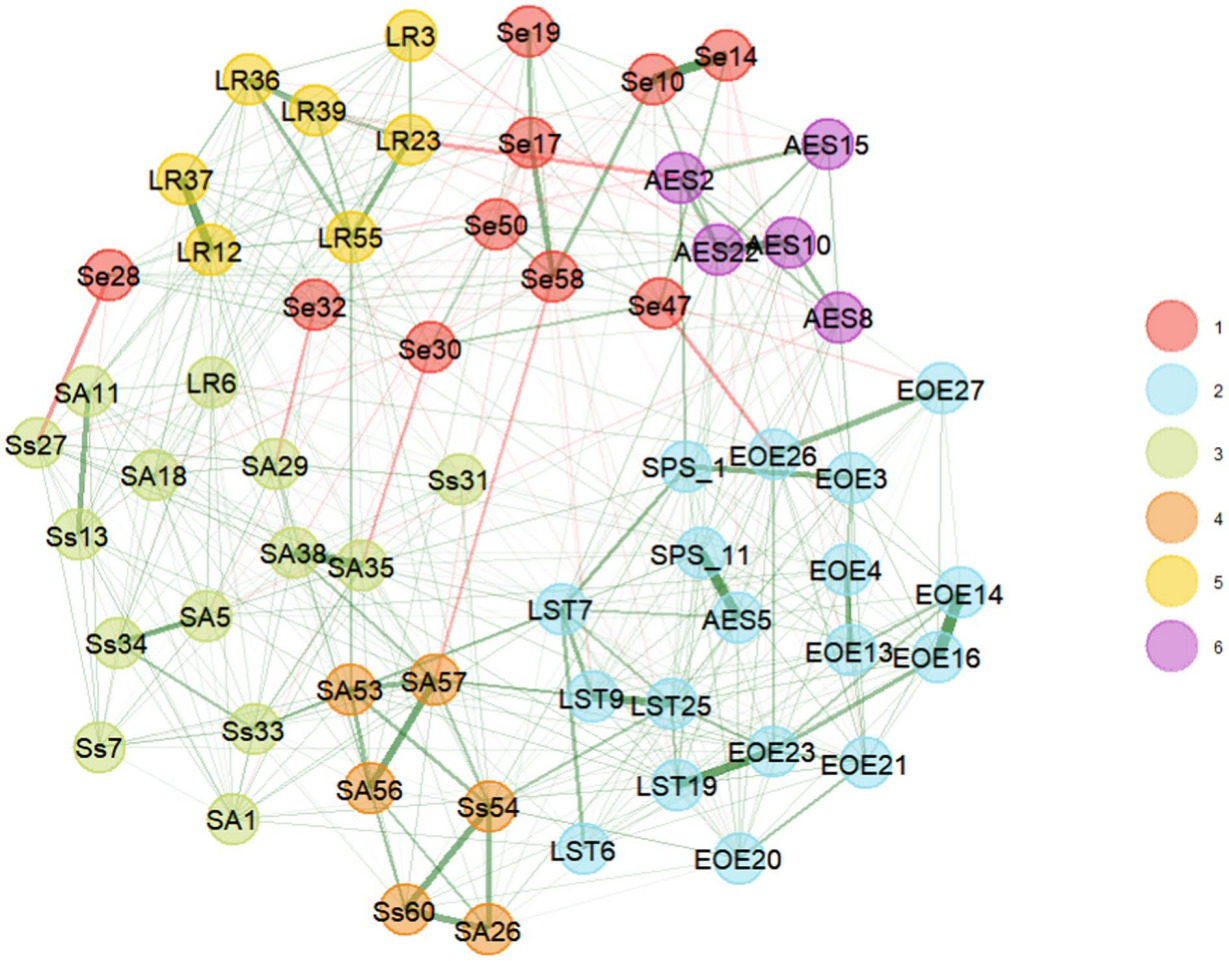
Network plot of the six community solution of highly sensitive person scale (HSPS) and the adolescent adult sensory profile (ASP) items. *N* = 1,702. EOE, Ease of excitation; AES, aesthetic sensitivity; LST, low sensory threshold; Ss, sensory sensitivity; SA, sensory avoiding; Se, Sensation seeking; LR, low registration.

To facilitate the interpretation of our results and comparison to past findings, we labeled each of the 60 ASP items based on their original classification into four profiles (Ss*,* SA, LR, and Se), and the 27 HSPS items based on the prior factor analyses (EOE, AES, and LST). We subjected these items to EGA with 500 parametric bootstraps and plotted the median solution, containing only the stable items that belonged to only one community (see Table 1). In the first run, the bootstrapping indicated that four to eight communities might underlie the data with a median of six (40.2% of the samples). It also indicated that 22 of the 87 items had stability below 0.55. After dropping these items, the bootstrapping indicated that four to seven communities might underlie the data with a median of six (94% of the samples). Still, two items had stability below 0.65. We dropped these two items. Without these, the bootstrapping indicated the same number of communities with a median of six (92% of the samples). Still, three items had stability below 0.75. After dropping these three items, the conclusion did not change with a median of six communities (89% of the samples; median CI [5.32, 6.68]). All the remaining 60 items have good stability (see Figure 2). Figure 3 shows the network of the SPS and SP items and their underlying six communities: two containing HSPS items and four ASP items. Thus, the EGA differentiates clearly between HSPS and ASP.

**Table 1:** EGA loadings table of the six HSPS and ASP sub-scales (without the unstable items).

*Item*	*X1*	*X2*	*X3*	*X4*	*X5*	*X6*
Se17	**0.19**	−0.01	−0.01	−0.01	0.02	−0.00
Se19	**0.18**	−0.01	0.00	−0.00	0.00	0.02
Se58	**0.26**	−0.02	−0.01	−0.04	0.00	0.04
Se47	**0.17**	0.00	−0.05	0.00	0.01	0.02
Se50	**0.16**	0.00	0.00	0.02	0.04	0.05
Se10	**0.26**	−0.01	0.00	0.01	0.00	0.09
Se14	**0.18**	−0.01	−0.02	0.00	−0.00	0.00
Se30	**0.18**	−0.04	0.00	0.00	0.03	0.01
Se28	**0.11**	−0.05	0.00	0.00	0.00	0.01
Se32	**0.11**	0.01	−0.00	−0.00	0.06	0.01
SA1	−0.01	**0.12**	0.03	0.04	0.01	0.00
Ss33	0.01	**0.14**	0.04	0.06	0.01	0.00
SA35	−0.03	**0.21**	0.04	0.04	0.01	0.01
SA38	−0.03	**0.20**	0.03	0.06	0.03	0.00
Ss7	−0.00	**0.11**	0.01	0.02	0.01	−0.00
SA5	−0.02	**0.17**	0.02	0.00	0.01	−0.02
Ss34	0.00	**0.19**	0.00	0.00	0.03	−0.00
SA29	−0.00	**0.14**	0.01	0.04	0.01	−0.00
Ss31	0.01	**0.07**	0.02	0.03	0.01	0.00
Ss27	−0.07	**0.13**	0.00	0.01	0.01	−0.00
SA18	−0.02	**0.12**	0.01	0.04	0.07	−0.00
LR6	0.00	**0.13**	0.02	0.01	0.09	−0.00
Ss13	−0.01	**0.12**	0.00	0.04	0.07	−0.00
SA11	−0.00	**0.15**	0.01	0.01	0.08	−0.00
EOE4	−0.00	0.03	**0.15**	0.00	−0.00	0.01
AES5	−0.00	0.01	**0.21**	0.00	0.00	0.01
SPS_11	0.00	0.03	**0.19**	0.01	0.02	0.07
EOE13	−0.00	0.01	**0.20**	0.01	0.01	0.01
EOE14	−0.00	0.00	**0.22**	0.02	0.00	0.00
EOE16	−0.00	0.00	**0.26**	0.01	0.00	0.00
EOE20	0.01	0.01	**0.13**	0.05	0.00	0.01
EOE21	−0.01	0.01	**0.19**	0.00	0.00	0.02
EOE23	−0.01	0.02	**0.26**	0.04	0.01	0.00
LST25	−0.00	0.02	**0.25**	0.06	−0.00	0.02
EOE27	−0.02	0.00	**0.10**	0.00	0.00	0.01
LST9	−0.01	0.00	**0.17**	0.06	0.00	0.03
EOE26	−0.05	0.04	**0.17**	0.01	0.02	0.00
LST19	−0.01	0.04	**0.19**	0.05	0.01	0.03
LST7	0.00	0.07	**0.20**	0.01	0.00	0.07
LST6	0.00	0.02	**0.09**	0.01	0.01	0.01
EOE3	0.00	0.00	**0.19**	0.02	0.00	0.08
SPS_1	0.01	0.00	**0.15**	0.04	0.00	0.08
SA26	0.01	0.01	0.03	**0.21**	0.00	0.01
Ss54	0.00	0.02	0.09	**0.28**	0.03	0.00
Ss60	−0.00	0.01	0.03	**0.27**	0.01	0.00
SA56	0.01	0.05	0.00	**0.27**	0.01	0.00
SA53	−0.00	0.08	0.02	**0.22**	0.04	0.00
SA57	−0.05	0.12	0.06	**0.25**	0.01	0.00
LR3	0.00	0.04	−0.00	0.00	**0.15**	−0.05
LR23	0.01	0.05	0.00	0.00	**0.24**	−0.07
LR36	0.03	0.04	0.00	0.00	**0.25**	−0.01
LR39	0.03	0.05	−0.01	−0.00	**0.22**	−0.04
LR55	0.02	0.03	0.00	0.07	**0.26**	−0.03
LR12	0.03	0.08	0.02	0.02	**0.19**	−0.00
LR37	0.04	0.05	0.01	0.01	**0.21**	0.00
AES2	0.01	0.00	0.04	−0.00	−0.08	**0.20**
AES15	0.02	−0.00	0.02	0.00	−0.02	**0.20**
AES22	0.07	0.00	0.06	0.00	−0.03	**0.27**
AES10	0.11	−0.00	0.05	0.00	−0.00	**0.26**
AES8	0.02	−0.00	0.07	0.00	0.00	**0.19**

*N* = 1, 232. HSPS, highly sensitive person scale; EOE, ease of excitation; AES, aesthetic sensitivity; LST, low sensory threshold; ASP, adolescent adult sensory profile scale; Ss, sensory sensitivity; SA, sensory avoiding; Se, sensation seeking; LR, low registration. The loadings of each factor are printed in bold.

Although eliminating unstable items would seem to make the differences between all subscales of HSPS and ASP stronger, EGA provided communities containing mixed subscales. Regarding the HSPS communities, the EGA suggested a clear separation of the *Aesthetic Sensitivity* items but an inconclusive division between *Low Sensory Threshold* and *Ease of Excitation*. Regarding the ASP, the EGA differentiated the *Sensation Seeking* and the *Low Registration* items but did not differentiate between *Sensory Avoiding* and *Sensory Sensitivity*. This finding is similar to the results reported by Brown et al. (2001). There was no evidence for differentiation between the *Sensory Avoiding* and *Sensory Sensitivity* profiles. Instead, our results suggest that the items of the *Sensory Sensitivity* and *Sensory Avoiding* subscales are divided by auditory versus non-auditory sensitivity.

### Short scales for HSPS and ASP

Based on the EGA, we constructed new short scales and subscales: A total HSPS 23-item scale, α = 0.92, AES with five items, α = 0.72, EOE and LST subscale with 18 items, α = 0.92, a total ASP 35-item scale, Se with 10 items, α = 0.68, LR with seven items, α = 0.72, non-auditory Ss-SA with 12 items, α = 0.75, auditory Ss-SA with six items, α = 0.84 (see Supplementary Appendix A).

We subjected the above new scales and subscales to item-response theory (IRT) analyses using the *ltm* package in R (Rizopoulos, 2006). IRT analyses might help to offer even shorter scales/subscales. We ordered the items based on the amount of information each item contained in the entire item range. Next, we constructed one-item subscales based on the item with the highest information and added items one at a time until we obtained Cronbach’s α of 0.90 or ran out of items. We found that reducing the 18 EOE and LST items to nine still retains α of 0.90. No other subscale could be improved. Therefore, we used the short EOE and LST to construct a short total HSPS 14-item scale, α = 0.90. For items composing the short scales, see Supplementary Appendix A.^3^

When items are complex (or unstable), it could be either because they genuinely share cross-domain variance or have poor psychometric properties. One way to tease these possibilities is to inspect the scale’s internal consistency. The full 27-item HSPS scale has identical Cronbach’s alpha to the 23-item version based on the EGA (both are 0.92). Thus, the four items are redundant. Moreover, Cronbach’s alpha of the 14-item scale we propose is 0.90, which is pretty good on its own, and demonstrates that the unstable items do not particularly contribute to the measurement of HSPS. Yet, the remainder of our analyses used the 18-item EOE and LST to benefit from its slightly improved reliability, α = 0.92 (see Supplementary Appendix B).

Interestingly, a short 12-item version was already suggested for the 27-items HSPS (Aron and Aron, 1997) and was mentioned in passing by Aron and Aron (2018). Past applications of that short version (Pluess, 2015; Pluess and Boniwell, 2015) yielded α’s = 0.85. We compared this 12-item scale to the 14-item scale developed here. Nine items were identical, while our reliance on the item information replaced three items.^4^

### Divergent validity for the HSPS and ASP

To test the divergent validity of the HSPS and ASP, we first considered Sample 1. We correlated the HSPS and ASP with neuroticism, age, gender, and membership in the HSP Facebook group (yes/ no). We also controlled (covaried out) neuroticism from age, gender, and HSP-Facebook-group membership. For predicting neuroticism and age, we used multiple regression, and for Facebook membership and gender, we used logistic regression.

As shown in Table 2, the disattenuated correlation of HSPS and ASP subscales and neuroticism are 0.80 or below. For EOE and LST subscale, the disattenuated correlation with neuroticism is 0.80, which indicates a marginal problem with divergent validity (Rönkkö and Cho, 2020). To further probe the divergent validity of HSPS, we regressed the membership in the HSPS Facebook group on neuroticism and HSPS (using logistic regression). As shown in Model 1 in Table 3, the main effect of HSPS on Facebook membership is preserved when controlling for both neuroticism and ASP. Specifically, for every increase in one unit on HSPS, the likelihood of belonging to the HSP Facebook group increases by *OR* = 4.03, *p* < 0.001, controlling for the other predictors. Model 2 in Table 3 shows that both HSPS subscales predict membership in the HSP Facebook group controlling for neuroticism. Finally, when controlling for neuroticism, Model 3 in Table 3 shows that both the non-auditory and auditory ASP subscales are positively related to the likelihood of membership in the HSP Facebook group and that the LR scale is negatively related to it. Yet, the effects of the ASP subscales, controlling for neuroticism, are weak relative to the effects of the total HSPS scale, consistent with the low correlations of Se and LR with neuroticism (Table 2).

**Table 2:** Sample 1: Descriptive statistics and correlations (below the diagonal), reliabilities (in the diagonal), and disattenuated correlations (above the diagonal).

Measure	*N*	Mean	SD	1	2	3	4	5	6	7	8	9	10	11
1. Female (1 = yes; 0 = no)	1,232	0.67	0.47	(−)		0.25	0.13	0.26	−0.01	0.01	0.12	0.11	0.21	
2. Age	1,231	31.10	12.52	0.07**	(−)	0.12	0.12	0.11	−0.12	−0.07	0.01	0.07	0.09	
3. HSPS: Total	1,240	5.92	1.73	0.24**	0.12**	(0.92)	**0.76**	**1.07**	−0.03	0.14	0.59	0.70	**0.77**	0.58
4. HSPS: AES	1,240	6.81	1.76	0.11**	0.10**	0.63**	(0.74)	0.56	0.36	−0.19	0.20	0.36	0.37	0.46
5. HSPS: EOE + LST	1,240	5.67	1.95	0.25**	0.11**	0.98**	0.46**	(0.92)	−0.11	0.20	0.62	0.71	**0.80**	0.55
6. ASP: Se	1,240	4.89	1.57	−0.01	−0.10**	−0.02	0.25**	−0.09**	(0.68)	0.24	−0.23	−0.09	−0.17	−0.12
7. ASP: LR	1,240	2.36	1.59	0.01	−0.06*	0.11**	−0.14**	0.16**	0.17**	(0.72)	0.47	0.31	0.20	−0.03
8. ASP: Non-auditory SA + Ss	1,240	3.45	1.53	0.10**	0.01	0.50**	0.15**	0.52**	−0.16**	0.35**	(0.77)	0.67	0.52	0.34
9. ASP: Auditory SA + Ss	1,240	4.49	2.37	0.10**	0.06*	0.61**	0.29**	0.63**	−0.07*	0.24**	0.54**	(0.84)	0.59	0.45
10. Neuroticism	1,240	4.85	2.72	0.19**	0.08**	0.68**	0.29**	0.70**	−0.13**	0.16**	0.41**	0.49**	(0.84)	0.46
11. HSPs Facebook member (1 = yes; 0 = no)	1,240	0.15	0.36	0.09**	0.24**	0.55**	0.39**	0.53**	−0.10**	−0.02	0.29**	0.41**	0.42**	(−)

HSPS: Total, highly sensitive person scale; AES, aesthetic sensitivity; EOE + LST, ease of excitation and low sensory threshold; ASP, adolescent adult sensory profile scale; Se, sensation seeking; LR, low registration; Auditory SA + Ss, sensory sensitivity and sensory avoiding items regarding auditory stimuli; Non-auditory SA + Ss, sensory sensitivity and sensory avoiding items regarding non-auditory stimuli. Values in the diagonal are reliabilities. Values above the diagonal are disattenuated correlations. For pairs of variables for which reliability is unavailable for both, the cell for the disattenuated correlations is left empty. Disattenuated correlations for which the upper limit of their confidence interval > 0.80 are printed in bold and suggests a marginal problem with divergent validity. Disattenuated correlations for which the upper limit of their confidence interval > 0.90 are printed in underscore and bold and suggests a moderate problem with divergent validity. Disattenuated correlations for which the upper limit of their confidence interval > 1 are printed in italics, underscore and bold and suggests a severe problem with divergent validity. The scale means are based on item means and not sums. *p < 0.05. **p < 0.01.

**Table 3:** Sample 1: Logistic regression predicting membership in HSP facebook group from neuroticism, SPS, and SP scales (model 1), neuroticism and SPS subscales (model 2), and neuroticism and SP subscales (model 3).

	Model 1			Model 2			Model 3		
Predictors	OR	CI	*p*	OR	CI	*p*	OR	CI	*p*
Neuroticism	1.15	[1.03, 1.28]	**0.012**	1.17	[1.05, 1.31]	**0.006**	1.54	[1.41, 1.70]	**<0.001**
HSPS	4.03	[3.21, 5.15]	**<0.001**						
ASP	0.85	[0.67, 1.07]	0.169						
EOE + LST				2.56	[2.10, 3.17]	**<0.001**			
Aesthetic				1.54	[1.31, 1.82]	**<0.001**			
Non-auditory							1.21	[1.04, 1.40]	**0.013**
Auditory							1.47	[1.33, 1.63]	**<0.001**
Se							1.02	[0.90, 1.15]	0.791
LR							0.75	[0.66, 0.86]	**<0.001**

*N* = 1,240. HSPS, highly sensitive person scale; AES, aesthetic sensitivity; EOE and LST, ease of excitation and low sensory threshold; Auditory SA + Ss, sensory sensitivity and sensory avoiding items regarding auditory stimuli; Non-auditory SA + Ss, sensory sensitivity and sensory avoiding items regarding non-auditory stimuli; Se, sensation seeking; LR, low registration. All predictors are centered. The loadings of each factor are printed in bold.‑

Next, we tested the divergence of HSPS from neuroticism in Sample 2, where the measure of neuroticism was based on the Big Five rather than on the three items employed in Sample 1. As shown in Table 4,^5^ the disattenuated correlations of HSPS and ASP subscales with neuroticism are 0.66 or below. And specifically for the HSPS subscales, the disattenuated correlation of neuroticism with EOE and LST is 0.66, and 0.06 with AES. Thus, it is not surprising that EOE and LST, like neuroticism, is mostly related to traits involving negative affect (introversion, trait anxiety, trait social anxiety, shyness, and ADHD), and AES is mostly related to traits involving positive affect (openness, agreeableness, and conscientiousness). Nevertheless, note that the disattenuated correlation of AES and openness is 0.82, which indicates a marginal problem with the divergent validity of AES (Rönkkö and Cho, 2020). Still, the disattenuated correlation of EOE and LST and openness is 0.14. Thus, these results are consistent with our review of the constructs and meta-analysis, suggesting divergent validity for the HSPS and ASP. They are not measures of neuroticism.

**Table 4:** Descriptive statistics and correlations for Sample 2 variables of HSPS and ASP sub-scales using EGA.

Measure	*N*	Mean	SD	1	2	3	4	5	6	7	8	9	10	11	12	13	14	15	16	17	18	19	20	21	22	23
1. Gender (1 = Female; 0 = Male)	481	1.66	0.48	(−)		0.19	0.01	0.21	0.02	−0.01	0.15	0.12	0.14	−0.13	0.07	0.12	−0.05	0.01	−0.02	0.02	0.02	−0.03	0.05	0.14	−0.03	0.14
2. Age	481	16.17	16.20	0.08	(−)	−0.04	−0.07	−0.04	0.05	0.05	0.07	0.01	0.04	0.02	−0.14	−0.06	−0.05	0.07	0.06	0.12	0.03	0.05	−0.07	−0.08	0.003	0.02
3. HSPS: Total	471	5.38	1.49	0.18**	−0.04	(0.89)	**0.80**	**1.09**	−0.07	0.13	0.30	0.52	0.59	0.15	−0.07	−0.13	0.28	0.44	0.38	0.39	0.43	0.40	−0.35	−0.01	−0.17	0.51
4. HSPS: AES	471	6.45	1.61	0.01	−0.05	0.61**	(0.64)	0.56	0.27	−0.13	−0.07	0.08	0.06	−0.32	0.29	0.42	**0.82**	−0.07	−0.22	−0.20	0.05	0.07	0.05	0.13	−0.22	0.12
5. HSPS: EOE + LST	471	5.08	1.68	0.20**	−0.03	0.98**	0.42**	(0.90)	−0.14	0.18	0.35	0.56	0.66	0.24	−0.15	−0.24	0.14	0.52	0.48	0.48	0.48	0.44	−0.41	−0.04	−0.14	0.55
7. ASP:Se	480	5.13	1.58	0.02	0.04	−0.05	0.18**	−0.11*	(0.66)	0.22	−0.20	0.02	−0.21	−0.43	0.19	−0.004	0.49	−0.14	−0.24	−0.24	0.06	0.10	−0.09	0.03	−0.36	0.04
8. ASP:LR	480	2.07	1.53	−0.005	0.04	0.11*	−0.09	0.14**	0.15**	(0.71)	0.51	0.41	0.32	0.15	−0.28	−0.53	0.03	0.43	0.37	0.33	0.57	0.57	−0.58	−0.27	−0.07	0.38
9. ASP:Non-auditory SA + Ss	479	3.38	1.45	0.13**	0.06	0.24**	−0.05	0.29**	−0.14**	0.37**	(0.72)	0.53	0.44	0.30	−0.37	−0.24	−0.21	0.40	0.41	0.39	0.45	0.48	−0.36	−0.16	0.20	0.35
10. ASP:Auditory SA + Ss	481	4.00	2.25	0.10*	0.01	0.44**	0.06	0.48**	0.01	0.31**	0.40**	(0.80)	0.43	0.24	−0.23	−0.33	0.08	0.38	0.40	0.40	0.46	0.51	−0.44	−0.15	−0.01	0.46
11. Neuroticism	471	4.36	1.67	0.13**	0.03	0.50**	0.04	0.56**	−0.15**	0.24**	0.34**	0.35**	(0.80)	0.31	−0.50	−0.55	−0.11	**0.83**	0.58	0.58	0.54	0.48	−0.42	−0.09	0.002	0.54
12. Introversion	471	3.95	1.59	−0.11*	0.02	0.12**	−0.22**	0.20**	−0.31**	0.11*	0.22**	0.19**	0.24**	(0.78)	−0.27	−0.35	−0.43	0.48	0.70	0.72	0.25	0.18	−0.19	−0.20	0.34	0.27
13. Agreeableness	471	6.88	1.44	0.06	−0.12**	−0.06	0.20**	−0.12**	0.14**	−0.21**	−0.27**	−0.18**	−0.39**	−0.21**	(0.77)	**0.75**	0.22	−0.53	−0.40	−0.41	−0.42	−0.37	0.44	0.40	−0.28	−0.21
14. Conscientiousness	471	7.16	1.38	0.09*	−0.04	−0.09	0.25**	−0.17**	−0.002	−0.34**	−0.15**	−0.22**	−0.37**	−0.23**	0.49**	(0.56)	0.23	**−0.74**	−0.54	−0.55	−0.66	−0.63	0.58	0.42	−0.11	−0.33
15. Openness	471	6.06	1.52	−0.04	−0.04	0.23**	0.56**	0.11*	0.34**	0.02	−0.15**	0.06	−0.08	−0.33**	0.16**	0.15**	(0.73)	−0.13	−0.20	−0.21	−0.01	0.09	−0.08	−0.02	−0.11	0.05
16. Trait Anxiety	471	3.22	1.63	0.01	0.06	0.40**	−0.06	0.47**	−0.11*	0.35**	0.32**	0.33**	0.71**	0.40**	−0.44**	−0.53**	−0.11*	(0.92)	0.75	**0.76**	0.63	0.58	−0.61	−0.33	0.12	0.59
17. Trait Social Anxiety	471	3.14	1.75	−0.02	0.06	0.35**	−0.17**	0.44**	−0.19**	0.30**	0.34**	0.35**	0.50**	0.60**	−0.34**	−0.39**	−0.17**	0.69**	(0.93)	**0.97**	0.56	0.51	−0.56	−0.33	0.23	0.51
18. Shyness	471	3.42	1.83	0.02	0.12*	0.35**	−0.15**	0.44**	−0.19**	0.27**	0.32**	0.34**	0.50**	0.61**	−0.35**	−0.40**	−0.17**	0.70**	0.90**	(0.93)	0.52	0.48	−0.53	−0.32	0.23	0.53
19. ADHD: BDEFS-SF	480	3.02	1.81	0.02	0.03	0.39**	0.04	0.44**	0.04	0.46**	0.36**	0.40**	0.47**	0.22**	−0.36**	−0.47**	−0.01	0.58**	0.52**	0.48**	(0.92)	**0.95**	−0.59	−0.34	−0.10	0.57
20. ADHD: ASRS5	481	3.29	1.82	−0.03	0.04	0.33**	0.05	0.36**	0.07	0.42**	0.35**	0.39**	0.37**	0.14**	−0.28**	−0.41**	0.06	0.48**	0.42**	0.40**	0.79**	(0.75)	−0.61	−0.38	−0.12	0.61
21. Trait Mindfulness	471	6.82	1.63	0.05	−0.07	−0.31**	0.04	−0.37**	−0.07	−0.46**	−0.29**	−0.37**	−0.36**	−0.16**	0.37**	0.41**	−0.06	−0.55**	−0.51**	−0.48**	−0.53**	−0.50**	(0.88)	0.28	−0.04	−0.43
22. Family support	463	7.14	2.14	0.14**	−0.08	−0.01	0.10*	−0.04	0.02	−0.22**	−0.14**	−0.13**	−0.08	−0.17**	0.34**	0.31**	−0.02	−0.31**	−0.31**	−0.30**	−0.32**	−0.32**	0.25**	(0.94)	−0.24	−0.23
23. Avoidant Attachment	481	4.75	1.97	−0.02	0.003	−0.13**	−0.15**	−0.11*	−0.25**	−0.05	0.15**	−0.01	0.002	0.26**	−0.21**	−0.07	−0.08	0.10*	0.19**	0.19**	−0.08	−0.09*	−0.03	−0.20**	(0.73)	−0.21
24. Attachment Anxiety	481	4.18	2.34	0.13**	0.02	0.44**	0.09	0.47**	0.03	0.29**	0.27**	0.37**	0.44**	0.22**	−0.17**	−0.22**	0.04	0.52**	0.45**	0.47**	0.49**	0.48**	−0.36**	−0.20**	−0.16**	(0.82)

HSPS: Total , highly sensitive person scale; AES , aesthetic sensitivity; EOE + LST , ease of excitation and low sensory threshold; ASP , adolescent adult sensory profile scale; Se , sensation seeking; LR , low registration; Non-auditory SA + Ss , sensory sensitivity and sensory avoiding items regarding non-auditory stimuli; Auditory SA + Ss , sensory sensitivity and sensory avoiding items regarding auditory stimuli. Values in the diagonal are reliabilities. Values above the diagonal are disattenuated correlations. For pairs of variables for which reliability is unavailable for both, the cell for the disattenuated correlations is left empty. Disattenuated correlations for which the upper limit of their confidence interval > 0.80 are printed in bold and suggests a marginal problem with divergent validity. Disattenuated correlations for which the upper limit of their confidence interval > 0.90 are printed in underscore and bold and suggests a moderate problem with divergent validity. Disattenuated correlations for which the upper limit of their confidence interval > 1 are printed in italics, underscore and bold and suggests a severe problem with divergent validity. The scale means are based on item means and not sums. *p < 0.05. **p < 0.01.

Last, family support, avoidance, and anxious attachment styles correlate with most SPS and SP scales in the expected direction (Table 4). For example, the more people recall family support from their childhood (up to the age of 18), the lower their LR. Of these three predictors, the anxious-attachment style is the best predictor of the SPS and SP scales, consistent with the theories that they are related to anxiety in general. Yet, even the strongest corrected correlation, 0.55, between anxious attachment style and EOE and LST, is low enough to support their divergent validity. We tested whether any of these three scales moderated the association of SPS with neuroticism. Inconsistent with previous studies (e.g., Jagiellowicz et al., 2016), none of the interactions were significant, *p* > 0.05 (see Supplementary Table S7 in Section D). Nevertheless, note that previous studies finding an interaction had used measures related primarily to much earlier states of childhood, relative to up to the age of 18.

## Discussion

We assessed the latent structure of the HSPS and ASP and tested their convergence and divergence. The HSPS and ASP were rarely considered together, probably because the HSPS emanated from research in personality psychology and the ASP from occupational therapy. When the latent structures of both HSPS and ASP were examined, the structure differed from the respective original theories (Brown et al., 2001; Smolewska et al., 2006; Aron and Aron, 2010; Booth et al., 2015). First, although the HSPS is meant to be an overall measure of SPS and was considered to reflect a single factor (Aron and Aron, 1997), previous studies suggested three factors (e.g., Smolewska et al., 2006). Thus we used EGA that eliminated unstable items that were common across communities and found that the structure of HSPS includes two communities of EOE and LST and AES. Moreover, some of the EOE and LST items loaded on different communities than the factors found in past research. Four items did not load on any of these (item 12 about conscientiousness and items 17, 18, and 24 about avoiding unpleasant stimuli).

Second, we found that ASP’s structure differs from the original formulation. The original formulation of ASP suggests two axes that create four profiles. However, our data revealed four latent communities. Like the original formulation, we found that LR and Se are separate sub-constructs but that the original SA and Ss subconstructs are indistinguishable empirically. Instead, our results suggest two communities divided by sensitivity to auditory versus non-auditory stimuli.

Furthermore, The combined analyses of HSPS and ASP suggest that the HSPS and ASP share a common variance. The highest commonality is between the HSPS sub-scale of EOE and LST and the ASP sub-scale of auditory sensitivity (a dissatteunated correlation of 0.68). This finding suggests that EOE and LST taps, among other things, sensitivity to noise. Nevertheless, although EOE and LST and auditory sensitivity share sensitivity to noise, their items diverge in intensity. The AES-auditory-sensitivity items refer to being distracted by noise. The EOE and LST items refer to *overarousal* and being *overwhelmed* by noise (and other things). Thus, the EOE and LST items reflect intensive-physical reactions (such as rapid heart rate) to noise, perhaps reaching transmarginal inhibition (Aron and Aron, 1997; Belsky and Pluess, 2009).

Thus, HSPS, ASP, and their subscales may be sibling constructs (Lawson and Robins, 2021). Sibling constructs share meaningful variance but are still distinct. For example, self-esteem and grandiose narcissism share positive self-regard but diverge in their associations with aggression tendencies. Another challenge for the divergent validity of the HSPS subscale of aesthetics is its high correlation with the openness measure of the Big 5. Again, these constructs may be siblings because the items of the AES reflect perceptual sensitivity to details and enjoyment of them. In contrast, openness items reflect an interest in arts, curiosity, and imagination, but not necessarily perceiving physical subtleties. Future research may test the differentiating theoretical mechanism underlying HSPS and ASP subscales and the difference between AES and openness.

Our work addresses the need for subscales in both the HSPS and ASP. Whereas total scale scores are helpful for treatment evaluations,^6^ the more fine-grained sub-scales appeared to have value in predicting external criteria. For example, each of the HSPS sub-scales has a unique explanatory power in predicting the HSP Facebook group membership. This finding calls for future researchers to identify the different potential mechanisms responsible for these distinctions.

Another contribution of our work is demonstrating the utility of the relatively new EGA (Golino and Epskamp, 2017; Golino et al., 2020b). EGA allowed us to uncover the data structure *via* a network graph without assuming a simple structure. This approach helped us address the debate regarding the convergence, or divergence, of the constructs of SPS and SP. EGA also helped delineate the potential divergence between SPS and SP theories.

Specifically, both HSPS and ASP seem to capture elevated sensitivity to stimulations; however, the HSPS seems to capture *emotional* reactions to overstimulation, while the ASP seems to capture *behavioral* reactions to stimulation in general. This divergence^7^ may support the argument that these theories are distinguished (Aron, 2011; Levit-Binnun et al., 2014). SPS seems to be about a tendency to react emotionally due to a deep depth of processing, a great awareness of subtleties, high emotional arousal, and empathic abilities (consistent with Aron and Aron, 1997; Aron et al., 2012; Aron, 2013; Levit-Binnun et al., 2014; Homberg et al., 2016). The SP theory, in contrast, seems to capture not only tendencies of high sensitivities but also different tendencies for low sensitivities (the Se and LR subscales). Therefore, using HSPS and ASP measures together may reveal the consequences of these individual-difference combinations by testing interactions between HSPS subscales and ASP subscales on various outcomes.

Moreover, we addressed the potential criticism that both HSPS and ASP may be nothing more than neuroticism in two ways. First, we ran meta-analyses on existing correlations, and second, we included neuroticism in the present study. The meta-analyses and our study suggested that the correlations between HSPS sub-scales and neuroticism are well below 0.70. This figure supports the divergent validity of the HSPS, according to several proposed criteria of divergent validity (Shaffer et al., 2016; Rönkkö and Cho, 2020). Moreover, the HSPS sub-scale of AES is related chiefly to traits involving positive affect, especially openness, which is negatively correlated with neuroticism. These results are consistent with our review, suggesting that while neuroticism relates only to sensitivity to negative affect, SPS relates to both negative and positive affect sensitivity.

### Implications and limitations

This paper has several strengths, including using a relatively large sample, a robust method for uncovering the factorial structure of SPS and SP (EGA; Golino and Epskamp, 2017; Golino et al., 2020b), the test of divergent validity of SPS and SP from neuroticism and other scales, and the development of newly shortened questionnaires. The theoretical implications of our findings are that SPS probes the *emotional* reactivity to stimulation, whereas SP probes the *behavioral* reactivity to stimulation. This distinction helps to understand the different manifestations of sensitivity to stimulation. Moreover, our findings inform theory concerning the difference between sensation modalities, where auditory sensitivity appears relatively independent from other modalities.

The practical implications of our finding include the suggestion that psychologists and occupation therapists working with issues of sensory sensitivity may get a fuller clinical picture if they use both the ASP and HSPS measures and not one. The benefit of using both measures is that they could shed light on different aspects of the phenomenon. Another practical contribution for therapists and researchers is the potential use of the brief measures reported here. The brief versions are approximately half the length of existing questionnaires, with minor loss in reliability and validity. Moreover, our brief 14-item measurement includes proportionally less negatively worded items (64%) than the 27-item HSPS (79%). Nevertheless, given the relatively large negatively worded ratio, for research purposes, it may be desirable to control for associated negative emotions and experiences, such as overarousal.

A significant limitation of our work is that the test of divergent validity of the self-reported SPS and SP was based on associations with other self-reported measures. Future studies may examine the psychometric properties of the SPS and SP reports by others and consider correlations between self-reported and other-reported measures. In addition, our criterion of HSP-Facebook-group membership was based on convenience rather than theory. Future studies may test the incremental validity of ASP and HSPS against different criteria. For example, one may manipulate sensory overload in the laboratory and test its effect on physiological indicators of distress. Next, one can test first whether EOE and LST moderates the effect of the manipulation. Do those high in EOE and LST show more distress following the manipulation, and does this moderation cannot be wholly accounted for by neuroticism?

Another limitation pointed out by a reviewer is that our sample includes participants who were members of a Facebook group dedicated to HSPs. Those participants might have identified themselves as HSPs based on taking the SPS scale before participating in the study. Therefore, we tested whether this subsample would lead to different conclusions than the remaining sample. First, we computed Tables 5, 6 only for the HSPs and once for the remaining sample. The mean absolute correlation values in these tables were similar: 0.32 for the HSPs sample and 0.31 for the remaining sample. We also reran the EGA calculations of these two samples. These EGA showed some fluctuations from Figures 2, 3, yet the solutions were similar. For example, both separated the SPS from the SP items, EOE and LST from other subscales, AES from EOE and LST, Se from LR and Ss and SA. Therefore, we believe our solution reasonably pertains to low and high sensory sensitivity populations. Yet, future research may seek to replicate our analyses on a large sample of people seeking help for coping with sensory sensitivity. To facilitate such research, our Supplementary Material in Section E includes all of the analyses on the two sub samples.

**Table 5:** Descriptive statistics and correlations for study variables of HSPS and ASP sub-scales using EGA.

Measure	*N*	Mean	SD	1	2	3	4	5	6	7	8	9	10
1. Gender (1 = Female; 0 = Male)	1,694	0.67	0.47	(−)		0.24	0.10	0.25	0.12	−0.001	0.01	0.13	0.11
2. Age	1,693	26.9	15.1	0.08**	(−)	0.13	0.11	0.13	0.01	−0.10	0.01	0.03	0.09
3. HSPS: Total	1,702	5.77	1.69	0.23**	0.13**	(0.92)	**0.77**	**1.07**	0.55	−0.05	0.15	0.53	0.66
4. AES	1,702	6.71	1.73	0.08**	0.09**	0.63**	(0.72)	0.56	0.27	0.32	−0.16	0.15	0.31
5. EOE + LST	1,702	5.51	1.89	0.24**	0.12**	0.98**	0.46**	(0.92)	0.57	−0.13	0.21	0.57	0.68
6. ASP: Total	1,702	3.77	1.03	0.11**	0.01	0.47**	0.21**	0.49**	(0.79)	0.46	**0.84**	**0.99**	**0.86**
7. Se	1,702	4.95	1.58	−0.001	−0.08**	−0.04	0.23**	−0.10**	0.34**	(0.68)	0.20	−0.24	−0.08
8. LR	1,702	2.27	1.56	0.01	0.01	0.12**	−0.12**	0.17**	0.63**	0.14**	(0.72)	0.47	0.33
9. Non-auditory SA + Ss	1,702	3.42	1.49	0.12**	0.02	0.44**	0.11**	0.47**	0.76**	−0.17**	0.34**	(0.75)	0.63
10. Auditory SA + Ss	1,702	4.34	2.35	0.10**	0.08**	0.58**	0.24**	0.60**	0.70**	−0.06*	0.26**	0.50**	(0.84)

HSPS: Total, highly sensitive person scale; AES, aesthetic sensitivity; EOE + LST, ease of excitation and low sensory threshold; ASP: Total, adolescent adult sensory profile scale; Se, sensation seeking; LR, low registration; Auditory SA + Ss, sensory sensitivity and sensory avoiding items regarding auditory stimuli; Non-auditory SA + Ss, sensory sensitivity and sensory avoiding items regarding non-auditory stimuli. Values in the diagonal are reliabilities. Values above the diagonal are disattenuated correlations. For pairs of variables for which reliability is unavailable for both, the cell for the disattenuated correlations is left empty. Disattenuated correlations for which the upper limit of their confidence interval > 0.80 are printed in bold and suggests a marginal problem with divergent validity. Disattenuated correlations for which the upper limit of their confidence interval > 0.90 are printed in underscore and bold and suggests a moderate problem with divergent validity. Disattenuated correlations for which the upper limit of their confidence interval > 1 are printed in italics, underscore and bold and suggests a severe problem with divergent validity. *p < 0.05. **p < 0.01.

**Table 6:** Descriptive statistics and correlations for study variables of HSPS and ASP sub-scales using EGA and IRT.

Measure	*N*	Mean	SD	1	2	3	4	5	6	7	8	9	10
1. Gender (1 = Female; 0 = Male)	1,694	−0.33	0.47	(−)		0.23	0.10	0.24	0.12	−0.001	0.01	0.13	0.11
2. Age	1,693	26.95	15.15	0.08**	(−)	0.15	0.11	0.14	0.01	−0.10	0.01	0.03	0.09
3. HSPS: Total	1,702	5.94	1.77	0.21**	0.14**	(0.88)	**0.88**	**1.07**	0.55	−0.02	0.12	0.52	0.66
4. AES	1,702	6.71	1.73	0.08**	0.09**	0.70**	(0.72)	0.55	0.27	0.32	−0.16	0.15	0.31
5. EOE + LST	1,702	5.51	2.19	0.23**	0.13**	0.95**	0.44**	(0.90)	0.58	−0.16	0.21	0.59	0.70
6. ASP: Total	1,702	3.77	1.03	0.11**	0.01	0.46**	0.21**	0.49**	(0.79)	0.46	**0.84**	**0.99**	**0.86**
7. Se	1,702	4.95	1.58	−0.001	−0.08**	−0.02	0.23**	−0.12**	0.34**	(0.68)	0.20	−0.24	−0.08
8. LR	1,702	2.27	1.56	0.01	0.01	0.09**	−0.12**	0.17**	0.63**	0.14**	(0.72)	0.47	0.33
9. Non-auditory SA + Ss	1,702	3.42	1.49	0.12**	0.02	0.42**	0.11**	0.48**	0.76**	−0.17**	0.34**	(0.75)	0.63
10. Auditory SA + Ss	1,702	4.34	2.35	0.10**	0.08**	0.57**	0.24**	0.61**	0.70**	−0.06*	0.26**	0.50**	(0.84)

HSPS: Total, highly sensitive person scale; AES, aesthetic sensitivity; EOE + LST, ease of excitation and low sensory threshold; ASP: Total, adolescent adult sensory profile scale; Se, sensation seeking; LR, low registration; Non-auditory SA + Ss, sensory sensitivity and sensory avoiding items regarding non-auditory stimuli; Auditory SA + Ss, sensory sensitivity and sensory avoiding items regarding auditory stimuli. Values in the diagonal are reliabilities. Values above the diagonal are disattenuated correlations. For pairs of variables for which reliability is unavailable for both, the cell for the disattenuated correlations is left empty. Disattenuated correlations for which the upper limit of their confidence interval > 0.80 are printed in bold and suggests a marginal problem with divergent validity. Disattenuated correlations for which the upper limit of their confidence interval > 0.90 are printed in underscore and bold and suggests a moderate problem with divergent validity. Disattenuated correlations for which the upper limit of their confidence interval > 1 are printed in italics, underscore and bold and suggests a severe problem with divergent validity. *p < 0.05; **p < 0.01.

## Conclusion

We analyzed the structure of measures designed to reflect similar constructs: sensory-processing sensitivity (measured by HSPS) and sensory thresholds (measured by ASP). Using a relatively large sample, we found that the structure of these instruments diverged from the original theories and that their subscales showed both some convergence and some divergence. Moreover, we have shown that some of the variance captured by these subscales cannot be attributed to neuroticism. In addition, we have shown that several subscales have differential validities, such that each may contribute to the understanding of external criteria. Based on EGA and IRT, we offered brief HSPS and ASP questionnaires halving the number of required items. In concert, our results can be used as a foundation for merging the underlying theories (in occupational therapy and personality psychology) and improving the measurement of individual differences in sensitivity to sensory stimulation, both for practical and research purposes.

## Data availability statement

The original contributions presented in the study are included in the article/Supplementary material, further inquiries can be directed to the corresponding author.

## Ethics statement

Ethical review and approval were obtained from the Ethics Committee at Hebrew University Business School. Participants provided their written informed consent to participate in the study.

## Author contributions

YT-L wrote the first draft of the manuscript and collected the data. All authors performed the statistical analysis and contributed to manuscript revision, read, and approved the submitted version.

## Funding

This research was funded by the Recanati Fund at the Hebrew University Business School and the Israel Science Foundation (grant number 928/17) to ANK.

## Conflict of interest

The authors declare that the research was conducted in the absence of any commercial or financial relationships that could be construed as a potential conflict of interest.

## Publisher’s note

All claims expressed in this article are solely those of the authors and do not necessarily represent those of their affiliated organizations, or those of the publisher, the editors and the reviewers. Any product that may be evaluated in this article, or claim that may be made by its manufacturer, is not guaranteed or endorsed by the publisher.

## Footnotes

^1^This research was not preregistered. All the data and R codes are available at: https://osf.io/783jb/?view_only=b761ecd3badd49fb97f4ed786ed0b25d.

^2^We also performed three different matrix-reduction techniques: principal components analysis (PCA), multiple-dimension scaling (MDS), and network analysis. Given the advantages of EGA in identifying empirical dimensions in multidimensional data, in this paper, we present only the EGA, and the three matrix-reduction analyses presented in supplementary material (see Supplemental Material Section C).

^3^IRT dropped only 9 items from the EOE & LST subscale (1, 3, 4, 5, 6, 13, 20, 26, 27).

^4^Items 2, 7, 12 were replaced with items 3, 9, 13, 15, and 19.

^5^For the analyses in Table 4, we did not impute missing data. We used pair-wise deletion in the correlation matrix to obtain maximum power for each test.

^6^The ASP scale is used as a standardized tool to help evaluate sensory processing patterns and, if found to be needed, adjust the proper occupational-therapy treatment for difficulties in sensory regulation. The HSPS provides a psychological evaluation for SPS and thus assists to conduct a proper psychological-treatment for HSPs’ patients.

^7^With strongest correlations among HSPS subscales and ASP subscales being about 0.70.

## References

[ref1] Acevedo B. P. Aron E. N. Aron A. Sangster M. D. Collins N. Brown L. L. (2014). The highly sensitive brain: an fMRI study of sensory processing sensitivity and response to others' emotions. Brain Behav. 4, 580–594. doi: 10.1002/brb3.242, PMID: 25161824 PMC4086365

[ref2] Aguinis H. Pierce C. A. Culpepper S. A. (2009). Scale coarseness as a methodological artifact: correcting correlation coefficients attenuated from using coarse scales. Organ. Res. Methods 12, 623–652. doi: 10.1177/1094428108318065

[ref3] Ahadi B. Basharpoor S. (2010). Relationship between sensory processing sensitivity, personality dimensions and mental health. J. Appl. Sci. 10, 570–574. doi: 10.3923/jas.2010.570.574

[ref4] Andresen M. Goldmann P. Volodina A. (2017). Do overwhelmed expatriates intend to leave? The effects of sensory processing sensitivity, stress, and social capital on Expatriates' turnover intention. Eur. Manag. Rev. 15, 315–328. doi: 10.1111/emre.12120

[ref6] Aron E. N. (2000). “High sensitivity as one source of fearfulness and shyness: preliminary research and clinical implications” in Extreme Fear, Shyness, and Social Phobia: Origins, Biological Mechanisms, and Clinical Outcomes. ed. Schulkin L. S. J. (New York: Oxford University Press), 251–272.

[ref7] Aron E. N. (2010). Psychotherapy and the Highly Sensitive Person. New York: Routledge.

[ref8] Aron E. N. (2011). Psychotherapy and the Highly Sensitive Person: Improving Outcomes for That Minority of People Who Are the Majority of Clients New York: Routledge.

[ref9] Aron E. N. (2013). The Highly Sensitive Person. eds. E. Aron and A. Aron (Kensington Publishing Corp), 1–4.

[ref10] Aron E. N. Aron A. (1997). Sensory-processing sensitivity and its relation to introversion and emotionality. J. Pers. Soc. Psychol. 73, 345–368. doi: 10.1037/0022-3514.73.2.345, PMID: 9248053

[ref11] Aron A. Aron E. N. (2010). Are There Positive and Negative Facets of High Sensitivity. American Psychological Association, San Diego, CA.

[ref12] Aron E. N. Aron A. (2018). Tips for SPS research. Available at: https://hsperson.com/wp-content/uploads/2018/08/Tips_for_SPS_Research_Revised_July24_2018.pdf (Accessed October 21, 2022).

[ref13] Aron E. N. Aron A. Davies K. M. (2005). Adult shyness: the interaction of temperamental sensitivity and an adverse childhood environment. Personal. Soc. Psychol. Bull. 31, 181–197. doi: 10.1177/0146167204271419, PMID: 15619591

[ref14] Aron E. N. Aron A. Jagiellowicz J. (2012). Sensory processing sensitivity: a review in the light of the evolution of biological responsivity. Personal. Soc. Psychol. Rev. 16, 262–282. doi: 10.1177/1088868311434213 22291044

[ref15] Aron A. Ketay S. Hedden T. Aron E. N. Markus H. R. Gabrieli J. D. (2010). Temperament trait of sensory processing sensitivity moderates cultural differences in neural response. Soc. Cogn. Affect. Neurosci. 5, 219–226. doi: 10.1093/scan/nsq028, PMID: 20388694 PMC2894664

[ref17] Asendorpf J. B. (1990). Development of inhibition during childhood: evidence for situational specificity and a two-factor model. Dev. Psychol. 26, 721–730. doi: 10.1037/0012-1649.26.5.721

[ref18] Bakker K. Moulding R. (2012). Sensory-processing sensitivity, dispositional mindfulness and negative psychological symptoms. Pers. Individ. Differ. 53, 341–346. doi: 10.1016/j.paid.2012.04.006

[ref20] Barkley R. A. (2011). Barkley Deficits in Executive Functioning Scale (BDEFS) New York: Guilford Press.

[ref21] Barnhofer T. Duggan D. S. Griffith J. W. (2011). Dispositional mindfulness moderates the relation between neuroticism and depressive symptoms. Pers. Individ. Differ. 51, 958–962. doi: 10.1016/j.paid.2011.07.032, PMID: 22180693 PMC3191267

[ref22] Belsky J. (1997). Variation in susceptibility to environmental influence: an evolutionary argument. Psychol. Inq. 8, 182–186. doi: 10.1207/s15327965pli0803_3

[ref23] Belsky J. Pluess M. (2009). Beyond diathesis stress: differential susceptibility to environmental influences. Psychol. Bull. 135, 885–908. doi: 10.1037/a0017376, PMID: 19883141

[ref24] Ben-Avi N. Almagor M. Engel-Yeger B. (2012). Sensory processing difficulties and interpersonal relationships in adults: an exploratory study. Psychology 03, 70–77. doi: 10.4236/psych.2012.31012

[ref25] Benham G. (2006). The highly sensitive person: stress and physical symptom reports. Personal. Individ. Differ. 40, 1433–1440. doi: 10.1016/j.paid.2005.11.021

[ref26] Biederman J. Faraone S. V. Monuteaux M. C. Bober M. Cadogen E. (2004). Gender effects on attention-deficit/hyperactivity disorder in adults, revisited. Biol. Psychiatry 55, 692–700. doi: 10.1016/j.biopsych.2003.12.003, PMID: 15038997

[ref27] Booth C. Standage H. Fox E. (2015). Sensory-processing sensitivity moderates the association between childhood experiences and adult life satisfaction. Personal. Individ. Differ. 87, 24–29. doi: 10.1016/j.paid.2015.07.020, PMID: 26688599 PMC4681093

[ref28] Borenstein M. Hedges L. V. Higgins J. P. T. Rothstein H. R. (2021). Introduction to Meta-Analysis. Hoboken, NJ: John Wiley & Sons doi: 10.1002/9781119558378.

[ref29] Boyce W. T. Ellis B. J. (2005). Biological sensitivity to context: I. an evolutionary–developmental theory of the origins and functions of stress reactivity. Dev. Psychopathol. 17, 271–301. doi: 10.1017/s0954579405050145 16761546

[ref30] Brindle K. Moulding R. Bakker K. Nedeljkovic M. (2015). Is the relationship between sensory-processing sensitivity and negative affect mediated by emotional regulation? Aust. J. Psychol. 67, 214–221. doi: 10.1111/ajpy.12084

[ref31] Brown C. Dunn W. (2002). Adolescent-Adult Sensory Profile: User's Manual. Little Rock, AR: Therapy Skill Builders San Antonio.

[ref32] Brown K. W. Ryan R. M. (2003). The benefits of being present: mindfulness and its role in psychological well-being. J. Pers. Soc. Psychol. 84, 822–848. doi: 10.1037/0022-3514.84.4.822, PMID: 12703651

[ref33] Brown C. Stoffel V. C. (2010). Occupational Therapy in Mental Health: A Vistion for Participation. ed. C. A. Fratantoro. (FA Davis Company).

[ref34] Brown C. Tollefson N. Dunn W. Cromwell R. Filion D. (2001). The adult sensory profile: measuring patterns of sensory processing. Am. J. Occup. Ther. 55, 75–82. doi: 10.5014/ajot.55.1.75, PMID: 11216370

[ref35] Carlson L. E. Brown K. W. (2005). Validation of the mindful attention awareness scale in a cancer population. J. Psychosom. Res. 58, 29–33. doi: 10.1016/j.jpsychores.2004.04.366, PMID: 15771867

[ref36] Catak P. D. (2012). The Turkish version of mindful attention awareness scale: preliminary findings. Mindfulness 3, 1–9. doi: 10.1007/s12671-011-0072-3

[ref37] Chamberlain S. R. Ioannidis K. Leppink E. W. Niaz F. Redden S. A. Grant J. E. (2017). ADHD symptoms in non-treatment seeking young adults: relationship with other forms of impulsivity. CNS Spectr. 22, 22–30. doi: 10.1017/S1092852915000875, PMID: 27680974 PMC5330410

[ref38] Cheek J. M. Krasnoperova E. N. (1999). “Varieties of shyness in adolescence and adulthood”, in Extreme fear, shyness, and social phobia: Origins, biological mechanisms, and clinical outcomes eds. L. A. Schmidt and J. Schulkin (Oxford University Press), 224–250.

[ref39] Cheek J. M. Melchior L. A. (1985). “Measuring the three components of shyness” in Emotion, Personality, and Personal Well-Being II: Symposium conducted at the annual convention of the American Psychological Association. eds. Davis M. H. Franzoi S. L. (Los Angeles)

[ref40] Christensen A. P. Golino H. (2021). Estimating the stability of psychological dimensions via bootstrap exploratory graph analysis: a Monte Carlo simulation and tutorial. Psychol. 3, 479–500. doi: 10.3390/psych3030032

[ref41] Chung J. (2006). Measuring sensory processing patterns of older Chinese people: psychometric validation of the adult sensory profile. Aging Ment. Health 10, 648–655. doi: 10.1080/13607860600648080, PMID: 17050094

[ref43] Costa P. T. McCrae R. R. (1985). The NEO Personality Inventory.

[ref44] Crosbie J. Arnold P. Paterson A. Swanson J. Dupuis A. Li X. . (2013). Response inhibition and ADHD traits: correlates and heritability in a community sample. J. Abnorm. Child Psychol. 41, 497–507. doi: 10.1007/s10802-012-9693-9, PMID: 23315233 PMC3600128

[ref45] Dean E. (2015). Sensory Processing Predictors of Challenging Behavior University of Kansas.

[ref46] Deng Y.-Q. Li S. Tang Y.-Y. Zhu L.-H. Ryan R. Brown K. (2012). Psychometric properties of the Chinese translation of the mindful attention awareness scale (MAAS). Mindfulness 3, 10–14. doi: 10.1007/s12671-011-0074-1

[ref47] Dunn W. (1997). The impact of sensory processing abilities on the daily lives of young children and their families: a conceptual model. Infants Young Child. 9, 23–35. doi: 10.1097/00001163-199704000-00005

[ref48] Dunn W. (2001). The sensations of everyday life: empirical, theoretical, and pragmatic considerations. Am. J. Occup. Ther. 55, 608–620. doi: 10.5014/ajot.55.6.608, PMID: 12959225

[ref49] Dunn W. (2014). Sensory Profile 2: Strengths Based Approach to Assessment and Planning.

[ref50] Engel-Yeger B. (2012). Validating the adolescent/adult sensory profile and examining its ability to screen sensory processing difficulties among Israeli people. Br. J. Occup. Ther. 75, 321–329. doi: 10.4276/030802212X13418284515839

[ref51] Eysenck H. J. (1957). Dynamics of anxiety and hysteria. Bull. Br. Psychol. Soc. 32:51.

[ref52] Eysenck H. J. (1981). A model for Personality. New York: SpringerVerlag, doi: 10.1007/978-3-642-67783-0. .

[ref53] Eysenck H. J. Eysenck S. B. G. (1975). Eysenck Personality Questionnaire Manual. San Diego, CA: Educational and Industrial Testing Service.

[ref54] Fergus T. A. Valentiner D. P. McGrath P. B. Gier-Lonsway S. L. Kim H.-S. (2012). Short forms of the social interaction anxiety scale and the social phobia scale. J. Pers. Assess. 94, 310–320. doi: 10.1080/00223891.2012.660291, PMID: 22369684

[ref55] Flannery A. J. Luebbe A. M. Becker S. P. (2017). Sluggish cognitive tempo is associated with poorer study skills, more executive functioning deficits, and greater impairment in college students. J. Clin. Psychol. 73, 1091–1113. doi: 10.1002/jclp.22406, PMID: 27764528 PMC5398959

[ref56] Friedman S. R. Rapport L. J. Lumley M. Tzelepis A. VanVoorhis A. Stettner L. . (2003). Aspects of social and emotional competence in adult attention-deficit/hyperactivity disorder. Neuropsychology 17, 50–58. doi: 10.1037/0894-4105.17.1.50, PMID: 12597073

[ref57] Gaub M. Carlson C. L. (1997). Behavioral characteristics of DSM-IV ADHD subtypes in a school-based population. J. Abnorm. Child Psychol. 25, 103–111. doi: 10.1023/A:1025775311259 9109027

[ref58] Golino H. Christensen A. P. Moulder R. (2020a). EGAnet: exploratory graph analysis: a framework for estimating the number of dimensions in multivariate data using network psychometrics. R Package Version 02. Available at: https://CRAN.R-project.org/package=EGAnet

[ref59] Golino H. Epskamp S. (2017). Exploratory graph analysis: a new approach for estimating the number of dimensions in psychological research. PLoS One 12:e0174035. doi: 10.1371/journal.pone.0174035, PMID: 28594839 PMC5465941

[ref60] Golino H. Shi D. Christensen A. P. Garrido L. E. Nieto M. D. Sadana R. . (2020b). Investigating the performance of exploratory graph analysis and traditional techniques to identify the number of latent factors: a simulation and tutorial. Psychol. Methods 25, 292–320. doi: 10.1037/met0000255, PMID: 32191105 PMC7244378

[ref500] Greven C. U. Lionetti F. Booth C. Aron E. N. Fox E. Schendan H. E. . (2019). Sensory processing sensitivity in the context of environmental sensitivity: A critical review and development of research agenda. Neurosci. Biobeh. Rev. 98, 287–305., PMID: 30639671 10.1016/j.neubiorev.2019.01.009

[ref61] Grimen H. L. Diseth Å. (2016). Sensory processing sensitivity: factors of the highly sensitive person scale and their relationships to personality and subjective health complaints. Comprehen. Psychol. 5:6007. doi: 10.1177/2165222816660077 27562694

[ref62] Haas B. W. Constable R. T. Canli T. (2008). Stop the sadness: neuroticism is associated with sustained medial prefrontal cortex response to emotional facial expressions. NeuroImage 42, 385–392. doi: 10.1016/j.neuroimage.2008.04.027, PMID: 18511299 PMC2789588

[ref63] Hodgens J. B. Cole J. Boldizar J. (2000). Peer-based differences among boys with ADHD. J. Clin. Child Psychol. 29, 443–452. doi: 10.1207/S15374424JCCP2903_15, PMID: 10969428

[ref64] Hofmann S. G. Bitran S. (2007). Sensory-processing sensitivity in social anxiety disorder: relationship to harm avoidance and diagnostic subtypes. J. Anxiety Disord. 21, 944–954. doi: 10.1016/j.janxdis.2006.12.003, PMID: 17241764 PMC2174907

[ref65] Homberg J. R. Schubert D. Asan E. Aron E. N. (2016). Sensory processing sensitivity and serotonin gene variance: insights into mechanisms shaping environmental sensitivity. Neurosci. Biobehav. Rev. 71, 472–483. doi: 10.1016/j.neubiorev.2016.09.029, PMID: 27697602

[ref66] Howarth E. (1986). Introversion and neuroticism: a persistent relationship. Psychol. Rep. 58, 389–390. doi: 10.2466/pr0.1986.58.2.389, PMID: 3704042

[ref67] Jagiellowicz J. Aron A. Aron E. N. (2016). Relationship between the temperament trait of sensory processing sensitivity and emotional reactivity. Soc. Behav. Pers. Int. J. 44, 185–199. doi: 10.2224/sbp.2016.44.2.185

[ref68] Jagiellowicz J. Xu X. Aron A. Aron E. N. Cao G. Feng T. . (2010). The trait of sensory processing sensitivity and neural responses to changes in visual scenes. Soc. Cogn. Affect. Neurosci. 6, 38–47. doi: 10.1093/scan/nsq001, PMID: 20203139 PMC3023077

[ref69] Jensen S. A. Rosen L. (2004). Emotional reactivity in children with attention-deficit/hyperactivity disorder. J. Atten. Disord. 8, 53–61. doi: 10.1177/108705470400800203 15801335

[ref70] Jerome E. M. Liss M. (2005). Relationships between sensory processing style, adult attachment, and coping. Personal. Individ. Differ. 38, 1341–1352. doi: 10.1016/j.paid.2004.08.016

[ref71] John O. P. Donahue E. M. Kentle R. L. (1991). Big five inventory. J. Pers. Soc. Psychol. 60, 348–361. PMID: 2027078

[ref72] John O. P. Naumann L. P. Soto C. J. (2008). Paradigm shift to the integrative big five trait taxonomy. Handb. Pers, Theory Res. 3, 114–158.

[ref700] John O. P. Srivastava S. (1999). The Big Five Trait taxonomy: History, measurement, and theoretical perspectives, in Handbook of personality: Theory and research. eds. L. A. Pervin and O. P. John Guilford Press, 102–138.

[ref73] Kabat-Zinn J. (2003). Mindfulness-based interventions in context: past, present, and future. Clin. Psychol. Sci. Pract. 10, 144–156. doi: 10.1093/clipsy.bpg016

[ref74] Kagan J. (2001). Temperamental Contributions to Affective and Behavioral Profiles in Childhood. eds. S. G. Hofmann and P. M. DiBartolo.

[ref75] Lace J. W. McGrath A. Merz Z. C. (2020). A factor analytic investigation of the Barkley deficits in executive functioning scale, short form. Curr. Psychol. 41, 1–9. doi: 10.1007/s12144-020-00756-7

[ref76] Lafontaine M.-F. Brassard A. Lussier Y. Valois P. Shaver P. R. Johnson S. M. (2016). Selecting the best items for a short-form of the Experiences in Close Relationships questionnaire. Eur. J. Psychol. Assess. 32, 140–154 doi: 10.1027/1015-5759/a000243

[ref77] Lawson K. M. Robins R. W. (2021). Sibling constructs: what are they, why do they matter, and how should you handle them? Personal. Soc. Psychol. Rev. 25, 344–366. doi: 10.1177/10888683211047101, PMID: 34663112

[ref78] Le Blanc A. L. Bruce L. C. Heimberg R. G. Hope D. A. Blanco C. Schneier F. R. . (2014). Evaluation of the psychometric properties of two short forms of the social interaction anxiety scale and the social phobia scale. Assessment 21, 312–323. doi: 10.1177/1073191114521279, PMID: 24497625

[ref79] Levit-Binnun N. Szepsenwol O. Stern-Ellran K. Engel-Yeger B. (2014). The relationship between sensory responsiveness profiles, attachment orientations, and anxiety symptoms. Aust. J. Psychol. 66, 233–240. doi: 10.1111/ajpy.12064

[ref80] Lionetti F. Aron A. Aron E. N. Burns G. L. Jagiellowicz J. Pluess M. (2018). Dandelions, tulips and orchids: evidence for the existence of low-sensitive, medium-sensitive and high-sensitive individuals. Transl. Psychiatry 8, 1–11. doi: 10.1038/s41398-017-0090-6 29353876 PMC5802697

[ref81] Lionetti F. Pastore M. Moscardino U. Nocentini A. Pluess K. Pluess M. (2019). Sensory processing sensitivity and its association with personality traits and affect: a meta-analysis. J. Res. Pers. 81, 138–152. doi: 10.1016/j.jrp.2019.05.013

[ref82] Liss M. Mailloux J. Erchull M. J. (2008). The relationships between sensory processing sensitivity, alexithymia, autism, depression, and anxiety. Pers. Individ. Differ. 45, 255–259. doi: 10.1016/j.paid.2008.04.009

[ref83] Liss M. Timmel L. Baxley K. Killingsworth P. (2005). Sensory processing sensitivity and its relation to parental bonding, anxiety, and depression. Pers. Individ. Differ. 39, 1429–1439. doi: 10.1016/j.paid.2005.05.007

[ref84] Listou Grimen H. Diseth Å. (2016). Sensory processing sensitivity: factors of the highly sensitive person scale and their relationships to personality and subjective health complaints. Percept. Mot. Skills 123, 637–653. doi: 10.1177/0031512516666114 27562694

[ref85] Little L. M. Dean E. Tomchek S. Dunn W. (2018). Sensory processing patterns in autism, attention deficit hyperactivity disorder, and typical development. Phys. Occup. Ther. Pediatr. 38, 243–254. doi: 10.1080/01942638.2017.1390809, PMID: 29240517

[ref86] MacKillop J. Anderson E. J. (2007). Further psychometric validation of the mindful attention awareness scale (MAAS). J. Psychopathol. Behav. Assess. 29, 289–293. doi: 10.1007/s10862-007-9045-1

[ref87] Maté G. (2000). Scattered: How Attention Deficit Disorder Originates and What You Can Do About It. Plume, New York: Penguin.

[ref88] Mattick R. P. Clarke J. C. (1998). Development and validation of measures of social phobia scrutiny fear and social interaction anxiety. Behav. Res. Ther. 36, 455–470. doi: 10.1016/S0005-7967(97)10031-6, PMID: 9670605

[ref89] McCracken L. M. Zhao-O’Brien J. (2010). General psychological acceptance and chronic pain: there is more to accept than the pain itself. Eur. J. Pain 14, 170–175. doi: 10.1016/j.ejpain.2009.03.004, PMID: 19349199

[ref90] McCrae R. R. (1990). Controlling neuroticism in the measurement of stress. Stress Med. 6, 237–241. doi: 10.1002/smi.2460060309

[ref91] Melchior L. A. Cheek J. M. (1990). Shyness and anxious self-preoccupation during a social interaction. J. Soc. Behav. Pers. 5, 117–130.

[ref92] Meredith P. J. Bailey K. J. Strong J. Rappel G. (2016). Adult attachment, sensory processing, and distress in healthy adults. Am. J. Occup. Ther. 70:1736. doi: 10.5014/ajot.2016.017376 26709426

[ref93] Meyer B. Ajchenbrenner M. Bowles D. P. (2005). Sensory sensitivity, attachment experiences, and rejection responses among adults with borderline and avoidant features. J. Pers. Disord. 19, 641–658. doi: 10.1521/pedi.2005.19.6.641, PMID: 16553560

[ref600] Meyer B. Carver C. S. (2000). Negative childhood accounts, sensitivity, and pessimism: A study of avoidant personality disorder features in college students. J. Pers. Disord. 14, 233–248. doi: 10.1521/pedi.2000.14.3.233 11019747

[ref94] Miller J. J. Fletcher K. Kabat-Zinn J. (1995). Three-year follow-up and clinical implications of a mindfulness meditation-based stress reduction intervention in the treatment of anxiety disorders. Gen. Hosp. Psychiatry 17, 192–200. doi: 10.1016/0163-8343(95)00025-M, PMID: 7649463

[ref95] Minshew N. J. Hobson J. A. (2008). Sensory sensitivities and performance on sensory perceptual tasks in high-functioning individuals with autism. J. Autism Dev. Disord. 38, 1485–1498. doi: 10.1007/s10803-007-0528-4, PMID: 18302014 PMC3077539

[ref96] Mullet D. Rinn A. Jett N. Nyikos T. (2016). Sensory processing sensitivity among higher ability adults: a psychometric evaluation of two versions of the highly sensitive person scale. 1–15. Retirado de: http://researchgate.net

[ref97] Neal J. A. Edelmann R. J. Glachan M. (2002). Behavioural inhibition and symptoms of anxiety and depression: is there a specific relationship with social phobia? Br. J. Clin. Psychol. 41, 361–374. doi: 10.1348/014466502760387489 12437791

[ref98] Ormel J. Wohlfarth T. (1991). How neuroticism, long-term difficulties, and life situation change influence psychological distress: a longitudinal model. J. Pers. Soc. Psychol. 60, 744–755. doi: 10.1037/0022-3514.60.5.744, PMID: 2072254

[ref99] Osman A. Gutierrez P. M. Barrios F. X. Kopper B. A. Chiros C. E. (1998). The social phobia and social interaction anxiety scales: evaluation of psychometric properties. J. Psychopathol. Behav. Assess. 20, 249–264. doi: 10.1023/A:1023067302227

[ref100] Panagiotidi M. Overton P. G. Stafford T. (2020). The relationship between sensory processing sensitivity and attention deficit hyperactivity disorder traits: a spectrum approach. Psychiatry Res. 293:113477. doi: 10.1016/j.psychres.2020.113477, PMID: 33198048

[ref101] Pavlov I. P. (1927/1960). Conditioned Reflexes: An Investigation of the Physiological Activity of the Cerebral Cortex. London: Oxford University Press. 10.5214/ans.0972-7531.1017309 PMC4116985 25205891

[ref102] Penley J. A. Tomaka J. (2002). Associations among the big five, emotional responses, and coping with acute stress. Pers. Individ. Differ. 32, 1215–1228. doi: 10.1016/S0191-8869(01)00087-3

[ref103] Pluess M. (2015). Individual differences in environmental sensitivity. Child Dev. Perspect. 9, 138–143. doi: 10.1111/cdep.12120

[ref104] Pluess M. Assary E. Lionetti F. Lester K. J. Krapohl E. Aron E. N. . (2018). Environmental sensitivity in children: development of the highly sensitive child scale and identification of sensitivity groups. Dev. Psychol. 54, 51–70. doi: 10.1037/dev0000406, PMID: 28933890

[ref105] Pluess M. Belsky J. (2013). Vantage sensitivity: individual differences in response to positive experiences. Psychol. Bull. 139, 901–916. doi: 10.1037/a0030196, PMID: 23025924

[ref106] Pluess M. Boniwell I. (2015). Sensory-processing sensitivity predicts treatment response to a school-based depression prevention program: evidence of vantage sensitivity. Personal. Individ. Differ. 82, 40–45. doi: 10.1016/j.paid.2015.03.011

[ref107] Pohl P. S. Dunn W. Brown C. (2003). The role of sensory processing in the everyday lives of older adults. OTJR 23, 99–106. doi: 10.1177/153944920302300303

[ref108] Polner B. Aichert D. Macare C. Costa A. Ettinger U. (2015). Gently restless: association of ADHD-like traits with response inhibition and interference control. Eur. Arch. Psychiatry Clin. Neurosci. 265, 689–699. doi: 10.1007/s00406-014-0531-7, PMID: 25209569

[ref109] Procidano M. E. Heller K. (1983). Measures of perceived social support from friends and from family: three validation studies. Am. J. Community Psychol. 11, 1–24. doi: 10.1007/BF00898416, PMID: 6837532

[ref110] Questionnaire (n.d.). European journal of psychological assessment Psychometrika monograph supplement 32, 140–154.

[ref42] R Core Team (2018). R: A Language and Environment for Statistical Computing. Vienna, Austria: R Foundation for Statistical Computing http://www.R-project.org/

[ref111] Rizopoulos D. (2006). Ltm: an R package for latent variable modeling and item response analysis 17, 25. doi: 10.18637/jss.v017.i05,

[ref112] Robbins C. A. (2005). ADHD couple and family relationships: enhancing communication and understanding through imago relationship therapy. J. Clin. Psychol. 61, 565–577. doi: 10.1002/jclp.20120, PMID: 15723423

[ref113] Rönkkö M. Cho E. (2020). An updated guideline for assessing discriminant validity. Organ. Res. Methods 25, 6–14. doi: 10.1177/1094428120968614

[ref114] Sanz-Cervera P. Pastor-Cerezuela G. González-Sala F. Tárraga-Mínguez R. Fernández-Andrés M.-I. (2017). Sensory processing in children with autism spectrum disorder and/or attention deficit hyperactivity disorder in the home and classroom contexts. Front. Psychol. 8:1772. doi: 10.3389/fpsyg.2017.01772, PMID: 29075217 PMC5641858

[ref115] Sesti A.-M. (2000). State trait anxiety inventory (STAI) in medication clinical trials. Qual. Life Newsl. 25, 15–16.

[ref900] Shaffer J. A. DeGeest D. Li A. (2016). Tackling the problem of construct proliferation: A guide to assessing the discriminant validity of conceptually related constructs. Organ. Res. Methods 19, 80–110., PMID: 26688599

[ref116] Smolewska K. A. McCabe S. B. Woody E. Z. (2006). A psychometric evaluation of the highly sensitive person scale: the components of sensory-processing sensitivity and their relation to the BIS/BAS and “big five”. Personal. Individ. Differ. 40, 1269–1279. doi: 10.1016/j.paid.2005.09.022

[ref117] Sobocko K. Zelenski J. M. (2015). Trait sensory-processing sensitivity and subjective well-being: distinctive associations for different aspects of sensitivity. Personal. Individ. Differ. 83, 44–49. doi: 10.1016/j.paid.2015.03.045

[ref118] Solden S. (2012). Women with attention deficit disorder: embrace your differences and transform your life. eBookIt. com. 1–354. Available at: https://books.google.co.il/books?id=Qm7F2-ZL5IEC

[ref119] Somma A. Adler L. A. Gialdi G. Arteconi M. Cotilli E. Fossati A. (2021). The validity of the World Health Organization adult attention-deficit/hyperactivity disorder self-report screening scale for diagnostic and statistical manual of mental disorders, in adolescence. J. Child Adolesc. Psychopharmacol. 31, 631–638. doi: 10.1089/cap.2020.0158, PMID: 34166067

[ref800] Spielberger C. D. (1983). State-trait anxiety inventory for adults (STAI-AD). APA PsycTests. doi: 10.1037/t06496-000

[ref120] Stormont M. (1998). Family factors associated with externalizing disorders in preschoolers. J. Early Interv. 21, 232–251. doi: 10.1177/105381519802100305

[ref121] Stormont M. (2001). Social outcomes of children with AD/HD: contributing factors and implications for practice. Psychol. Sch. 38, 521–531. doi: 10.1002/pits.1040

[ref122] Strelau J. (1987). Emotion as a key concept in temperament research. J. Res. Pers. 21, 510–528. doi: 10.1016/0092-6566(87)90037-7

[ref123] Strelau J. (1994). “The concepts of arousal and arousability as used in temperament studies” in Temperament: Individual Differences at the Interface of Biology and Behavior. eds. Bates J. E. Wachs T. D. (Washington, DC: American Psychological Association), 117–141.

[ref124] Üçgül M. Ş. Karahan S. Öksüz Ç. (2017). Reliability and validity study of Turkish version of adolescent/adult sensory profile. Br. J. Occup. Ther. 80, 510–516. doi: 10.1177/0308022617706680

[ref125] Uekermann J. Kraemer M. Abdel-Hamid M. Schimmelmann B. G. Hebebrand J. Daum I. . (2010). Social cognition in attention-deficit hyperactivity disorder (ADHD). Neurosci. Biobehav. Rev. 34, 734–743. doi: 10.1016/j.neubiorev.2009.10.009 19857516

[ref126] Ustun B. Adler L. A. Rudin C. Faraone S. V. Spencer T. J. Berglund P. . (2017). The World Health Organization adult attention-deficit/hyperactivity disorder self-report screening scale for DSM-5. JAMA Psychiat. 74, 520–527. doi: 10.1001/jamapsychiatry.2017.0298, PMID: 28384801 PMC5470397

[ref127] Van den Noortgate W. Lopez-Lopez J. A. Marin-Martinez F. Sanchez-Meca J. (2013). Three-level meta-analysis of dependent effect sizes. Behav. Res. Methods 45, 576–594. doi: 10.3758/s13428-012-0261-6, PMID: 23055166

[ref128] Viechtbauer W. (2010). Conducting meta-analyses in r with the metafor package. J. Stat. Softw. 36, 1–48. doi: 10.18637/jss.v036.i03

[ref129] Watson D. Clark L. A. (1984). Negative affectivity: the disposition to experience aversive emotional states. Psychol. Bull. 96, 465–490. doi: 10.1037/0033-2909.96.3.465, PMID: 6393179

[ref130] Weinstein N. Brown K. W. Ryan R. M. (2009). A multi-method examination of the effects of mindfulness on stress attribution, coping, and emotional well-being. J. Res. Pers. 43, 374–385. doi: 10.1016/j.jrp.2008.12.008

[ref131] Wender P. H. Wolf L. E. Wasserstein J. (2001). Adults with ADHD: an overview. Ann. N. Y. Acad. Sci. 931, 1–16. doi: 10.1111/j.1749-6632.2001.tb05770.x 11462736

[ref132] Weyn S. Van Leeuwen K. Pluess M. Lionetti F. Greven C. U. Goossens L. . (2019). Psychometric properties of the highly sensitive child scale across developmental stage, gender, and country. Curr. Psychol. 40, 1–17. doi: 10.1007/s12144-019-00254-5

[ref133] Wheeler Maedgen J. Carlson C. L. (2000). Social functioning and emotional regulation in the attention deficit hyperactivity disorder subtypes. J. Clin. Child Psychol. 29, 30–42. doi: 10.1207/S15374424jccp2901_4, PMID: 10693030

[ref134] Zobel A. Barkow K. Schulze-Rauschenbach S. Von Widdern O. Metten M. Pfeiffer U. . (2004). High neuroticism and depressive temperament are associated with dysfunctional regulation of the hypothalamic–pituitary–adrenocortical system in healthy volunteers. Acta Psychiatr. Scand. 109, 392–399. doi: 10.1111/j.1600-0447.2004.00313.x, PMID: 15049775

