# Supplementary material for: Sensory-processing sensitivity versus the sensory-processing theory: Convergence and divergence
Source: Front Psychol. 2022 Dec 1;13:1010836. doi: 10.3389/fpsyg.2022.1010836 (PMC9752870; doi:10.3389/fpsyg.2022.1010836)
Supplement: Supplementary file 1 [file Data_Sheet_1.PDF]

Section A

Figure S1

A Forest Plot of Correlations Between Total HSPS Scale and Neuroticism

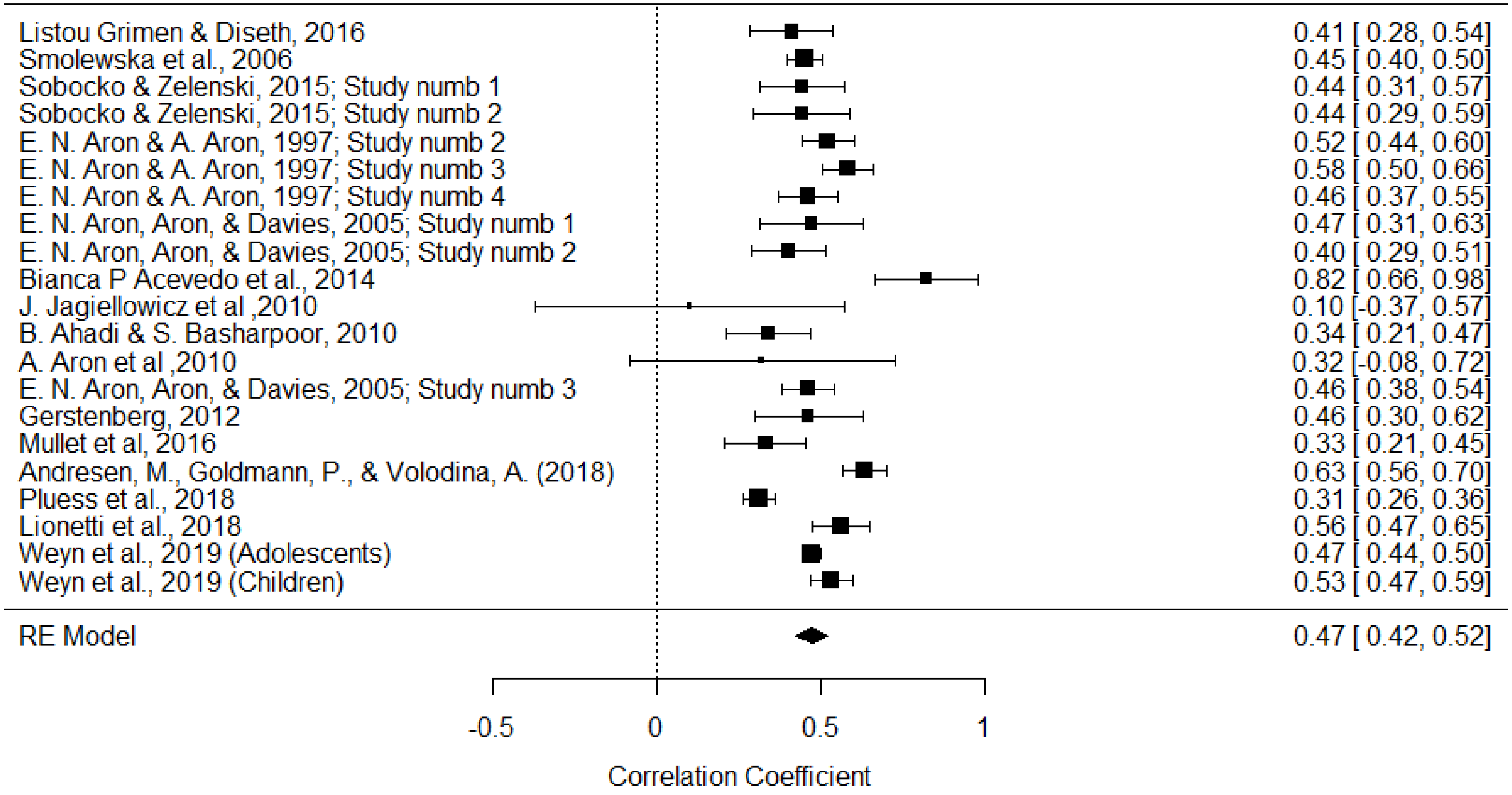

Note. $K = 21$, total $N = 8{,}494$. HSPS = Highly Sensitive Person Scale.

Figure S2

A Forest Plot of Correlations Between Neuroticism with Ease of Excitation

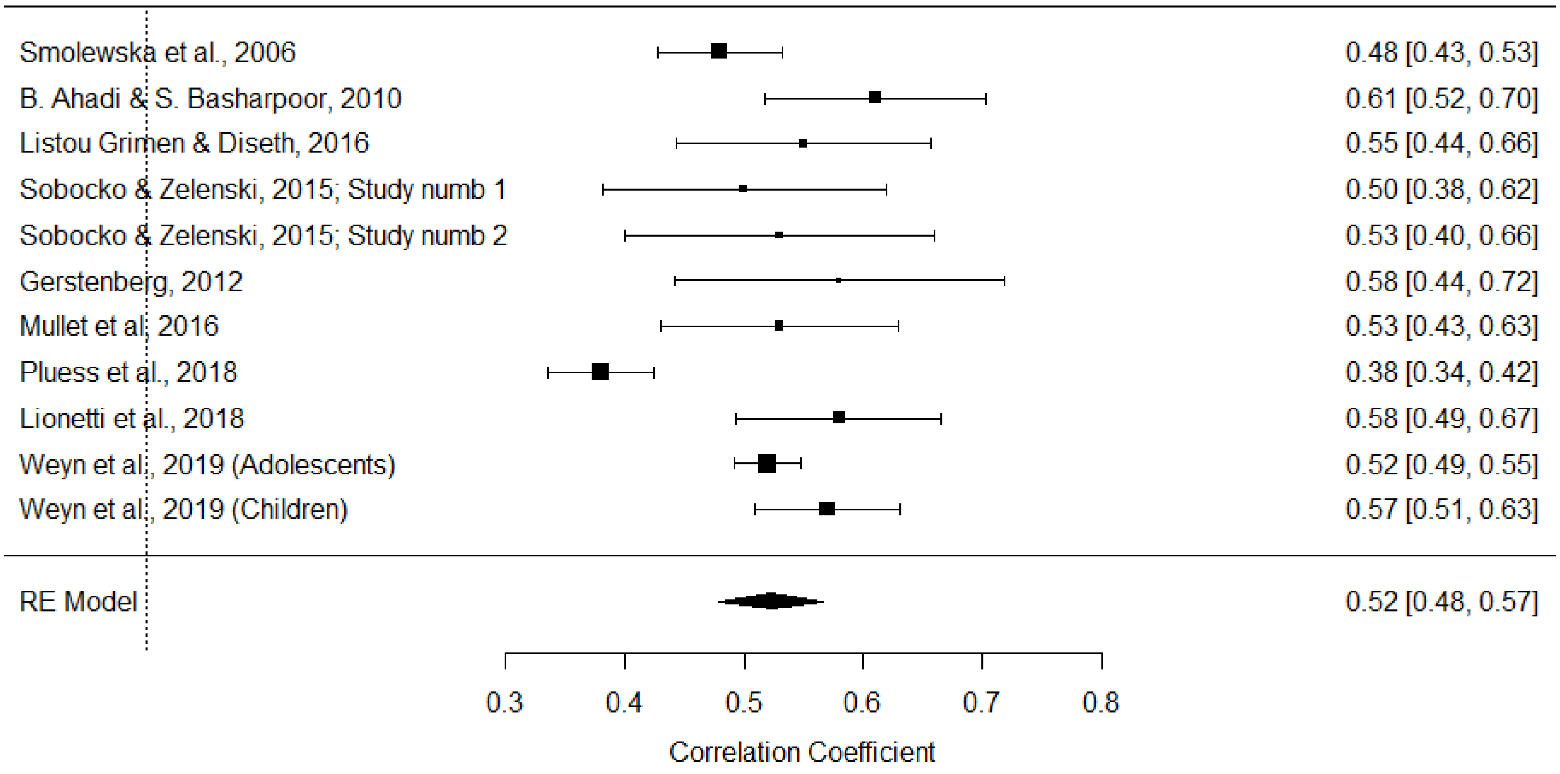

Note. $K = 11$, $N = 6{,}519$.

Figure S3

A Forest Plot of Correlations Between Neuroticism with Low Sensory Threshold

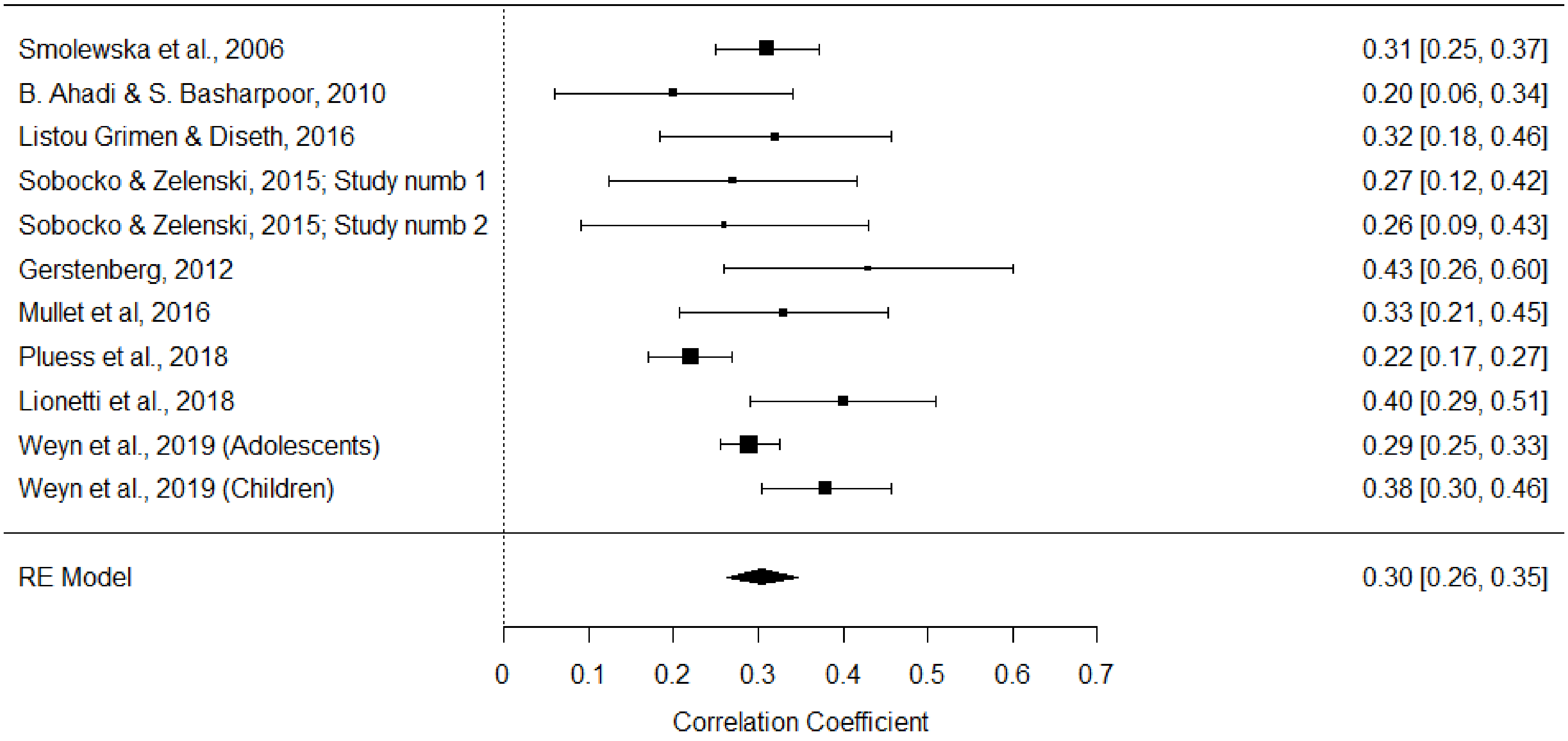

Note. K = 11, N = 6,519.

Figure S4

A Forest Plot of Correlations Between Neuroticism with Aesthetic Sensitivity

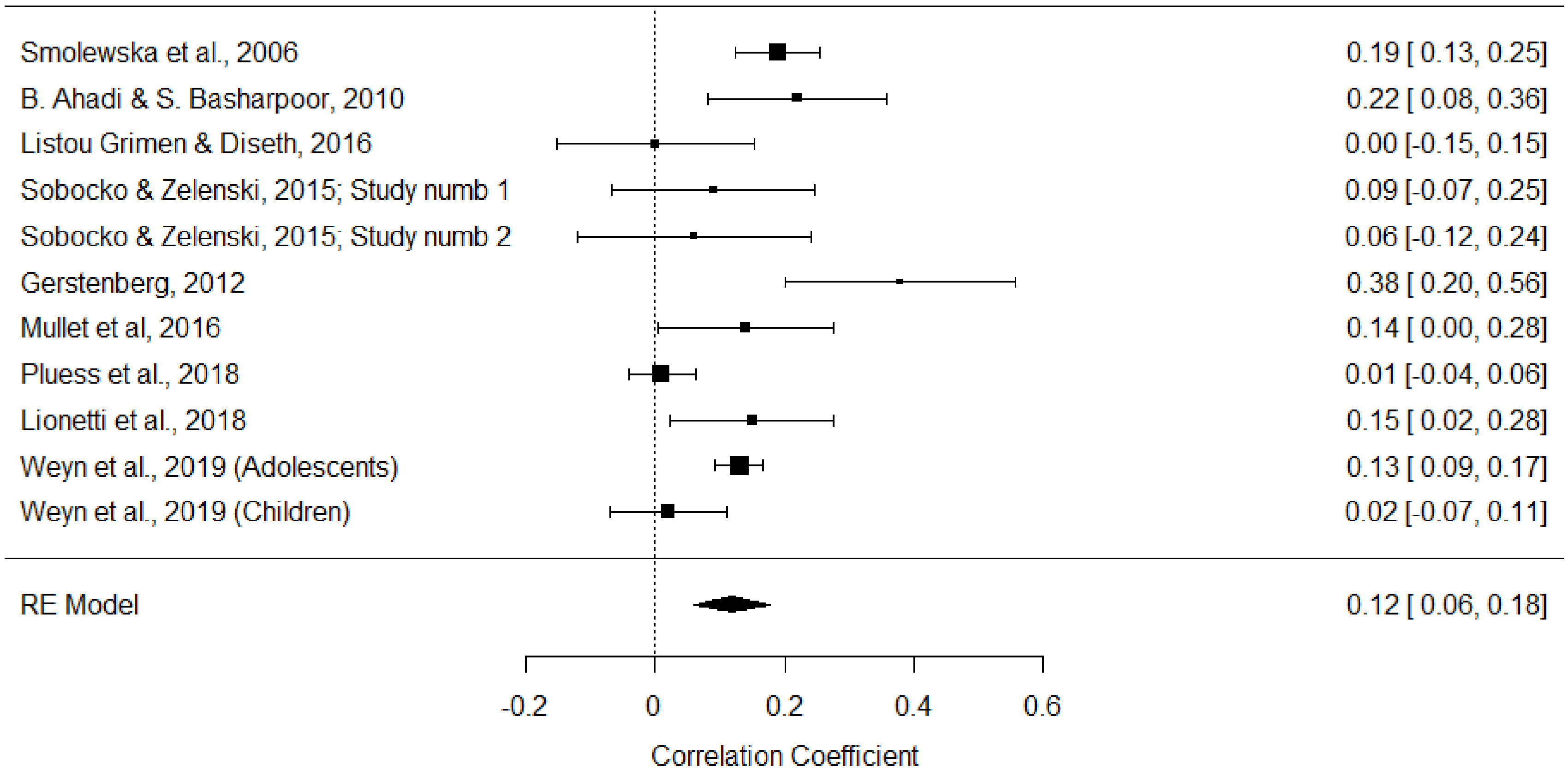

Note. $K = 11$, $N = 6{,}519$

Section B

Figure S5

A Forest Plot of Correlations Between Total HSPS Scale and Introversion

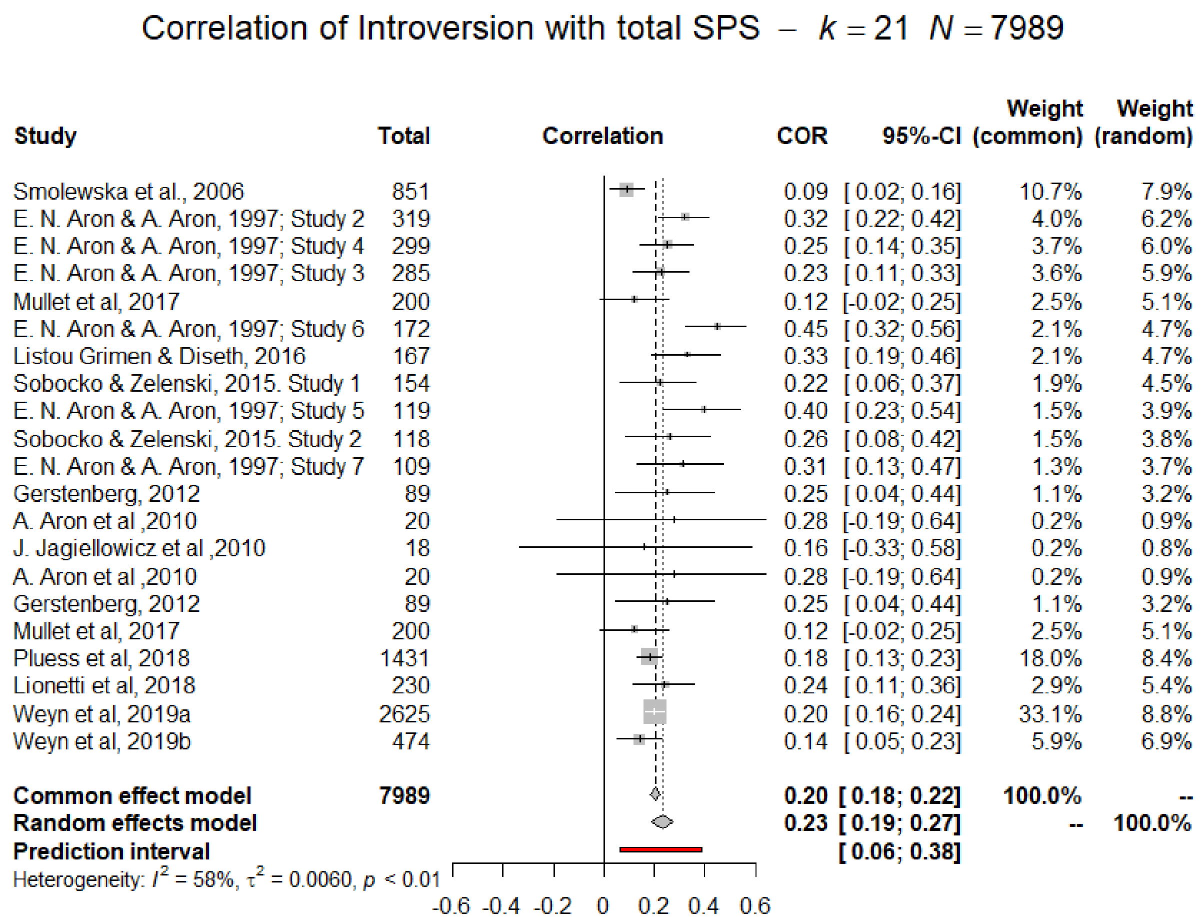

Note. K = 21, total N = 7,989. HSPS = Highly Sensitive Person Scale.

Section C

Additional analysis of three different matrix-reduction techniques

We used an additional three different matrix-reduction techniques. First, we subjected the HSPS and ASP items to a principal components analysis (PCA) with Promax rotation, using *princomp* function in *R* (R Core Team, 2018). We considered loading exceeding .40 to indicate that the item reflects a particular factor. The benefit of PCA is that it uses the same approach to item analysis used to test the dimensionality of HSPS (Smolewska, McCabe, & Woody, 2006) and ASP (Brown et al., 2001)[1]. Second, we subjected these scales to multiple-dimension scaling (MDS) with *cmdscale* function from *stats* package *R* (R Core Team, 2018). The benefit of MDS is that it does not assume a simple structure. That is, each item could be a manifestation of more than one underlying latent cause. This approach is better suited to test SP theory because it assumes that two underlying dimensions create four clusters. Last, we employed network analysis (Dalege et al., 2017) on the HSPS and ASP items, separately and in combination—using several *R* packages (see examples in Dalege et al., 2017). The benefit of the network analysis is that it can provide a more nuanced view of the relationship among the items.

Based on the consistency in results of our matrix-reduction techniques, we constructed sub-scales of HSPS and ASP, computed the correlations among them and with neuroticism, and used all to predict belonging to the HSP-Facebook group, using logistic regression, to test the content validity of all sub-scales.

Results

HSPS

[1] Note that Brown, C., Tollefson, N., Dunn, W., Cromwell, R., & Filion, D. (2001). The adolescent adult sensory profile: Measuring patterns of sensory processing. *American Journal of Occupational Therapy, 55*(1), 75-82. used PCA with a *varimax* rotation, which assumes no correlation among the factors. This assumption is inconsistent with the SP theory. Therefore, we used an oblique-rotation technique.

The original HSPS was claimed to tap a single construct. However, prior work revealed three factors: *Ease of Excitation*, *Low Sensory Threshold*, and *Aesthetic Sensitivity*. Our PCA suggested four factors, three similar, but not identical, to the *Ease of Excitation*, *Low Sensory Threshold,* and *Aesthetic Sensitivity*, and a separate factor, loaded with one item tapping avoiding violent movies and TV (see Table S1).

MDS of the 27 items indicated that two dimensions are sufficient to describe the data, stress = .14. The MDS plot suggests a clear separation of the *Aesthetic Sensitivity* items but an inconclusive division between *Low Sensory Threshold* and *Ease of Excitation*. The plot further indicates that *Low Sensory Threshold* tends to be between *Aesthetic Sensitivity* and *Ease of Excitation* (see Figure S6).

Finally, we subjected these items to network analysis, which suggested four clusters. Like the PCA and the MDS, the network analysis separated the *Aesthetic Sensitivity* items. Moreover, the network analysis suggests separating the *Ease of Excitation* from the *Low Sensory Threshold* items. And similar to MDS, it suggests that *Low Sensory Threshold* items are between *Aesthetic Sensitivity* and *Ease of Excitation*. However, with some similarities to the PCA, the network analysis suggests an additional cluster of four items that appear to tap avoidance (see Figure S7).

ASP

The original ASP was claimed to tap four profiles defined by two axes creating four profiles, each with 15 items. Our PCA suggested 14 factors. Restricting the PCA solution to four components did not recover the proposed profiles (see Table S2). Yet, given that the theory proposed that each profile reflects the manifestation of two axes, PCA may be inappropriate because PCA searches for a simple-structure solution, where each item loads on one, and only one, component.

MDS of the 60 items indicated that two dimensions are almost sufficient to describe the data, stress = .158. However, adding a third dimension improved the stress slightly, .11, but did not change the configuration of the first two dimensions and did not reveal interpretable patterns when exploring the third dimension. Therefore, we proceeded to interpret the two-dimension solution. The results (Figure S8) differentiated the *Sensation Seeking* items and most of the *Low Registration* items but did not differentiate between *Sensory Avoiding* and *Sensory Sensitivity*.

Network analysis of the 60 items revealed five clusters (see Figure S9). A mix of *Sensation Avoiding* and *Sensory Sensitivity* items loaded on two clusters, different from the theory, yet consistent with Brown et al. (2001, p. 78). *Sensation Seeking* items formed one region (community). *Low Registration* items formed two adjacent communities, one of which was composed of five items, of which all contained “I don’t” as part of the question. Thus, our analyses suggest that *Sensation Seeking* and *Low Registration* largely conforms to the original ASP model, but where the latter may have some items with wording effect. Also, *Sensation Avoiding* and *Sensory Sensitivity* are not distinguishable empirically. Because of the partial similarity, with few aberrations, of the MDS and the network analysis with Dunn’s (1997) original theory, we constructed three scales based on the original theory reflecting empirically supported clusters: *Sensation Seeking*, *Low Registration*, and *Sensory Sensitivity* + *Sensation Avoiding*. However, we also constructed one subscale, reflecting negatively worded *Low Registration* items.

HSPS and ASP items combined

To explore the convergence and divergence of the respective factors and profiles of the HSPS and ASP, we also applied the same matrix-reduction techniques to the pool of items taken from both the HSPS and ASP. PCA of all of the HSPS and ASP items was not useful because it

suggested, by the criterion of eigenvalue > 1, 18 factors. Nevertheless, scree test suggested a five-factor solution (see Table S3). However, PCA itself may not be suitable because the ASP theory suggests that two axes are responsible for four profiles.

MDS of the 87 items indicated that two dimensions are sufficient to describe the data, stress = .148. The results differentiated the *Sensation Seeking* items, most of the *Aesthetic Sensitivity* items, and indicated somewhat differentiation of the *Low Registration* items. But, it did not differentiate between *Ease of Excitation*, *Low Sensory Threshold*, *Sensory Sensitivity,* and *Sensory Avoiding* (Figure S10).

Network analysis of the 87 items revealed four clusters (see Figure S11). Most of the HSPS items formed one region (community), except five (from the six) *Aesthetic Sensitivity* items loaded together with the *Sensation Seeking* items. Most of the *Low Registration* items formed one region. And most items of the two low neurological threshold profiles (from ASP), namely, *Sensory Avoiding* and *Sensory Sensitivity*, were not separable and clustered together in one region. Nevertheless, this region is visually separated by *Low Registration* and *Sensation Seeking* items. This finding is consistent with Brown et al. (2001, p. 78), indicating categorization according to Dunn's (1997) Model of Sensory Processing.

Subscales of HSPS and ASP

The results obtained by the PCA, MDS, and network analysis raise the question of identifying the subscales (summary of suggested subscales across all methods presented in Table S4, correlations are presented in Tables S5a-S5b). On the one hand, we considered constructing subscales based on consistent results obtained across all the methods and inclusion (HSPS and ASP items separately or combined). This approach safeguards against an error of commission that identifies subscales that do not exist. On the other hand, merging scales could lead to the

loss of information, or error of omission, neglecting true subscales that may have subtle but true differentiation.

Table S4 summarizes the conclusions suggested by all methods (PCA, MDS, or network analysis) and analyses. The *Low Registration* items are differentiated from all other items in four out of six analyses. Also, the *Sensory Sensitivity* and the *Sensory Avoiding* items are merged and separated from all other items in four out of six analyses. Furthermore, HSPS and ASP items were largely separated, excluding *Aesthetic Sensitivity* (HSPS) and *Sensation Seeking* (ASP) items. Although PCA and MDS suggested separated factors for *Aesthetic Sensitivity* (HSPS) items and *Sensation Seeking* (ASP) items, network analysis formed one region (although not visually mixed). Theoretically, while both subscales relate to awareness and excitement from some stimulus, *Aesthetic Sensitivity* (HSPS) relates specifically to aesthetic stimuli (such as being moved by the arts). *Sensation Seeking* (ASP) relates to actively seeking for all experiences full of sensory-stimulation (such as crowded places, loud music, and bright lights). Experiencing intense sensory stimulation will likely cause HSPs an overarousal and overwhelm them. In contrast, those high on the ASP sensation seeking are likely to seek, rather than avoid, these experiences. Thus, these subscales may not relate to the same sensory tendency.

Another question regards the items of *Ease of Excitation* (HSPS) and *Low-Sensory Thresholds* (HSPS). As shown in Table S4, some of our analyses on HSPS suggested different factors of *Ease of Excitation* and *Low-Sensory Thresholds*. Yet, this distinction disappeared in the MDS of HSPS and network analysis of both HSPS and APS items. The difference between these results is not surprising because these subscales are highly correlated (*rs* = .74 - .78; see Table S5a). Theoretically, while both subscales relate to experiencing stimuli, they do not refer to the same experience. *Ease of Excitation* relates to becoming mentally overwhelmed by external and internal stimuli (overwhelmed by hunger, multiple tasks, and many things happening around).

Low Sensory Threshold relates specifically to the sensory sensitivity to external stimuli that HSPs experience as unpleasant sensory arousal (such as strong lights, loud noise, pain, and others' feelings). Thus, merging these scales could lead to a loss of information.

To answer these division questions, we also used a series of regression analyses, predicting belonging to the Facebook group of HSP, neuroticism, age, and gender. If two similar scales nevertheless yield differential validity, they should be kept as separate subscales.

Validity

To test the differential validity of HSPS and ASP and their respective subscales, we used four variables as criteria: neuroticism, membership in the Facebook group of HSPs (yes/ no), age, and gender. For the last three criteria, we also controlled for neuroticism. For neuroticism and age, we used multiple regression, and for Facebook membership and gender, we used logistic regression. As can be seen in Table S6a, SPS strongly predicts neuroticism, $\beta = .57$, but ASP explains additional variance in neuroticism, $\beta = .16$. Second, it appears that EOE of the SPS and SsSA of the ASP are the best predictors of neuroticism, but all other subscales, except for AES, explain additional variance that is both statistically significant and yet relatively small. These results indicate, first, that although both SPS and ASP are highly correlated with neuroticism, they are not isomorphic with neuroticism as the upper bounds of the confidence intervals are well below unity. Second, these results indicate that ASP may tap some variance in neuroticism not accounted for by HSPS. Third, the subscales of EOE from HSPS and SsSA from ASP that are most similar in the network analysis show here the dominant effect on neuroticism, hinting, perhaps that these subscales are closest to the heart of the construct of sensory sensitivity. Fourth, each scale (HSPS and ASP) have other subscales that may be relevant as they explain additional variance in neuroticism, albeit to a minimal degree.

Next, as can be seen in Table S6b, the logistic regressions indicate a main effect of HSPS on Facebook membership. Specifically, participants in the HSP Facebook group were more likely to be high in HSPS, than were those who were not members in the Facebook group, OR = 4.08 (95% CI: 3.22, 5.28), $p < .001$. Second, it appears that LST of the HSPS and SsSA of the ASP are the best predictors of Facebook membership. At the same time, neuroticism and all other HSPS and ASP subscales explain an additional amount of variance that is both statistically significant and small (especially Se).

Moreover, as seen in Table S6c, HSPS slightly predicts age, $\beta = .18$. And TV of the HSPS is the best predictor of age, $\beta = .20$, but neuroticism and all other subscales explain additional variance. Thus, the higher the participants' age, the more likely they were to report avoiding violent movies and TV.

Furthermore, the logistic regressions presented in Table 6d indicate a main effect of neuroticism on gender (0 = males and 1 = females), OR = 1.14 (95% CI: 1.08, 1.20), $p < .001$, and HSPS on gender, OR = 1.39 (95% CI: 1.25, 1.56), $p < .001$. Second, it appears that EOE and TV of the HSPS and SsSA of the ASP are the best predictors of being a female, while all other HSPS and ASP subscales explain additional variance.

Importantly, in all the analyses (Tables S6b-S6d), there were sensitivity measures that predicted variance in the criterion variables that neuroticism could not explain. This results pattern is another indication the HSPS and ASP are not isomorphic with neuroticism.

Table S1

Loadings of Highly Sensitive Person Scale (HSPS) Items in a Principal Components Analysis with Promax Rotation

Item	EOE	LST	AES	TV
14. Do you get rattled when you have a lot to do in a short amount of time?	.90			
16. Are you annoyed when people try to get you to do too many things at once?	.80			
17. Do you try hard to avoid making mistakes or forgetting things?	.78			
24. Do you make it a high priority to arrange your life to avoid upsetting or overwhelming situations?	.65			
26. When you must compete or be observed while performing a task, do you become so nervous or shaky that you do much worse than you would otherwise?	.61			
23. Do you find it unpleasant to have a lot going on at once?	.58			
21. Do changes in your life shake you up?	.54			
27. When you were a child, did parents or teachers seem to see you as sensitive or shy?	.53			
13. Do you startle easily?	.45			
25. Are you bothered by intense stimuli, like loud noises or chaotic scenes?	.45			
19. Do you become unpleasantly aroused when a lot is going on around you?	.42			
11. Does your nervous system sometimes feel so frazzled that you just have to go off by yourself?				
6. Are you particularly sensitive to the effects of caffeine?		.77		
7. Are you easily overwhelmed by things like bright lights, strong smells, coarse fabrics, or sirens close by?		.75		
1. Are you easily overwhelmed by strong sensory input?		.68		
9. Are you made uncomfortable by loud noises?		.61		
3. Do other people's moods affect you?		.46		
4. Do you tend to be more sensitive to pain?		.46		
5. Do you find yourself needing to withdraw during busy days, into bed or into a darkened room or any place where you can have some privacy and relief from stimulation?		.44		
20. Does being very hungry create a strong reaction in you, disrupting your concentration or mood?				
2. Do you seem to be aware of subtleties in your environment?			.75	
22. Do you notice and enjoy delicate or fine scents, tastes, sounds, works of art?			.70	
10. Are you deeply moved by the arts or music?			.68	
15. When people are uncomfortable in a physical environment do you tend to know what needs to be done to make it more comfortable (like changing the lighting or the seating)?			.66	
8. Do you have a rich, complex inner life?			.62	
12. Are you conscientious?			.41	
18. Do you make a point to avoid violent movies and TV shows?				.65

Figure S6

Multiple Dimension Scaling (MDS) of all Items of the Highly Sensitive Person Scale (HSPS)

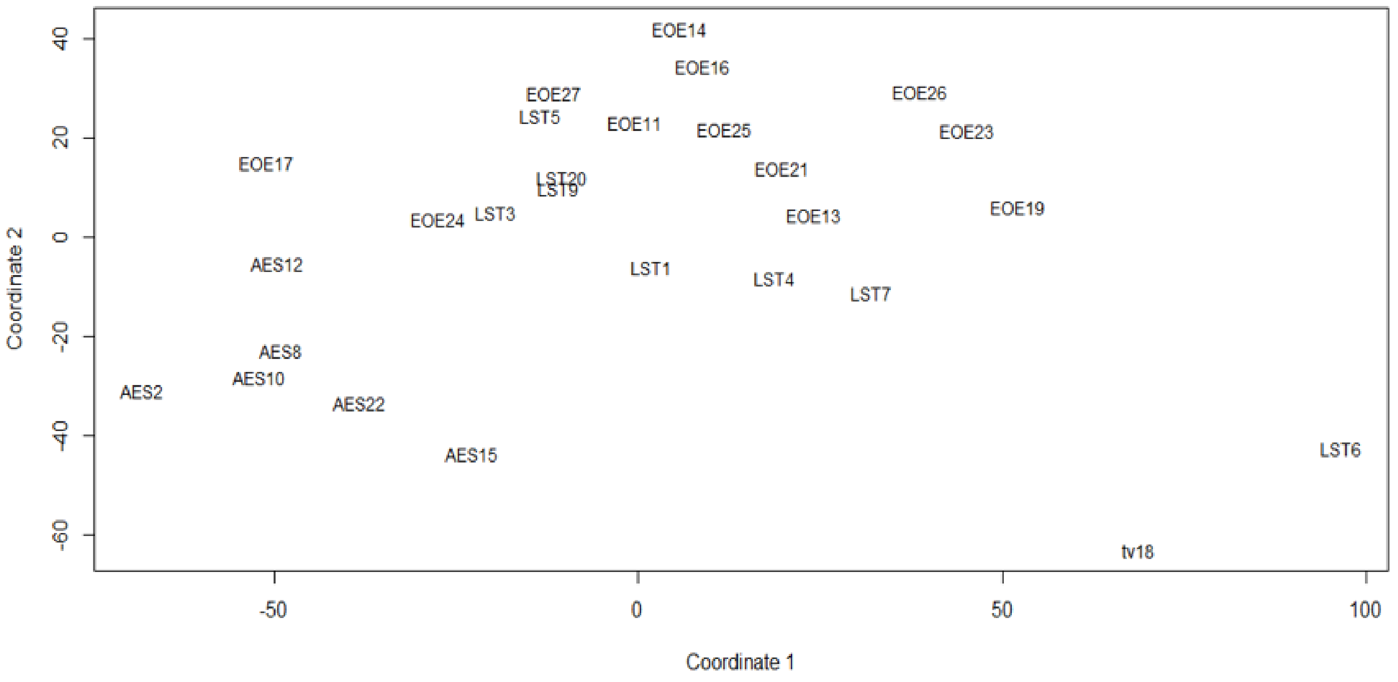

Note. N = 1,240. EOE = Ease of Excitation; AES = Aesthetic Sensitivity; LST = Low Sensory Threshold; TV = avoiding violent movies and TV**.**

Figure S7

Network analysis of all Highly Sensitive Person Scale (HSPS) items

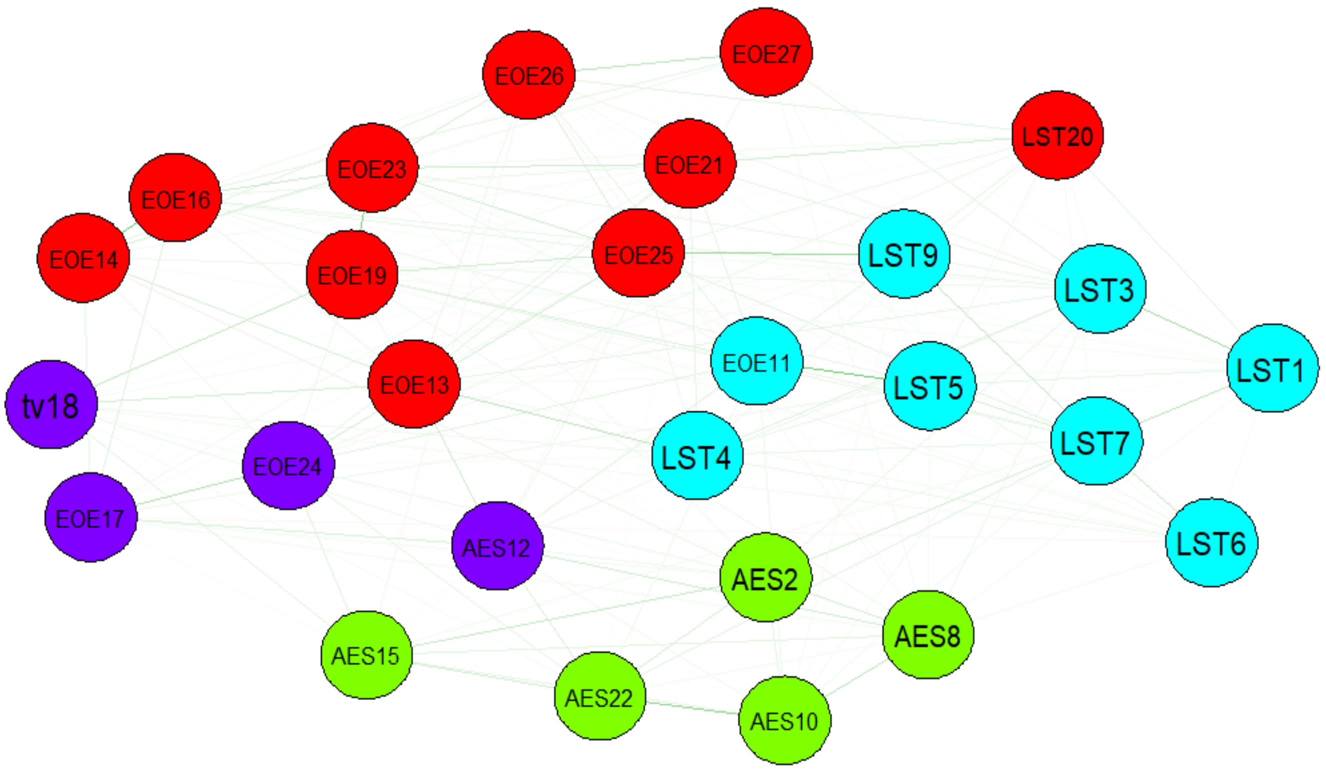

Note. *N* = 1,240. EOE = Ease of Excitation; AES = Aesthetic Sensitivity; LST = Low Sensory Threshold; TV = avoiding violent movies and TV.

Table S2

Loadings of the Adolescent Adult Sensory Profile(ASP) Items in a Principal Components Analysis with Promax Rotation.

Item	SsSA	Se	LR
54. I am distracted if there is a lot of noise around.	.83		
57. I stay away from noisy settings.	.79		
46. I stay away from crowds.	.73		
25. I become bothered when I see lots of movement around me (for example, at a busy mall, parade, carnival).	.72		
26. I limit distractions when I am working (for example, I close the door, or turn off the TV).	.67		
22. I am bothered by unsteady or fast moving visual images in movies or TV.	.67		
60. I find it difficult to work with background noise (for example, fan, radio).	.67		
53. I leave the room when others are watching TV, or I ask them to turn it down.	.64		
56. I use strategies to drown out sound (for example, close the door, cover my ears, wear ear plugs).	.63		
35. I move away when others get too close to me.	.62		
38. I avoid standing in lines or standing close to other people because I don’t like to get too close to others.	.58		
20. I become frustrated when trying to find something in a crowded drawer or messy room.	.56		
24. I choose to shop in smaller stores because I’m overwhelmed in large stores.	.56		
51. I startle easily at unexpected or loud noises (for example, vacuum cleaner, dog barking, telephone ringing).	.54		
52. I have trouble following what people are saying when they talk fast or about unfamiliar topics.	.53		
49. I avoid situations where unexpected things might happen (for example, going to unfamiliar places or being around people I don’t know).	.53		
48. I find it hard to concentrate for the whole time when sitting in a long class or a meeting.	.50		
44. I seem slower than others when trying to follow an activity or task.	.48		
33. I’m uncomfortable wearing certain fabrics (for example, wool, silk, corduroy, tags in clothing).	.48		
16. I become dizzy easily (for example, after bending over, getting up too fast).	.43		
8. I go over to smell fresh flowers when I see them.	.43		
43. I find time to get away from my busy life and spend time by myself.			
59. I have to ask people to repeat things.			
9. I’m afraid of heights.			
1. I leave or move to another section when I smell a strong odor in a store (for example, bath products, candles, perfumes).			
31. I am bothered by the feeling in my mouth when I wake up In the morning.			
45. I don’t get jokes as quickly as others.			

7. I don’t like strong tasting mints or candies (for example, hot/cinnamon or sour candy).		
29. I avoid or wear gloves during activities that will make my hands messy.		
41. It takes me more time than other people to wake up in the morning.		
27. I dislike having my back rubbed.		
10. I enjoy how it feels to move about (for example, dancing, running).	.59	
58. I like to attend events with a lot of music.	.57	
30. I touch others when I’m talking (for example, I put my hand on their shoulder or shake their hands).	.46	
17. I like to go to places that have bright lights and that are colorful.	.45	
28. I like how it feels to get my hair cut.	.45	
19. I like to wear colorful clothing.	.45	
50. I hum, whistle, sing, or make other noises.	.45	
14. I choose to engage in physical activities.	.45	
42. I do things on the spur of the moment (in other words, I do things without making a plan ahead of time).	.42	
32. I like to go barefoot.	.41	
47. I find activities to perform in front of others (for example, music, sports, acting, public speaking, and answering questions in class).		
4. I enjoy being close to people who wear perfume or cologne.		
2. I add spice to my food.		
40. I work on two or more tasks at the same time.		
39. I don’t seem to notice when someone touches my arm or back.		.74
23. I don’t notice when people come into the room.		.70
36. I don’t seem to notice when my face or hands are dirty.		.70
55. I don’t notice when my name is called.		.61
3. I don’t smell things that other people say they smell.		.58
12. I trip or bump into things.		.51
15. I am unsure of footing when walking on stairs (for example, I trip, lose balance, and/or need to hold the rail).		.51
37. I get scrapes or bruises but don’t remember how I got them.		.44
6. Many foods taste bland to me (in other words, food tastes plain or does not have a lot of flavor).		.42
11. I avoid elevators and/or escalators because 1 dislike the movement.		.42
21. I miss the street, building, or room signs when trying to go somewhere new.		
13. I dislike the movement of riding in a car.		
18. I keep the shades down during the day when I am at home.		
34. I don’t like particular food textures (for example, peaches with skin, applesauce, cottage cheese, chunky peanut butter).		
5. I only eat familiar foods.		

Figure S8

Multiple Dimension Scaling (MDS) of all items of *the Adolescent Adult Sensory Profile(ASP)*

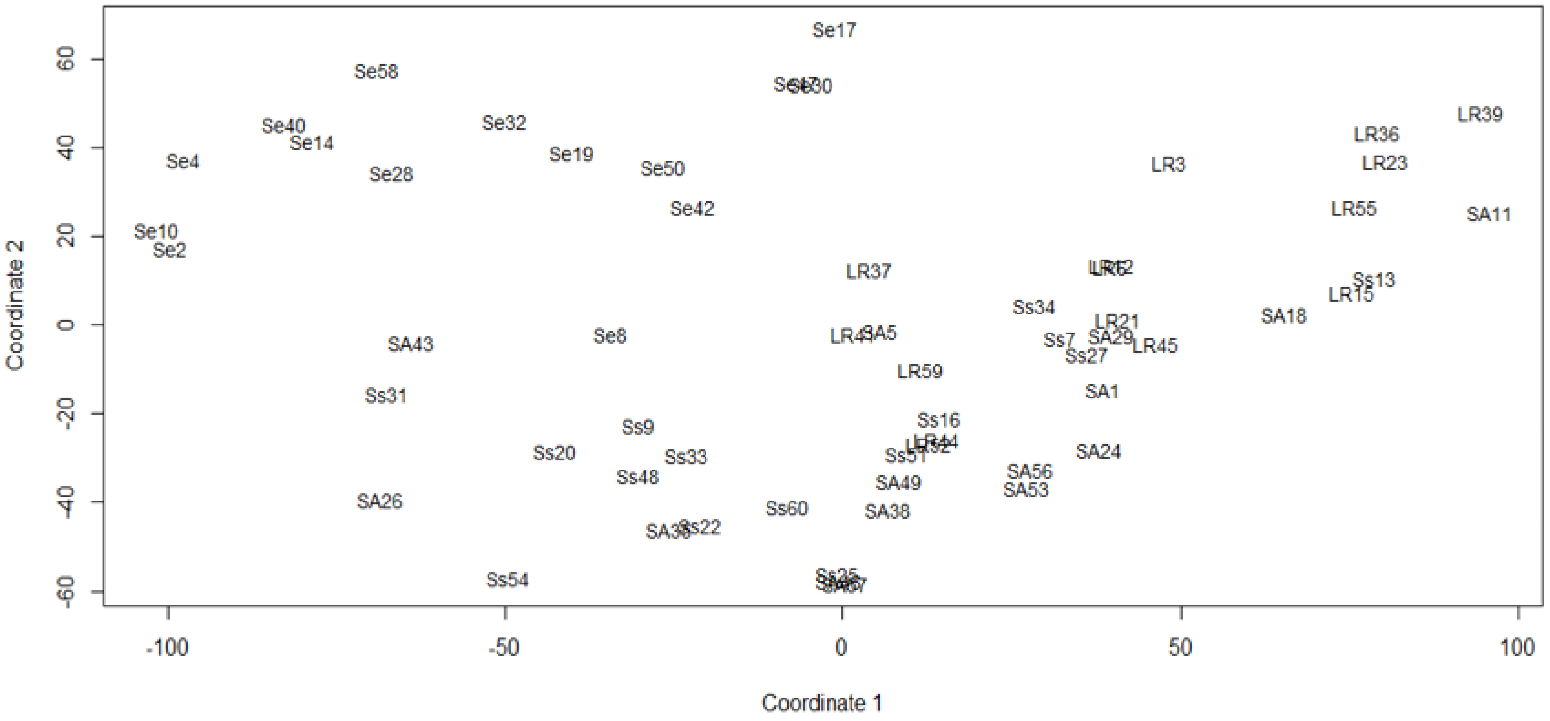

Note. N = 1,240. Ss = Sensory Sensitivity; SA = Sensory Avoiding; Se = Sensation Seeking; LR = Low Registration.

Figure S9

Network analysis of all Adolescent Adult Sensory Profile(ASP) items.

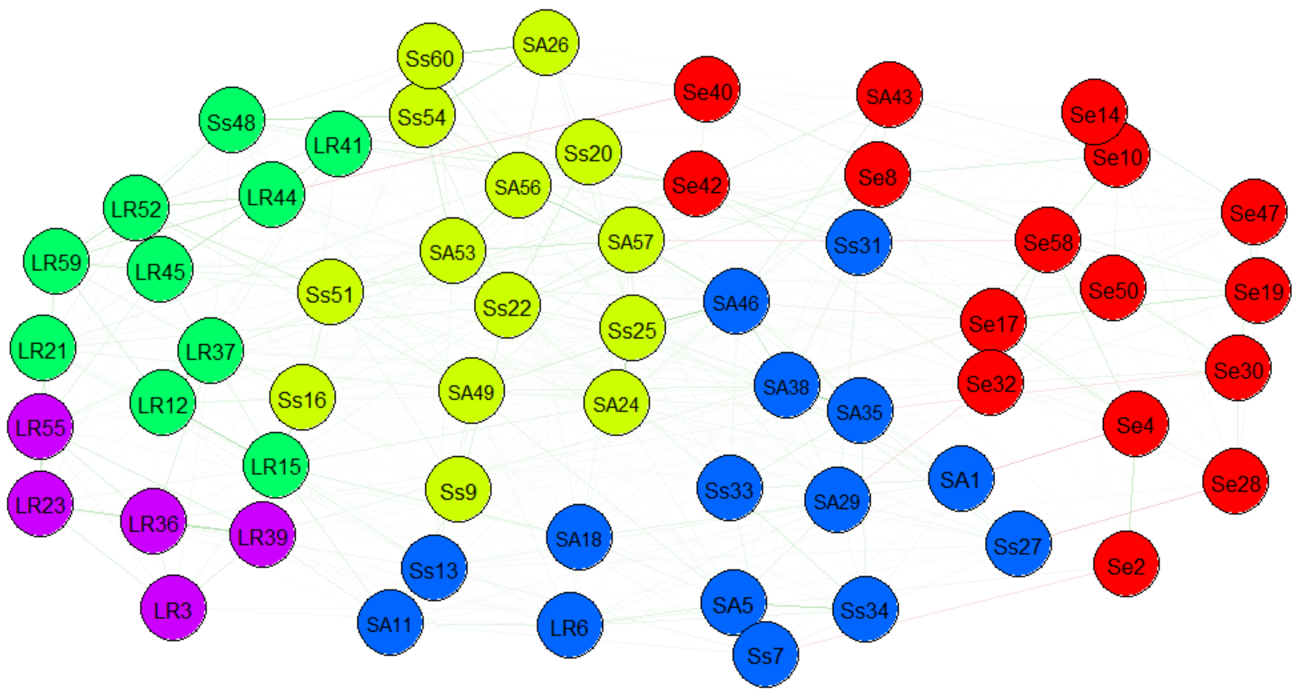

Note. *N* = 1,240. Ss = Sensory Sensitivity; SA = Sensory Avoiding; Se = Sensation Seeking; LR = Low Registration.

Table S3

Loadings of Highly Sensitive Person Scale (HSPS) and Adolescent Adult Sensory Profile(ASP) Items in a Principal Components Analysis with Promax Rotation

Item	F1	F2	F3	F4	F5
SPS14. Do you get rattled when you have a lot to do in a short amount of time?	.81				
SPS16. Are you annoyed when people try to get you to do too many things at once?	.77				
SPS13. Do you startle easily?	.70				
SPS17. Do you try hard to avoid making mistakes or forgetting things?	.69				
SPS25. Are you bothered by intense stimuli, like loud noises or chaotic scenes?	.67				
SPS21. Do changes in your life shake you up?	.66				
SPS26. When you must compete or be observed while performing a task, do you become so nervous or shaky that you do much worse than you would otherwise?	.65				
SPS3. Do other people's moods affect you?	.63				
SPS23. Do you find it unpleasant to have a lot going on at once?	.61				
SPS24. Do you make it a high priority to arrange your life to avoid upsetting or overwhelming situations?	.61				
SPS4. Do you tend to be more sensitive to pain?	.59				
SPS5. Do you find yourself needing to withdraw during busy days, into bed or into a darkened room or any place where you can have some privacy and relief from stimulation?	.58				
SPS11. Does your nervous system sometimes feel so frazzled that you just have to go off by yourself?	.58				
SPS27. When you were a child, did parents or teachers seem to see you as sensitive or shy?	.56				
SPS20. Does being very hungry create a strong reaction in you, disrupting your concentration or mood?	.55				
SPS12. Are you conscientious?	.52				
SPS19. Do you become unpleasantly aroused when a lot is going on around you?	.49				
SPS9. Are you made uncomfortable by loud noises?	.49				
SPS1. Are you easily overwhelmed by strong sensory input?	.45				

SPS7. Are you easily overwhelmed by things like bright lights, strong smells, coarse fabrics, or sirens close by?	.40	
Ss20. I become frustrated when trying to find something in a crowded drawer or messy room.		
SA49. I avoid situations where unexpected things might happen (for example, going to unfamiliar places or being around people I don’t know).		
Se4. I enjoy being close to people who wear perfume or cologne.		
Ss51. I startle easily at unexpected or loud noises (for example, vacuum cleaner, dog barking, telephone ringing).		
Ss31. I am bothered by the feeling in my mouth when I wake up in the morning.		
Ss9. I’m afraid of heights.		
Se10. I enjoy how it feels to move about (for example, dancing, running).		.60
SPS10. Are you deeply moved by the arts or music?		.53
Se58. I like to attend events with a lot of music.		.52
SPS22. Do you notice and enjoy delicate or fine scents, tastes, sounds, works of art?		.51
Se47. I find activities to perform in front of others (for example, music, sports, acting, public speaking, and answering questions in class).		.45
Se30. I touch others when I’m talking (for example, I put my hand on their shoulder or shake their hands).		.45
Se19. I like to wear colorful clothing.		.45
Se50. I hum, whistle, sing, or make other noises.		.44
Se14. I choose to engage in physical activities.		.44
Se32. I like to go barefoot.		.42
Se40. I work on two or more tasks at the same time.		.42
Se8. I go over to smell fresh flowers when I see them.		.41
Se17. I like to go to places that have bright lights and that are colorful.		
SPS15. When people are uncomfortable in a physical environment do you tend to know what needs to be done to make it more comfortable (like changing the lighting or the seating)?		
SPS8. Do you have a rich, complex inner life?		
Se42. I do things on the spur of the moment (in other words, I do things without making a plan ahead of time).		
SPS2. Do you seem to be aware of subtleties in your environment?		
SA43. I find time to get away from my busy life and spend time by myself.		
Se28. I like how it feels to get my hair cut.		

Se2. I add spice to my food.		
LR59. I have to ask people to repeat things.	.60	
LR55. I don’t notice when my name is called.	.58	
LR23. I don’t notice when people come into the room.	.56	
LR39. I don’t seem to notice when someone touches my arm or back.	.52	
LR52. I have trouble following what people are saying when they talk fast or about unfamiliar topics.	.50	
LR12. I trip or bump into things.	.49	
LR36. I don’t seem to notice when my face or hands are dirty.	.47	
LR44. I seem slower than others when trying to follow an activity or task.	.47	
LR37. I get scrapes or bruises but donâ€™t remember how I got them.	.44	
LR45. I don’t get jokes as quickly as others.	.43	
Ss48. I find it hard to concentrate for the whole time when sitting in a long class or a meeting.	.41	
LR3. I don’t smell things that other people say they smell.		
LR21. I miss the street, building, or room signs when trying to go somewhere new.		
LR15. I am unsure of footing when walking on stairs (for example, I trip, lose balance, and/or need to hold the rail).		
LR41. It takes me more time than other people to wake up in the morning.		
Ss16. I become dizzy easily (for example, after bending over, getting up too fast).		
SA57. I stay away from noisy settings.		.68
SA46. I stay away from crowds.		.64
SA53. I leave the room when others are watching TV, or I ask them to turn it down.		.58
Ss25. I become bothered when I see lots of movement around me (for example, at a busy mall, parade, carnival).		.56
SA56. I use strategies to drown out sound (for example, close the door, cover my ears, wear ear plugs).		.53
SA24. I choose to shop in smaller stores because I’m overwhelmed in large stores.		.52
Ss54. I am distracted if there is a lot of noise around.		.48
SA1. I leave or move to another section when I smell a strong odor in a store (for example, bath products, candles, perfumes).		.44
SA38. I avoid standing in lines or standing close to other people because I don’t like to get too close to others.		.43

Ss60. I find it difficult to work with background noise (for example, fan, radio).				.43	
Ss22. I am bothered by unsteady or fast moving visual images in movies or TV.				.42	
SA26. I limit distractions when I am working (for example, I close the door, or turn off the TV).					
Ss33. I'm uncomfortable wearing certain fabrics (for example, wool, silk, corduroy, tags in clothing),					
SA35. I move away when others get too close to me.					
SPS18. Do you make a point to avoid violent movies and TV shows?					
SPS6. Are you particularly sensitive to the effects of caffeine?					
SA11. I avoid elevators and/or escalators because 1 dislike the movement.					.55
SA5. I only eat familiar foods.					.49
Ss34. I don't like particular food textures (for example, peaches with skin, applesauce, cottage cheese, chunky peanut butter).					.46
LR6. Many foods taste bland to me (in other words, food tastes plain or does not have a lot of flavor).					.40
Ss13. I dislike the movement of riding in a car.					
Ss27. I dislike having my back rubbed.					
SA29. I avoid or wear gloves during activities that will make my hands messy.					
Ss7. I don't like strong tasting mints or candies (for example, hot/cinnamon or sour candy).					
SA18. I keep the shades down during the day when I am at home.					

Note. *N* = 1,240. SPS = Highly sensitive person scale; Ss = Sensory Sensitivity; SA = Sensory Avoiding; Se = Sensation Seeking; LR = Low Registration.

Figure S10

Multiple Dimensional Scaling (MDS) of all items of Highly Sensitive Person Scale (HSPS) and the Adolescent Adult Sensory Profile(ASP)

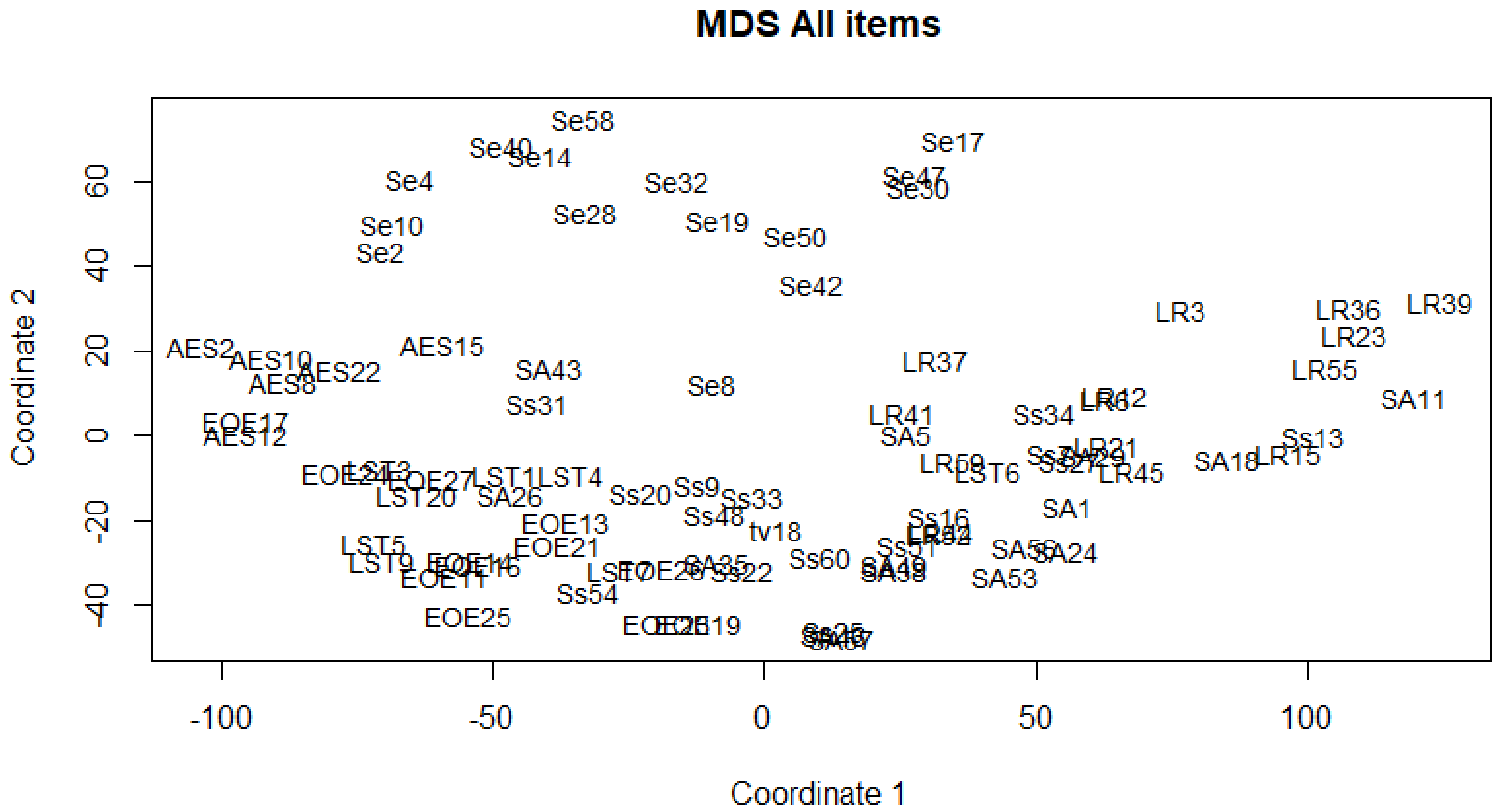

Note. *N* = 1,240. EOE = Ease of Excitation; AES = Aesthetic Sensitivity; LST = Low Sensory Threshold; TV = avoiding violent movies and TV; Ss = Sensory Sensitivity; SA = Sensory Avoiding; Se = Sensation Seeking; LR = Low Registration.

Figure S11

Network analysis of all items of Highly Sensitive Person Scale (HSPS) and the Adolescent Adult Sensory Profile(ASP)

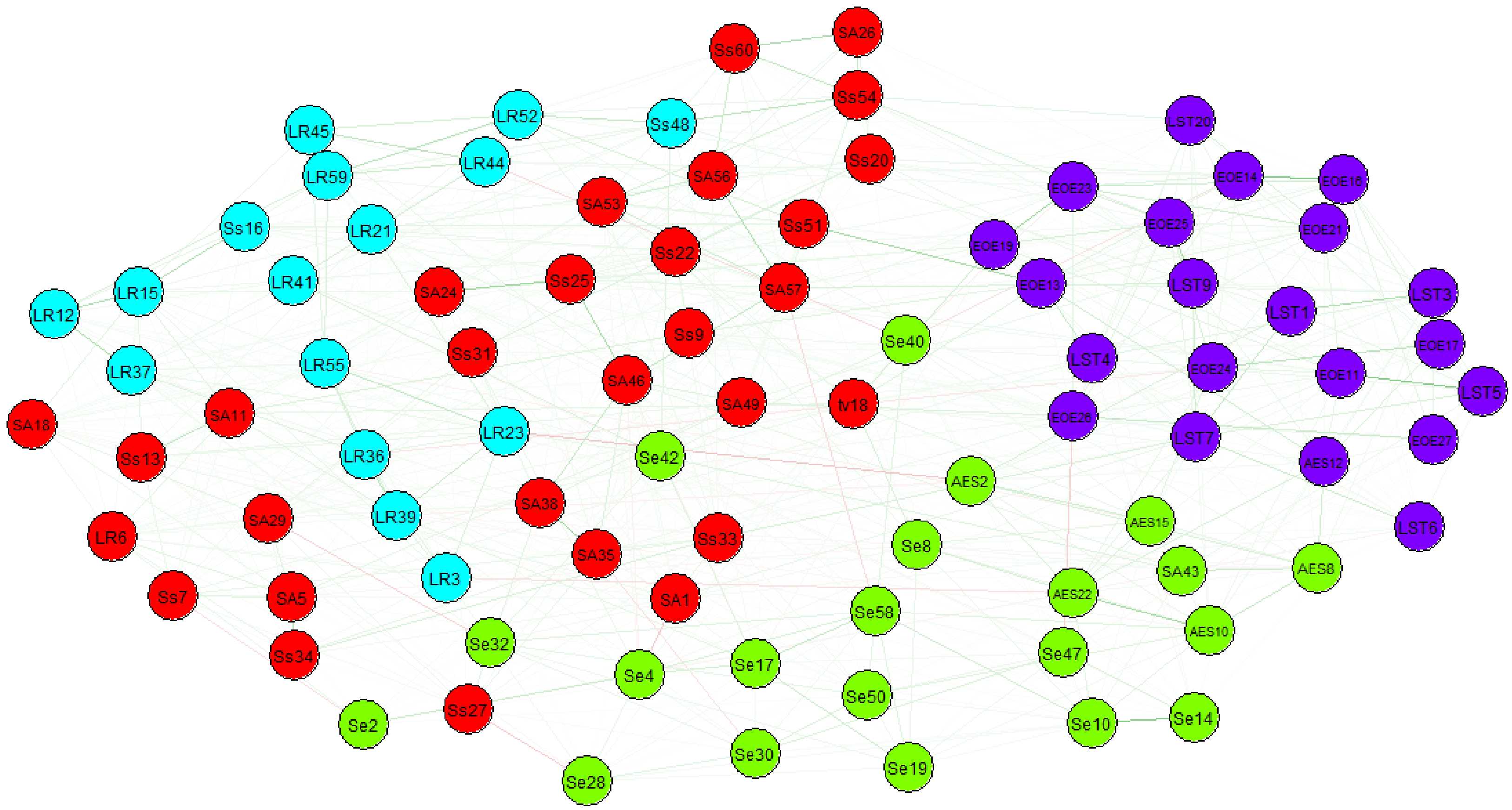

Note. *N* = 1,240. EOE = Ease of Excitation; AES = Aesthetic Sensitivity; LST = Low Sensory Threshold; TV = avoiding violent movies and TV; Ss = Sensory Sensitivity; SA = Sensory Avoiding; Se = Sensation Seeking; LR = Low Registration.

Table S4

Summary of conclusions suggested by all methods (PCA, MDS, and network analysis)

**Matrix-reduction techniques:**	**HSPS**<br>27 items	**ASP**<br>60 items	**HSPS + ASP**<br>87 items
Principal Components Analysis (PCA)	4 factors:<br>**AES**<br>**EOE**<br>**LST**<br>**1 item:** about TV	14 factors<br><br>Restricting (3):<br>**Ss + SA** (21 items)<br>**LR** (10 items)<br>**Se** (9 items)	18 factors<br><br>Scree test (5):<br>**Ss + SA** (21 items)<br>**Ss + SA** (4 items about food issues)<br>**LR** (21 items)<br>**Se** (21 items)<br>**SPS** (21 items)
Multiple-Dimension Scaling (MDS)	3 regions:<br>**AES**<br>**EOE + LST**<br>**2 items:** TV18, LST6	3 regions:<br>**Ss + SA**<br>**LR**<br>**Se**	4 regions:<br>**LR (HSPS)**<br>**Se (ASP)**<br>**AES (HSPS)**<br>**All other**
Network analysis	4 communities:<br>**AES**<br>**EOE**<br>**LST**<br>**4 items:** about avoidance (TV18, EOE17, EOE24, AES12)	5 communities:<br>**Ss + SA**<br>**SA + Ss**<br>**LR**<br>**LR** (5 “I don’t” items)<br>**Se**	4 communities:<br>**Ss + SA (ASP)**<br>**LR (ASP)**<br>**Se (ASP) + AES (HSPS)**<br>**EOE + LST (HSPS)**

Note. N = 1,240. ***AES*** *= Aesthetic Sensitivity;* ***EOE*** *= Ease of Excitation;* ***LST*** *= Low Sensory Threshold; TV = one item of avoiding violent TV;* ***SP: Ss + SA*** *= Sensory Sensitivity and Sensory Avoiding;* ***Se*** *= Sensation Seeking;* ***LR*** *= Low Registration.*

Table S5a

Means, Standard Deviations, and Correlations of Highly Sensitive Person Scale (HSPS) sub-scales using PCA, MDS and Network analysis.

Variable	*M*	*SD*	1	2	3	4	5	6	7	8	9	10	11	12	13	14	15
1. HSPS	5.98	1.69	(.93)														
2. PCA.EOE	5.82	2.04	.93	(.90)													
3. PCA.LST	5.64	1.97	.89	.74	(.81)												
4. PCA.AES	6.87	1.70	.69	.48	.53	(.76)											
5. MDS.EOE	5.84	2.05	.94	**.99**	.76	.49	(.88)										
6. MDS. LST	5.72	1.93	.90	.76	**.99**	.53	.77	(.81)									
7. MDS. AES	6.87	1.70	.69	.48	.53	**1.00**	.49	.53	(.76)								
8. PCA.MDS.TV	4.40	3.59	.49	.40	.36	.32	.40	.36	.32								
9. NET.EOE	5.66	2.12	.92	**.98**	.76	.45	**.98**	.79	.45	.39	(.89)						
10. NET. LST	5.69	2.00	.91	.77	**.99**	.54	.80	**.97**	.54	.37	.78	(.84)					
11. NET. AES	6.81	1.76	.64	.42	.49	**.98**	.43	.49	**.98**	.29	.39	.50	(.74)				
12. NET.SPS.AVOID.TV	6.33	1.97	.78	.73	.56	.62	.74	.58	.62	**.69**	.65	.59	.52	(.61)			
13. Age	31.10	12.52	.13	.08	.12	.12	.09	.10	.12	.21	.08	.13	.10	.13			
14. Gender	1.67	0.47	.26	.25	.19	.13	.26	.19	.13	.30	.26	.20	.11	.26	.07		
15. Neuroticism	4.85	2.72	.68	.70	.58	.35	.71	.58	.35	.25	.70	.61	.29	.48	.08	.19	(.84)
16. HSPs' Facebook group	0.15	0.36	.56	.48	.51	.42	.49	.51	.42	.34	.48	.53	.39	.42	.24	.09	.42

Note. N = 1,240. HSPS = Highly sensitive person scale; PCA.EOE = PCA sub-scale of *Ease of Excitation*; PCA.AES = PCA sub-scale of *Aesthetic Sensitivity*; PCA.LST = PCA sub-scale of *Low Sensory Threshold*; MDS.EOE = MDS sub-scale of *Ease of Excitation*; MDS.AES = MDS sub-scale of *Aesthetic Sensitivity*; MDS.LST = MDS sub-scale of *Low Sensory Threshold*; PCA.MDS.TV = PCA & MDS sub-scale that include the following SPS item: *"18. Do you make a point to avoid violent movies and TV shows?"*; NET.EOE = Network analysis sub-scale of Ease of Excitation; NET.AES = Network analysis sub-scale of Aesthetic Sensitivity; NET.LST = Network analysis sub-scale of Low Sensory Threshold; NET.SPS.AVOID.TV = Network analysis sub-scale that includes the following SPS items: *"12. Are you conscientious?"* (AES by PCA and MDS), *"17. Do you try hard to avoid making mistakes or forgetting things?"* (EOE by PCA and MDS), *"24. Do you make it a high priority to arrange your life to avoid upsetting or overwhelming situations?"* (EOE by PCA and MDS), *"18. Do you make a point to avoid violent movies and TV shows?"*; HSPs' Facebook group = membership in HSPs' Facebook group. Values in table are standardized regression coefficient, figures in parentheses are 95% confidence intervals. Figures in **bold** are correlations between similar scales construed based on PCA, MDS, and network analysis. When $|r| > .06$, $p < .05$.

Table S5b

Means, Standard Deviations, and Correlations of the Adolescent Adult Sensory Profile(ASP) sub-scales using PCA, MDS and Network analysis.

Variable	*M*	*SD*	1	2	3	4	5	6	7	8	9	10	11	12	13	14	15
1. ASP	4.03	1.12	(.89)														
2. PCA.SsSA	4.36	1.91	.89	(.91)													
3. PCA.LR	2.29	1.46	.67	.43	(.77)												
4. PCA.Se	4.95	1.55	.27	-.03	.15	(.68)											
5. MDS.SsSA	4.24	1.66	.92	**.98**	.46	-.03	(.90)										
6. MDS.LR	2.51	1.37	.75	.53	**.93**	.07	.57	(.83)									
7. MDS.Se	5.17	1.38	.23	-.10	.12	**.95**	-.09	.04	(.71)								
8. Net.SsSA	3.56	1.85	.82	**.73**	.69	.11	**.76**	.72	.05	(.81)							
9. Net.SASs	3.51	1.55	.78	**.75**	.49	-.15	**.81**	.68	-.18	.54	(.79)						
10. Net.LR	4.36	2.00	.85	.96	**.40**	-.05	.95	**.50**	-.10	.65	.69	(.88)					
11. Net.LR.Neg	1.90	1.58	.46	.22	**.84**	.16	.24	**.76**	.13	.45	.27	.20	(.70)				
12. Net.Se	5.18	1.31	.31	.00	.14	**.94**	.01	.06	**.97**	.10	-.12	-.02	.13	(.71)			
13. Age	31.10	12.52	.01	.08	-.03	-.09	.07	-.03	-.11	-.04	.02	.09	-.04	-.06			
14. Gender	1.67	0.47	.15	.18	.04	.01	.18	.03	.00	.14	.11	.18	-.09	.04	.07		
15. Neuroticism	4.85	2.72	.52	.60	.23	-.08	.61	.30	-.12	.47	.44	.61	.05	-.06	.08	.19	(.84)
16. HSPs' Facebook group	0.15	0.36	.36	.49	.04	-.09	.47	.11	-.15	.25	.33	.47	-.06	-.06	.24	.09	.42

Note. N = 1,240. ASP = Adolescent Adult sensory profiles; PCA.SsSA = PCA sub-scale of *Sensory Sensitivity* and *Sensory Avoiding*; PCA.LR = PCA sub-scale of *Low Registration*; PCA.Se = PCA sub-scale of *Sensation Seeking*; MDS.SsSA = MDS sub-scale of *Sensory Sensitivity* and *Sensory Avoiding*; MDS.LR = MDS sub-scale of *Low Registration*; MDS.Se = MDS sub-scale of *Sensation Seeking*; Net.SsSA = Network analysis sub-scale including mostly items of *Sensory Sensitivity* and also *Sensory Avoiding* items; Net.SASs = Network analysis sub-scale including mostly items of *Sensory Avoiding* and also S*ensory Sensitivity* items; Net.LR = Network analysis sub-scale including most of the *Low Registration* items; Net.LR.Neg = Network analysis sub-scale including negative items of *Low Registration*; Net.Se = Network analysis sub-scale of *Sensation Seeking*; HSPs' Facebook group = membership in HSPs' Facebook group. Values in table are standardized regression coefficient, figures in parentheses are 95% confidence intervals.

Figures in **bold** are correlations between similar scales construed based on PCA, MDS, and network analysis. When |*r*| > .06, *p* < .05.

Table S6a

Neuroticism Regressed on HSPS and ASP (Model 1), HSPS Subscales (Model 2), and ASP Profiles (Model 3).

	Model 1	Model 2	Model 3
HSPS	.57*** (.53, .62)		
ASP	.16*** (.12, .20)		
EOE		.66*** (.60, .71)	
AES		-.01 (-.05, .03)	
LST		.10*** (.04, .15)	
TV		-.04* (-.08, -.01)	
SsSA			.63*** (.59, .68)
Se			-.06** (-.10, -.02)
LR			-.05** (-.10, -.01)

Note. *N* = 1,240. HSPS = Highly sensitive person scale; ASP = Adolescent Adult sensory profiles; EOE = Ease of Excitation; AES = Aesthetic Sensitivity; LST = Low Sensory Threshold; TV = avoiding violent movies and TV; SsSA = Sensory Sensitivity and Sensory Avoiding; Se = Sensation Seeking; LR = Low Registration. Values in table are standardized regression coefficient, figures in parentheses are 95% confidence intervals.

p* < .05; ** *p* < .01; * *p* < .001

Table S6b

Membership in HSPs' Facebook Group (yes/no) Regressed (Logistic Regression) on HSPS and ASP (Model 1), ASP (Model 2), HSPS Subscales (Model 3), and ASP Profiles (Model 4) Controlled for Neuroticism

	Model 1	Model 2	Model 3	Model 4
Neuroticism	1.16** (1.04, 1.30)	1.60*** (1.46, 1.76)	1.23*** (1.09, 1.38)	1.40*** (1.27, 1.55)
HSPS	4.08*** (3.22, 5.28)			
ASP	0.88 (0.69, 1.13)	1.79*** (1.48, 2.18)		
EOE			1.34** (1.09, 1.65)	
AES			1.52*** (1.26, 1.85)	
LST			1.71*** (1.39, 2.12)	
TV			1.10** (1.03, 1.18)	
SsSA				2.56*** (2.15, 3.08)
Se				0.85* (0.74, 0.98)
LR				0.65*** (0.55, 0.76)

Note. N = 1, 240. HSPS = Highly sensitive person scale; ASP = Adolescent Adult sensory profiles; EOE = Ease of Excitation; AES = Aesthetic Sensitivity; LST = Low Sensory Threshold; TV = avoiding violent movies and TV; SsSA = Sensory Sensitivity and Sensory Avoiding; Se = Sensation Seeking; LR = Low Registration. Values in table are odds ratios, figures in parentheses are 95% confidence intervals.

*p < .05; ** p < .01; *** p < .001.

Table S6c

Age Regressed on HSPS and ASP (Model 1), HSPS Subscales (Model 2), and ASP Profiles (Model 3). Regressions Controlled for Neuroticism

	Model 1	Model 2	Model 3
Neuroticism	.01 (-.05, .08)	.05 (-.02, .11)	.05 (-.01, .10)
HSPS	.18*** (.11, .25)		
ASP	-.11*** (-.17, -.05)		
EOE		-.08 (-.17, .002)	
AES		.05 (-.001, .11)	
LST		.04 (-.04, .11)	
TV		.20*** (.15, .26)	
SsSA			.09** (.02, .15)
Se			-.09*** (-.14, -.04)
LR			-.09** (-.14, -.03)

Note. *N* = 1,231. HSPS = Highly sensitive person scale; ASP = Adolescent Adult sensory profiles; EOE = Ease of Excitation; AES = Aesthetic Sensitivity; LST = Low Sensory Threshold; TV = avoiding violent movies and TV; SsSA = Sensory Sensitivity and Sensory Avoiding; Se = Sensation Seeking; LR = Low Registration. Values in table are standardized regression coefficient, figures in parentheses are 95% confidence intervals.

p* < .05; ** *p* < .01; * *p* < .001.

Table S6d

Gender Regressed (Logistic Regression) on ASP (Model 1), ASP and HSPS (Model 2), HSPS Subscales (Model 3), and ASP Profiles (Model 4). Regressions Controlled for Neuroticism

	Model 1	Model 2	Model 3	Model 4
Neuroticism	1.14^{***} (1.08, 1.20)	1.04 (0.97, 1.10)	1.04 (0.97, 1.11)	1.11^{***} (1.05, 1.17)
ASP	1.14^{*} (1.01, 1.30)	0.95 (0.82, 1.10)		
HSPS		1.39^{***}(1.25, 1.56)		
EOE			1.23^{***} (1.10, 1.38)	
AES			0.98 (0.90, 1.07)	
LST			0.95 (0.86, 1.06)	
TV			1.17^{***} (1.13, 1.22)	
SsSA				1.27^{***} (1.14, 1.42)
Se				1.05 (0.96, 1.15)
LR				0.83^{**} (0.74, 0.93)

Note. *N* = 1, 232. Gender is coded 0 = males and 1 = females. HSPS = Highly sensitive person scale; ASP = Adolescent Adult sensory profiles; EOE = Ease of Excitation; AES = Aesthetic Sensitivity; LST = Low Sensory Threshold; TV = avoiding violent movies and TV; SsSA = Sensory Sensitivity and Sensory Avoiding; Se = Sensation Seeking; LR = Low Registration. Values in table are odds ratios, figures in parentheses are 95% confidence intervals. *$p < .05$; ** $p < .01$; *** $p < .001$.

References

Brown, C., Tollefson, N., Dunn, W., Cromwell, R., & Filion, D. (2001). The adult sensory profile: Measuring patterns of sensory processing. *American Journal of Occupational Therapy, 55*(1), 75-82.

Dalege, J., Borsboom, D., van Harreveld, F., & van der Maas, H. L. J. (2017). Network Analysis on Attitudes. *Social Psychological and Personality Science, 8*(5), 528-537. doi:10.1177/1948550617709827

Dunn, W. (1997). The Impact of Sensory Processing Abilities on the Daily Lives of Young Children and Their Families: A Conceptual Model. *Infants & Young Children, 9*(4), 23-35.

R Core Team. (2018). R: A language and environment for statistical computing. R Foundation for Statistical Computing. Vienna, Austria. Retrieved from http://www.R-project.org/

Smolewska, K. A., McCabe, S. B., & Woody, E. Z. (2006). A psychometric evaluation of the Highly Sensitive Person Scale: The components of sensory-processing sensitivity and their relation to the BIS/BAS and "Big Five". *Personality and Individual Differences, 40*(6), 1269-1279.

Section D

Table S7

Neuroticism Regressed on HSPS' correlations with Family support (Model 1), Attachment Anxiety (Model 2), and Avoidant Attachment (Model 3)

	**Neuroticism**								
	Model 1			Model 2			Model 3		
Predictors	*Estimates*	*CI*	*p*	*Estimates*	*CI*	*p*	*Estimates*	*CI*	*p*
(Intercept)	0.00	-0.08 – 0.08	0.966	0.00	-0.08 – 0.08	0.956	-0.00	-0.08 – 0.08	0.917
Family support	-0.08	-0.16 – 0.00	0.063						
HSPS	0.48	0.39 – 0.56	**<0.001**	0.39	0.30 – 0.47	**<0.001**	0.52	0.44 – 0.60	**<0.001**
Family support * HSPS	0.03	-0.04 – 0.11	0.382						
Attachment Anxiety				0.27	0.19 – 0.35	**<0.001**			
Attachment Anxiety * HSPS				0.01	-0.06 – 0.08	0.753			
Avoidant Attachment							0.08	-0.00 – 0.16	0.054
Avoidant Attachment * HSPS							-0.08	-0.16 – 0.00	0.059
Observations	445			462			462		
R^2 / R^2 adjusted	0.232 / 0.227			0.312 / 0.307			0.261 / 0.256		

Section E

Figure S12

Sample without HSPs Facebook members: Structural Consistency of the Highly Sensitive Person Scale (HSPS) and the Adolescent Adult Sensory Profile(ASP)

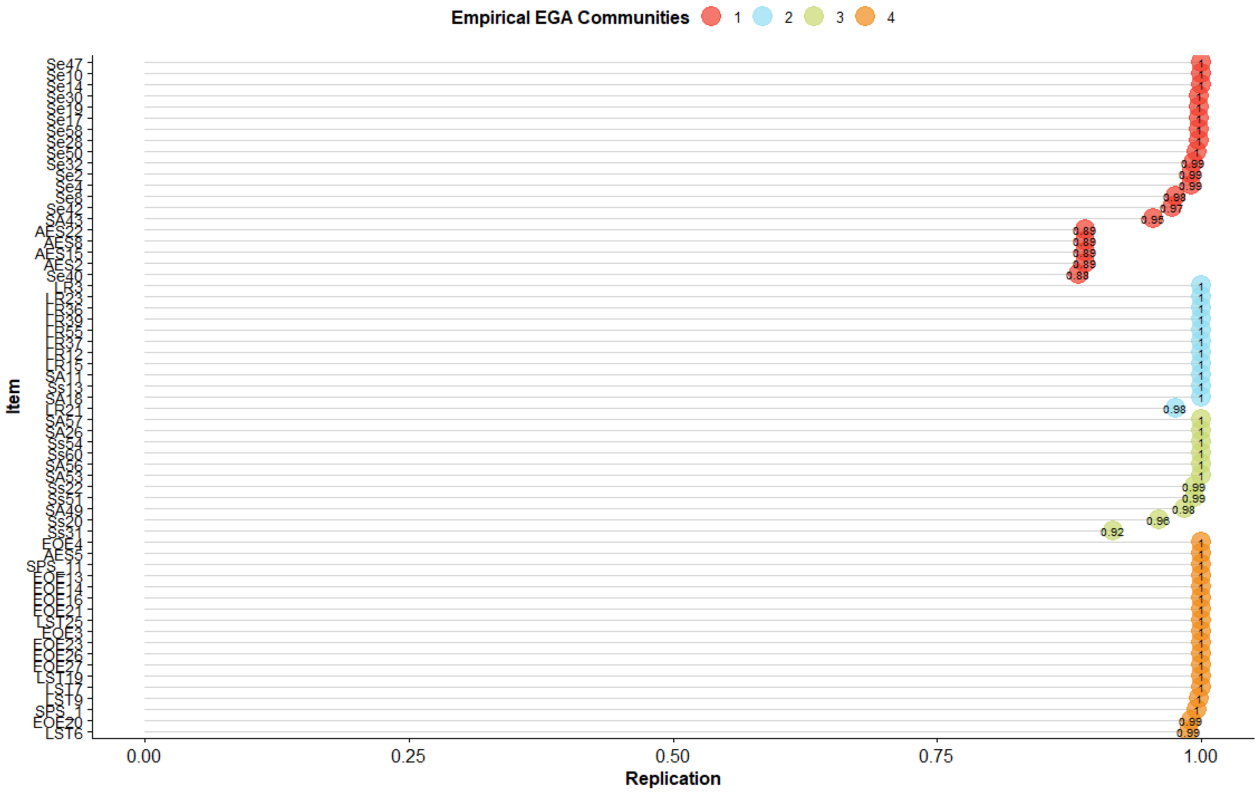

Note. N = 1,515. SPS = items that previous studies did not assign to any subscale of the Highly sensitive person scale; EOE = Ease of Excitation; AES = Aesthetic Sensitivity; LST = Low Sensory Threshold; Ss = Sensory Sensitivity; SA = Sensory Avoiding; Se = Sensation Seeking; LR = Low Registration.

Figure S13

Sample without HSPs Facebook members: Network Plot of the Highly Sensitive Person Scale (HSPS) and the Adolescent Adult Sensory Profile(ASP)

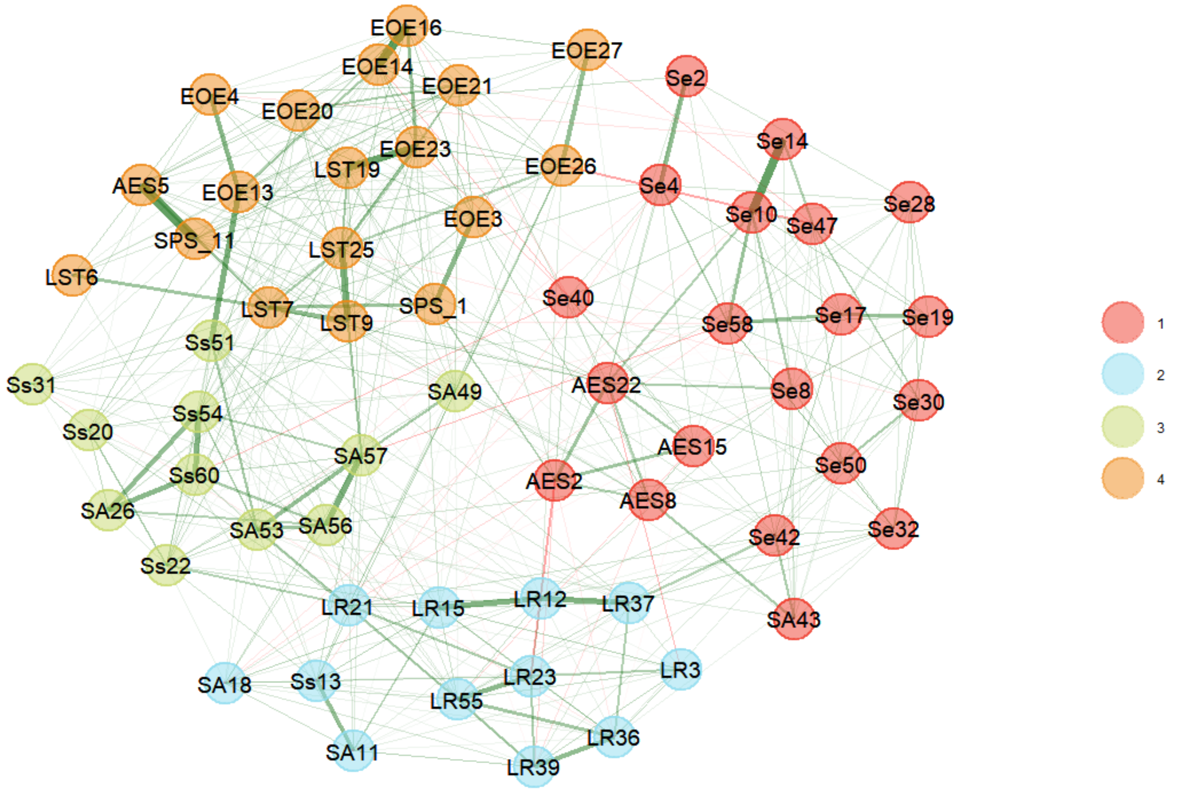

Note. *N* = 1,515. SPS = items that previous studies did not assign to any subscale of the Highly sensitive person scale; EOE = Ease of Excitation; AES = Aesthetic Sensitivity; LST = Low Sensory Threshold; Ss = Sensory Sensitivity; SA = Sensory Avoiding; Se = Sensation Seeking; LR = Low Registration.

Figure S14

Sample of HSPs Facebook members: Structural Consistency of the Highly Sensitive Person Scale (HSPS) and the Adolescent Adult Sensory Profile(ASP)

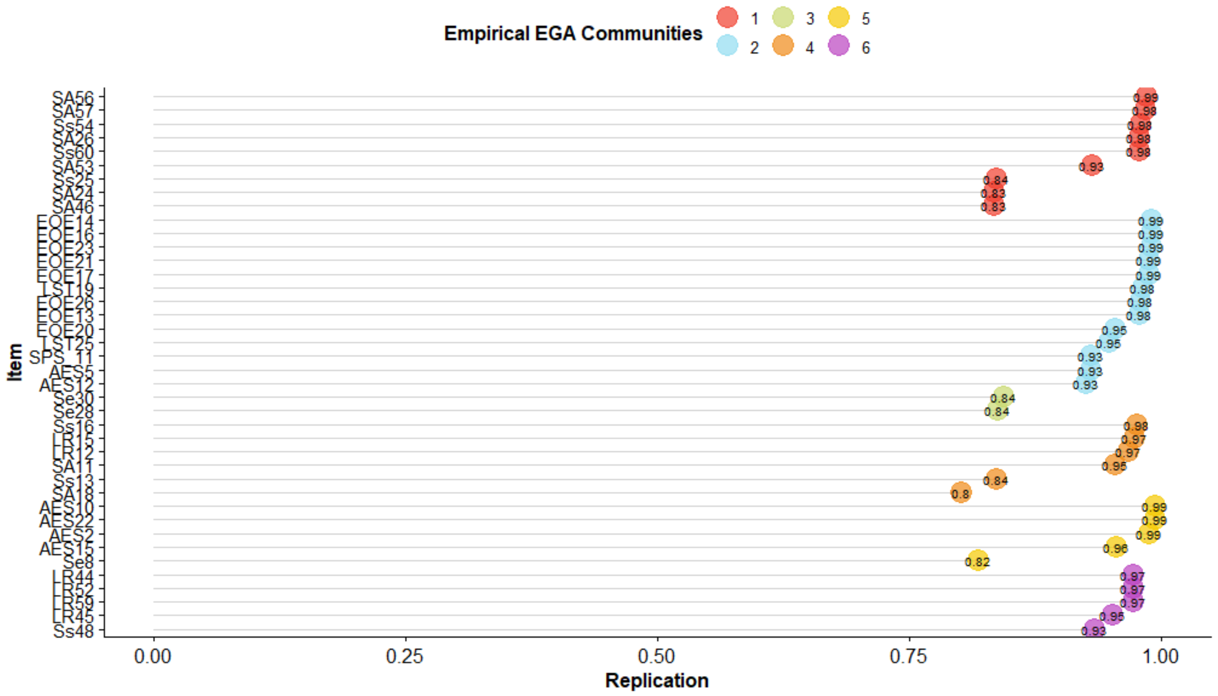

Note. N = 187. SPS = items that previous studies did not assign to any subscale of the Highly sensitive person scale; EOE = Ease of Excitation; AES = Aesthetic Sensitivity; LST = Low Sensory Threshold; Ss = Sensory Sensitivity; SA = Sensory Avoiding; Se = Sensation Seeking; LR = Low Registration.

Figure S15

Sample of HSPs Facebook members: Network Plot of the Highly Sensitive Person Scale (HSPS) and the Adolescent Adult Sensory Profile(ASP)

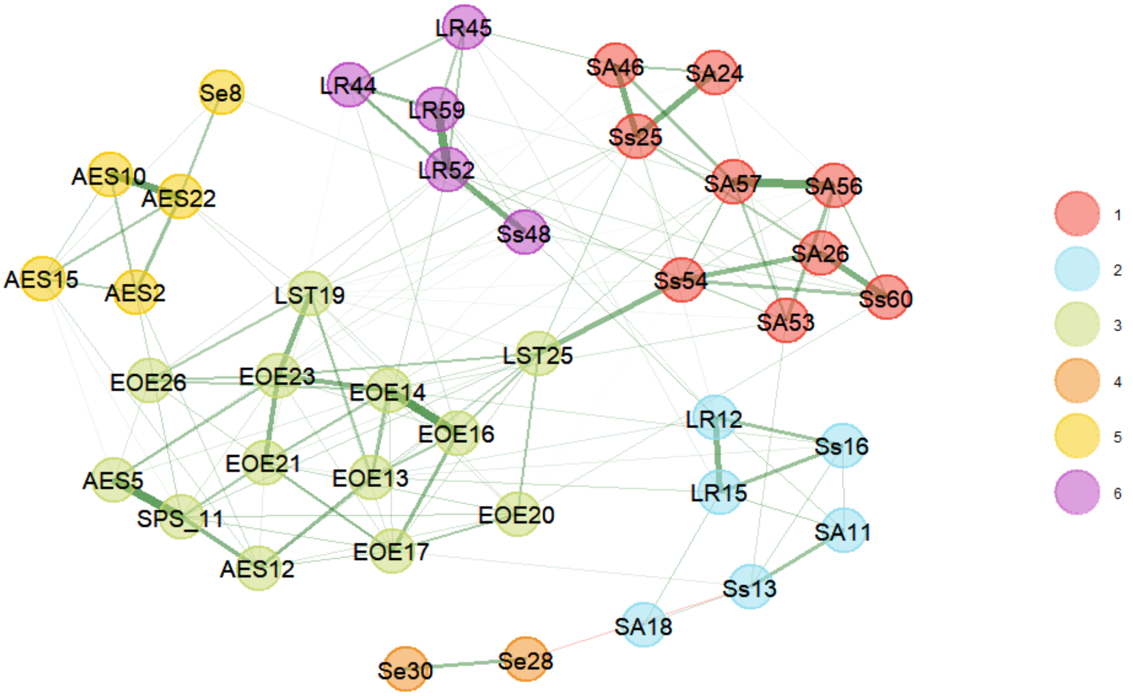

Note. *N* = 187. SPS = items that previous studies did not assign to any subscale of the Highly sensitive person scale; EOE = Ease of Excitation; AES = Aesthetic Sensitivity; LST = Low Sensory Threshold; Ss = Sensory Sensitivity; SA = Sensory Avoiding; Se = Sensation Seeking; LR = Low Registration.
